# Iron(III)-Catalyzed Highly Regioselective Halogenation of 8-Amidoquinolines in Water

**DOI:** 10.3390/molecules24030535

**Published:** 2019-02-01

**Authors:** Yang Long, Lei Pan, Xiangge Zhou

**Affiliations:** College of Chemistry, Sichuan University, Chengdu 610064, China; elitelyhere@163.com (Y.L.); panleiscu@163.com (L.P.)

**Keywords:** 8-amidoquinoline, iron, catalysis, water

## Abstract

A simple protocol of iron(III)-catalyzed halogenation of 8-amidoquinolines in water under mild conditions was developed, affording the 5-halogenlated products in good to excellent yields up to 98%. The reaction mechanism most likely involves a single-electron transfer (SET) process.

## 1. Introduction

**Scheme 1:**
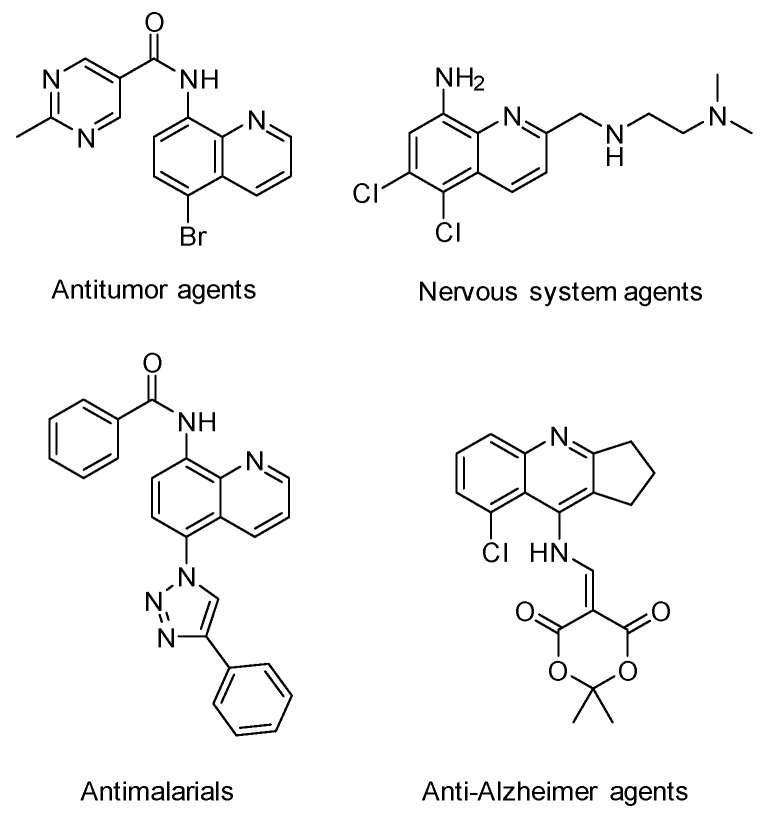
Examples of biologically active compounds containing quinoline motifs.

Quinolines are an important kind of structural motif found in numerous bioactive molecules and natural products with applications in pharmaceutical chemistry. Over the past decades, the quinoline framework has drawn significant attention due to its frequent occurrence in bioactive natural products, agrochemicals, and functional materials (Scheme 1) [1,2,3,4,5,6,7,8]. In addition, with the seminal achievement of using 8-amidoquinoline as a bidentate directing group by Daugulis [9], a great deal of C-H functionalization reaction has been realized [10]. Therefore, the development of facile and efficient methods to halogenate quinolines is highly necessary. However, compared with well-developed functionalization of quinoline’s C2, C3, and C8 positions [11,12,13,14,15,16,17,18,19], the approaches to the C5-functionalization are still less studied.

**Scheme 2:**
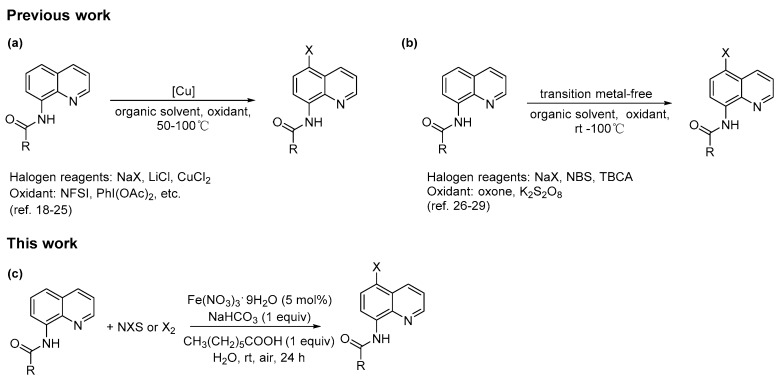
C5 halogenation of 8-amidoquinoline amides. (**a**) Cu-catalyzed halogenation of 8-amidoquinoline amides; (**b**) transition metal-free halogenation of 8-amidoquinoline amides; (**c**) Fe(III)-catalyzed halogenation of 8-amidoquinoline amides.

Recently, Stahl and co-workers reported Cu(I) catalyzed C5 chlorination of 8-amidoquinoline amides by using LiCl as a chlorination reagent [20]. Similarly, Zhang, Li, Huang, etc. described copper-catalyzed C5 C-H halogenation of 8-amidoquinolines (Scheme 2a) [21,22,23,24,25,26,27]. Meanwhile, Li and other groups also reported transition metal-free C5 halogenation of 8-amidoquinolines (Scheme 2b) [28,29,30,31]. Although approaches to the C5 halogenation have been developed to a certain extent, the work mentioned above still has some drawbacks: (i) usually there was a requirement of some complex oxidants, such as *N*-fluorobis(benzenesulfon)imide (NFSI), PhI(OAc)_2_, oxone, etc.; (ii) the halogen reagents were expensive, low cost resources like Br_2_ or I_2_ that hardly reacted effectively in these systems [25]; (iii) normally there was usage of an organic solvent, while using water as the sole solvent was pretty rare [32]; (iv) some reactions needed harsh conditions, such as high temperature or inert atmosphere. In continuation of our recent work of aqueous catalysis [33,34,35,36,37,38,39,40], herein is reported iron-catalyzed remote C5 C-H-halogenation of 8-amidoquinolines in water by using NXS and X_2_ (X = Br, I)) as effective halogen sources (Scheme 2c). Notably, we think this protocol is environmentally friendly with the following novelties: (i) water as a solvent and air as an oxidant; (ii) cheap iron salt as the catalyst at mild reaction conditions at room temperature [41]; (iii) easily available and budget-friendly halogen reagents.

## 2. Results and Discussion

Initially, *N*-(quinolin-8-yl)pivalamide, *N*-bromo-succinimide (NBS) or Br_2_ were treated as model substrates to optimize the reaction conditions. As shown in Table 1, no desired product was observed under an inert atmosphere (entry 1), indicating the indispensability of the oxidant. Furthermore, although the product could be obtained with 91% yield by NBS without a catalyst, the yield was only 35% with Br_2_ as a halogen source (entry 2). Thus, in order to make both halogen reagents react well, a variety of metal salts including Pd(II), Cu(II), Co(II), and Fe(III) were then evaluated. To our delight, metal salts were helpful for the catalysis, especially in the case of Br_2_ as a halogen source, the reactivity of which was improved to the same level as NBS (entries 3–7). Considering the efficiency, low cost, and environmental friendliness, Fe(NO_3_)_3_·9H_2_O was selected as a catalyst for the further studies. The oxidant was another important factor that affected the results. Simple silver salts such as Ag_2_O and Ag_2_CO_3_ gave similar yields as air (entries 8 and 9), while AgOAc raised the yield to about 75% (entry 10). Interestingly, the yield increased dramatically to about 90% in the case of CH_3_(CH_2_)_5_COOAg (entry 11), indicating the positive effect of a long chain carboxylic acid ion, which might function as a phase transfer reagent [42]. Indeed, when the air was used as an oxidant, the combination of CH_3_(CH_2_)_5_COOH and NaHCO_3_ resulted in the best yield of 95% for both halogen reagents (entry 12). In summary, the optimal conditions consist of quinolines (0.3 mmol), NBS or Br_2_ (0.6 mmol), Fe(NO_3_)_3_·9H_2_O (5 mol%), NaHCO_3_ (0.3 mmol), and CH_3_(CH_2_)_5_COOH (0.3 mmol) at room temperature for 24 h in the air.

**Scheme 3:**
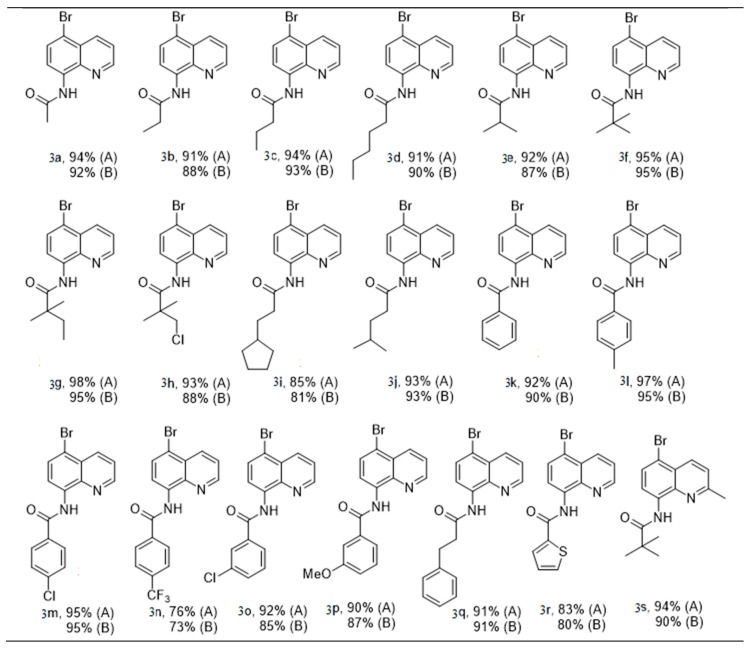
Iron-catalyzed C-H bromination. Reaction conditions A: 1 (0.3 mmol), NBS (0.6 mmol), CH_3_(CH_2_)_5_COOH (0.3 mmol), NaHCO_3_ (0.3 mmol), and Fe(NO_3_)_3_·9H_2_O (5 mol%) in water (1 mL) at rt for 24 h. Reaction conditions B: 1 (0.3 mmol), Br_2_ (0.6 mmol), CH_3_(CH_2_)_5_COOH (0.3 mmol), NaHCO_3_ (0.3 mmol), and Fe(NO_3_)_3_·9H_2_O (5 mol%) in water (1 mL) at rt for 24 h.

**Figure 1:**
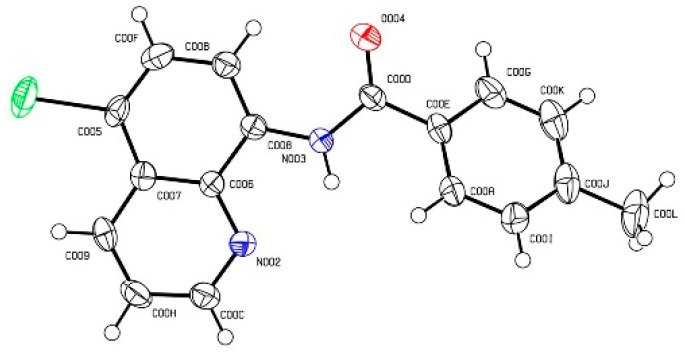
Single-crystal X-ray structure of **2l**.

With the optimized conditions in hand, we subsequently examined the scope of quinoline derivatives, as shown in Scheme 3. Overall, different substrates provided moderate to excellent yields, and both halogen reagents could efficiently realize the reaction, while much lower reactive activity was found in the previous reports [26]. The length of the linear alkyl chain showed few effects on the reaction, affording similar results around 90% yields (Scheme 3a–d). Various branched chain alkyl groups also gave excellent yield (Scheme 3e–j). Meanwhile, different aryl groups were also compatible in this system. The substrates bearing *para*-methyl chloro groups gave excellent yields up to 95% (Scheme 3l,m), *para*-trifluoromethyl groups gave a moderate yield of about 73% (Scheme 3n), and the meta-chloro and methoxyl group gave a good yield (Scheme 3o,p). Replacement of the aryl with the ethylphenyl and thienyl groups were also well-tolerated (Scheme 3q,r). The methyl group on the C2 position of the quinoline ring were also compatible (Scheme 3s). Moreover, the structure of the product (Scheme 3l) was confirmed by X-ray crystallography (Figure 1) [CCDC 1480727, for detailed crystal data, see Supplementary Information (SI)].

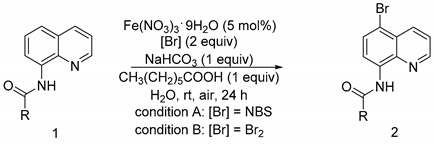



**Scheme 4:**
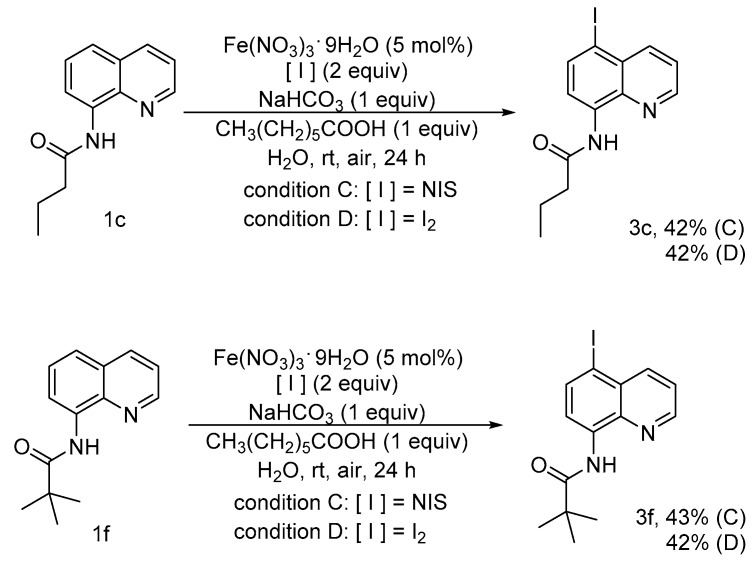
Iron-catalyzed C-H halogenation. Reaction conditions C: 1 (0.3 mmol), NIS (0.6 mmol), CH_3_(CH_2_)_5_COOH (0.3 mmol), NaHCO_3_ (0.3 mmol), and Fe(NO_3_)_3_·9H_2_O (5 mol%) in water (1 mL) at rt for 24 h. Reaction conditions D: 1 (0.3 mmol), I_2_ (0.6 mmol), CH_3_(CH_2_)_5_COOH (0.3 mmol), NaHCO_3_ (0.3 mmol), and Fe(NO_3_)_3_·9H_2_O (5 mol%) in water (1 mL) at rt for 24 h.

In an endeavor to expand the scope of this methodology, NIS and I_2_ were treated as halogen reagents. As shown in Scheme 4, iodination reaction could also be fulfilled (although with low yields around 40%) by using I_2_ or NIS (3c, 3f). The lower yield of iodination than that of bromination might have been due to the lower reactivity of the iodine free radical and the instability of iodo products [43,44].

**Scheme 5:**
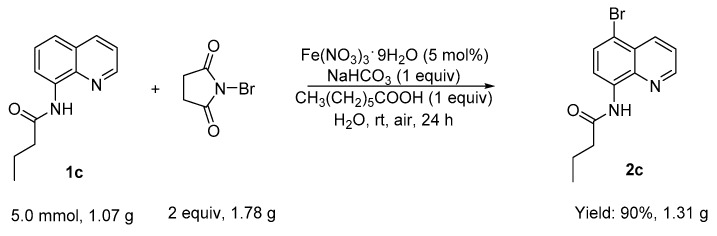
Gram-scale iron-catalyzed C-H bromination of **1c**.

Furthermore, the scaled-up reaction was carried out, giving quantities of the 2c in 90% (Scheme 5), which indicated it as a facile route to the desired product on a more synthetically useful scale.

**Scheme 6:**
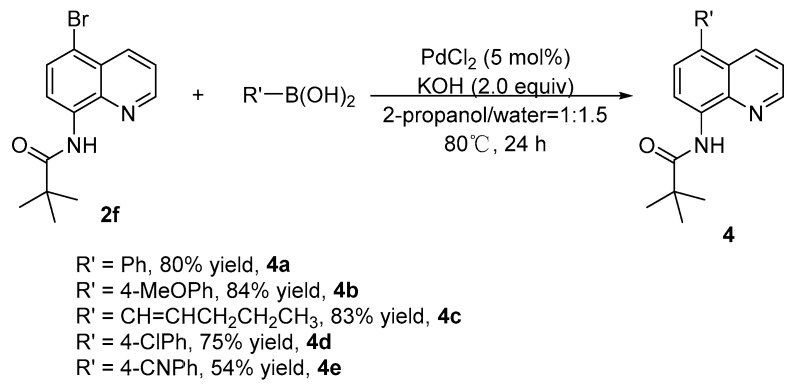
Suzuki coupling reaction.

In order to expand the application of this protocol, *N*-(5-bromoquinolin-8-yl)pivalamide was reacted with boronic acid to give a series of derivatives by simple Suzuki coupling reactions in moderate to good yields ranging from 54% to 84%(Scheme 6) [45,46].

**Scheme 7:**
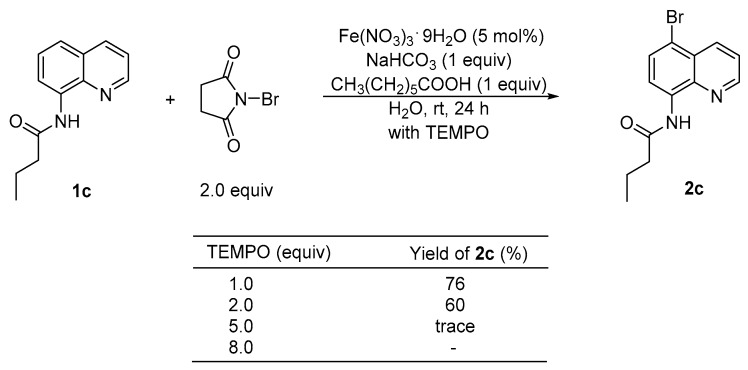
Radical trapping experiments.

Next, radical trapping experiments were carried out, as shown in Scheme 7, and the addition of 2,2,6,6-tetramethyl-1-piperidinyloxy (TEMPO) drastically hampered the reaction. In addition, an EPR experiment was done (for detailed EPR spectra, see SI). Both results suggested that the radical mechanism might be involved in the reaction.

**Scheme 8:**
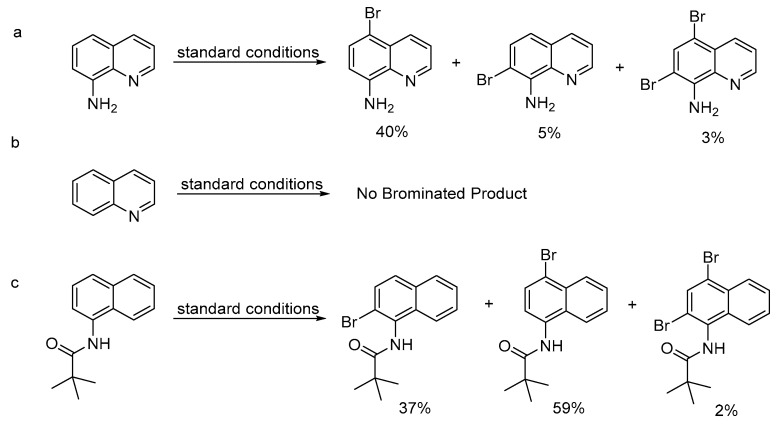
The exploration of the analogous substrates.

At last, three analogous substrates were also investigated. As shown in Scheme 8, 8-aminoquinoline and quinoline gave 40% and none of the product, respectively, which suggested the necessity of the protected amino group during the reaction (Scheme 8a). Moreover, *N*-(1-naphthyl)carboxamide generated a mixture of brominated byproducts, indicating that a chelation of iron with *N*,*N*-bidentate 8-aminoquinoline might play a predominant role in the reaction (Scheme 8c).

**Scheme 9:**
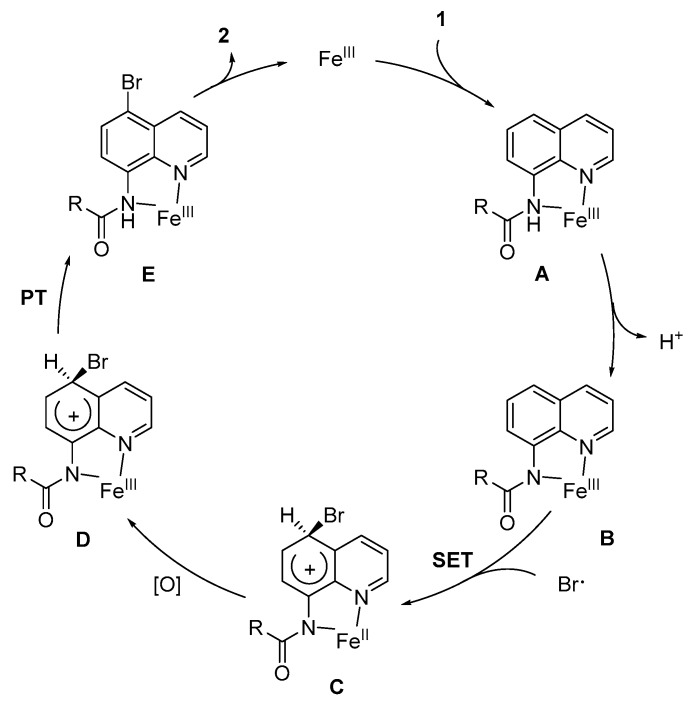
Plausible mechanism for C5 halogenation of quinolines.

Based on our work, as well as existing literatures [20,21,22,23,24,25,26,27], a plausible reaction pathway was proposed, as shown in Scheme 9. At first, substrate 1 and Fe(III) species formed complex A, which was transformed to be complex B after deprotonation [41,47]. Then, B, which may have influenced the electron density of the quinoline ring at the C5-H position [47,48], was attacked by a bromine radical from the halogen reagent to form complex C by a single electron transfer process (SET). The complex C soon transformed into D through oxidation. After generation of the intermediate E through the proton transfer process (PT), a metal dissociation process gained the terminal product 2 and Fe(III) species, and the catalytic cycle was completed. Furthermore, considering that the reaction could be carried out without a catalyst, although the yield was low, a metal-free halogenation mechanism reported by Xu [32] also may have been involved in the reaction.

## 3. Materials and Methods

### 3.1. General Experimental Procedures

Unless otherwise noted, all the reactions were performed under air atmosphere. All reagents were used without purification as commercially available. All reactions were monitored by thin layer chromatography. Analytical thin layer chromatography (TLC) was performed using silica gel GF_254_ plates. Chemical yields refer to pure isolated substances. Column chromatography was performed using silica gel (200–300 mesh or 300–400 mesh) eluting with petroleum ether and ethyl acetate. All products were characterized by their NMR spectra. ^1^H-NMR spectra were recorded at 400 MHz and ^13^C-NMR spectra at 100 MHz (Bruker DPX, Bruker, Madison, WI, USA) with CDCl_3_ as a solvent. Chemical shifts were reported in ppm using TMS as the internal standard.

### 3.2. Synthesis of Starting Materials

To a 50 mL single neck flask charged with CH_2_Cl_2_ (20 mL) was added 8-aminoquinoline (10 mmol) and triethylamine (11 mmol) and stirred at room temperature for 5 min, then the reaction solution was cooled in an ice bath. The acid chloride (12 mmol) was added dropwise (if solid, it was dissolved with CH_2_Cl_2_). The reaction solution was stirred overnight. When it was completed monitored by TLC, the mixture was filtered through a pad of Celite, the solid was washed with ethyl acetate (30 mL), and the organic layer was washed with 1 M NaHCO_3_ of aqueous solution (3 × 15 mL), then the organic layer was dried with Na_2_SO_4_, filtered, and the solvent was removed under reduced pressure. The product was finally obtained by column chromatography on silica gel (PE/EtOAc = 20/1).

### 3.3. General Procedures for Iron-Catalyzed Halogenation C5-H of 8-Amidoquinolines under Mild Conditions in Water

Reaction conditions A: A mixture of 1 (0.3 mmol), NBS (0.6 mmol), Fe(NO_3_)_3_·9H_2_O(5 mol%), CH_3_(CH_2_)_5_COOH (0.3 mmol), NaHCO_3_ (0.3 mmol) in water (1.0 mL) in a 20 mL Schlenk tube was stirred at room temperature for 24 h. Then, the mixture was extracted with EtOAc (10 mL × 4). The combined organic layer was dried with Na_2_SO_4_ and filtered through a pad of Celite. The solvent was removed under reduced pressure and the residue was purified by silica gel column chromatography using PE/EtOAc (20/1) as an eluent to afford the products.

Reaction conditions B: A mixture of 1 (0.3 mmol), Br_2_ (0.6 mmol), Fe(NO_3_)_3_·9H_2_O (5 mol%), CH_3_(CH_2_)_5_COOH (0.3 mmol), NaHCO_3_ (0.3 mmol) in water (1.0 mL) in a 20 mL Schlenk tube was stirred at room temperature for 24 h. Then, the mixture was extracted with EtOAc (10 mL × 4). The combined organic layer was dried with Na_2_SO_4_ and filtered through a pad of Celite. The solvent was removed under reduced pressure and the residue was purified by silica gel column chromatography using PE/EtOAc (20/1) as an eluent to afford the corresponding halogenation products.

Reaction conditions C: A mixture of 1 (0.3 mmol), NIS (0.6 mmol), Fe(NO_3_)_3_·9H_2_O(5 mol%), CH_3_(CH_2_)_5_COOH (0.3 mmol), NaHCO_3_ (0.3 mmol) in water (1.0 mL) in a 20 mL Schlenk tube was stirred at room temperature for 24 h. Then, the mixture was extracted with EtOAc (10 mL × 4). The combined organic layer was dried with Na_2_SO_4_ and filtered through a pad of Celite. The solvent was removed under reduced pressure and the residue was purified by silica gel column chromatography using PE/EtOAc (20/1) as an eluent to afford the products.

Reaction conditions D: A mixture of 1 (0.3 mmol), I_2_ (0.6 mmol), Fe(NO_3_)_3_·9H_2_O(5 mol%), CH_3_(CH_2_)_5_COOH (0.3 mmol), NaHCO_3_ (0.3 mmol) in water (1.0 mL) in a 20 mL Schlenk tube was stirred at room temperature for 24 h. Then, the mixture was extracted with EtOAc (10 mL × 4). The combined organic layer was dried with Na_2_SO_4_ and filtered through a pad of Celite. The solvent was removed under reduced pressure and the residue was purified by silica gel column chromatography using PE/EtOAc (20/1) as an eluent to afford the products.

*N-(5-Bromoquinolin-8-yl)acetamide* (**2a**), ^1^H-NMR (400 MHz, CDCl_3_) δ = 9.72 (s, 1H), 8.77 (dd, *J* = 4.2, 1.5 Hz, 1H), 8.61 (d, *J* = 8.4 Hz, 1H), 8.46 (dd, *J* = 8.5, 1.6 Hz, 1H), 7.73 (d, *J* = 8.4 Hz, 1H), 7.51 (dd, *J* = 8.5, 4.2 Hz, 1H), 2.34 (s, 3H). ^13^C-NMR (100 MHz, CDCl_3_) δ = 168.78, 148.55, 138.81, 135.90, 134.37, 130.85, 127.05, 122.62, 116.82, 114.10, 25.18. HRMS: calculated [C_11_H_10_BrN_2_O]^+^: 264.9971, found: 264.9964 [26].

*N-(5-Bromoquinolin-8-yl)propionamide* (**2b**), ^1^H-NMR (400 MHz, CDCl_3_) δ = 9.76 (s, 1H), 8.78 (dd, *J* = 4.2, 1.6 Hz, 1H), 8.65 (d, *J* = 8.4 Hz, 1H), 8.47 (dd, *J* = 8.5, 1.6 Hz, 1H), 7.75 (d, *J* = 8.4 Hz, 1H), 7.52 (dd, *J* = 8.5, 4.2 Hz, 1H), 2.59 (q, *J* = 7.6 Hz, 2H), 1.33 (t, *J* = 7.6 Hz, 3H). ^13^C-NMR (100 MHz, CDCl_3_) δ = 172.49, 148.55, 138.94, 135.91, 134.43, 130.90, 127.09, 122.61, 116.82, 113.95, 31.25, 9.68. HRMS: calculated [C_12_H_12_BrN_2_O]^+^: 279.0128, found: 279.0122 [31].

*N-(5-Bromoquinolin-8-yl)butyramide* (**2c**), ^1^H-NMR (400 MHz, CDCl_3_) δ = 9.75 (s, 1H), 8.78 (dd, *J* = 4.2, 1.5 Hz, 1H), 8.65 (d, *J* = 8.4 Hz, 1H), 8.47 (dd, *J* = 8.5, 1.5 Hz, 1H), 7.74 (d, *J* = 8.4 Hz, 1H), 7.52 (dd, *J* = 8.5, 4.2 Hz, 1H), 2.56–2.50 (m, 2H), 1.90–1.78 (m, 2H), 1.05 (t, *J* = 7.4 Hz, 3H). ^13^C-NMR (100 MHz, CDCl_3_) δ = 171.75, 148.50, 138.87, 135.92, 134.38, 130.88, 127.06, 122.58, 116.84, 113.94, 40.11, 19.05, 13.83. HRMS: calculated [C_13_H_14_BrN_2_O]^+^: 293.0284, found: 293.0284 [26].

*N-(5-Bromoquinolin-8-yl)hexanamide* (**2d**), ^1^H-NMR (400 MHz, CDCl_3_) δ = 9.74 (s, 1H), 8.77 (dd, *J* = 4.2, 1.6 Hz, 1H), 8.65 (d, *J* = 8.4 Hz, 1H), 8.46 (dd, *J* = 8.5, 1.6 Hz, 1H), 7.74 (d, *J* = 8.4 Hz, 1H), 7.51 (dd, *J* = 8.5, 4.2 Hz, 1H), 2.62–2.44 (m, 2H), 1.90–1.74 (m, 2H), 1.45–1.32 (m, 4H), 0.91 (t, *J* = 7.1 Hz, 3H). ^13^C-NMR (100 MHz, CDCl_3_) δ = 171.89, 148.52, 138.89, 135.83, 134.42, 130.85, 127.03, 122.57, 116.79, 113.92, 38.19, 31.45, 25.27, 22.48, 13.99. HRMS: calculated [C_15_H_18_BrN_2_O]^+^: 321.0597, found: 321.0587.

*N-(5-Bromoquinolin-8-yl)isobutyramide* (**2e**), ^1^H-NMR (400 MHz, CDCl_3_) δ = 9.86 (s, 1H), 8.80 (d, *J* = 4.2 Hz, 1H), 8.67 (d, *J* = 8.4 Hz, 1H), 8.48 (d, *J* = 8.5 Hz, 1H), 7.76 (d, *J* = 8.4 Hz, 1H), 7.53 (dd, *J* = 8.5, 4.2 Hz, 1H), 2.76 (dt, *J* = 13.8, 6.9 Hz, 1H), 1.35 (d, *J* = 6.9 Hz, 6H). ^13^C-NMR (100 MHz, CDCl_3_) δ = 175.73, 148.56, 139.07, 135.92, 134.49, 130.89, 127.09, 122.58, 116.86, 113.93, 37.12, 19.66. HRMS: calculated [C_13_H_14_BrN_2_O]^+^: 293.0284, found: 293.0284 [28].

*N-(5-Bromoquinolin-8-yl)pivalamide* (**2f**), ^1^H-NMR (400 MHz, CDCl_3_) δ = 10.13 (s, 1H), 8.71 (dd, *J* = 4.2, 1.6 Hz, 1H),8.59 (d, *J* = 8.4 Hz, 1H), 8.37 (dd, *J* = 8.5, 1.6 Hz, 1H), 7.67 (d, *J* = 8.4 Hz, 1H), 7.43 (dd, *J* = 8.5, 4.2 Hz, 1H), 1.33 (s, 9H). ^13^C-NMR (100 MHz, CDCl_3_) δ = 176.19, 147.60, 138.34, 134.79, 133.53, 129.83, 126.02, 121.50, 115.62, 112.82, 39.32, 26.64. HRMS: calculated [C_14_H_15_BrN_2_NaO]^+^: 329.0260, found: 329.0253 [26].

*N-(5-Bromoquinolin-8-yl)-2,2-dimethylbutanamide* (**2g**), ^1^H-NMR (400 MHz, CDCl_3_) δ = 10.10 (s, 1H), 8.71 (d, *J* = 4.2 Hz, 1H), 8.60 (d, *J* = 8.4 Hz, 1H), 8.44–8.30 (m, 1H), 7.67 (d, *J* = 8.4 Hz, 1H), 7.43 (ddd, *J* = 8.5, 4.2, 0.7 Hz, 1H), 1.66 (q, *J* = 7.5 Hz, 2H), 1.29 (s, 6H), 0.85 (t, *J* = 7.5 Hz, 3H). ^13^C-NMR (100 MHz, CDCl_3_) δ = 175.62, 147.62, 138.35, 134.80, 133.51, 129.85, 126.04, 121.50, 115.61, 112.78, 43.05, 33.04, 23.99, 8.26. HRMS: calculated [C_15_H_18_BrN_2_O]^+^: 321.0597, found: 321.0587.

*N-(5-Bromoquinolin-8-yl)-3-chloro-2,2-dimethylpropanamide* (**2h**), ^1^H-NMR (400 MHz, CDCl_3_) δ = 10.21 (s, 1H), 8.71 (dd, *J* = 4.2, 1.6 Hz, 1H), 8.58 (d, *J* = 8.4 Hz, 1H), 8.36 (dd, *J* = 8.5, 1.6 Hz, 1H), 7.67 (d, *J* = 8.4 Hz, 1H), 7.43 (dd, *J* = 8.5, 4.2 Hz, 1H), 3.69 (s, 2H), 1.45 (s, 6H). ^13^C-NMR (100 MHz, CDCl_3_) δ = 172.42, 147.77, 138.28, 134.81, 132.99, 129.74, 126.02, 121.62, 115.89, 113.44, 51.65, 44.79, 22.61. HRMS: calculated [C_14_H_14_BrClN_2_NaO]^+^: 362.9870, found: 362.9869.

*N-(5-Bromoquinolin-8-yl)-3-cyclopentylpropanamide* (**2i**), ^1^H-NMR (400 MHz, CDCl_3_) δ = 9.76 (s, 1H), 8.84–8.76 (m, 1H), 8.66 (d, *J* = 8.4 Hz, 1H), 8.48 (dt, *J* = 8.5, 1.4 Hz, 1H), 7.76 (d, *J* = 8.4 Hz, 1H), 7.53 (ddd, *J* = 8.5, 4.2, 0.9 Hz, 1H), 2.61–2.53 (m, 2H), 1.88–1.78 (m, 5H), 1.69–1.47 (m, 4H), 1.17 (tt, *J* = 14.0, 6.8 Hz, 2H). ^13^C-NMR (100 MHz, CDCl_3_) δ = 172.02, 148.55, 138.93, 135.90, 134.46, 130.90, 127.08, 122.59, 116.84, 113.94, 39.74, 37.55, 32.55, 31.77, 25.20. HRMS: calculated [C_17_H_19_BrN_2_NaO]^+^: 369.0573, found: 369.0566.

*N-(5-Bromoquinolin-8-yl)-4-methylpentanamide* (**2j**), ^1^H-NMR (400 MHz, CDCl_3_) δ = 9.66 (s, 1H), 8.69 (dd, *J* = 4.2, 1.4 Hz, 1H), 8.55 (d, *J* = 8.4 Hz, 1H), 8.37 (dd, *J* = 8.5, 1.5 Hz, 1H), 7.65 (d, *J* = 8.4 Hz, 1H), 7.42 (dd, *J* =8.5, 4.2 Hz, 1H), 2.54–2.40 (m, 2H), 1.68–1.55 (m, 3H), 0.88 (d, *J* = 6.4 Hz, 6H). ^13^C-NMR (100 MHz, CDCl_3_) δ = 172.06, 148.54, 138.91, 135.91, 134.44, 130.90, 127.07, 122.59, 116.85, 113.94, 36.24, 34.34, 27.83, 22.41. HRMS: calculated [C_15_H_18_BrN_2_O]^+^: 321.0597, found: 321.0588 [32].

*N-(5-Bromoquinolin-8-yl)benzamide* (**2k**), ^1^H-NMR (400 MHz, CDCl_3_) δ = 10.65 (s, 1H), 8.87–8.74 (m, 2H), 8.47 (dd, *J* = 8.5, 1.5 Hz, 1H), 8.10–8.00 (m, 2H), 7.79 (d, *J* = 8.4 Hz, 1H), 7.61–7.49 (m, 4H). ^13^C-NMR (100 MHz, CDCl_3_) δ = 165.48, 148.78, 139.42, 136.16, 134.86, 134.51, 132.07, 131.05, 128.88, 127.33, 122.79, 117.13, 114.48. HRMS: calculated [C_16_H_12_BrN_2_O]^+^: 327.0128, found: 327.0120 [26].

*N-(5-Bromoquinolin-8-yl)-4-methylbenzamide* (**2l**), 1H-NMR (400 MHz, CDCl3) δ = 10.62 (s, 1H), 8.89–8.72 (m, 2H), 8.46 (dd, *J* = 8.5, 1.5 Hz, 1H), 7.93 (d, *J* = 8.2 Hz, 2H), 7.78 (d, *J* = 8.4 Hz, 1H), 7.52 (dd, *J* = 8.5, 4.2 Hz, 1H), 7.31 (d, *J* = 7.9 Hz, 2H), 2.43 (s, 3H). ^13^C-NMR (100 MHz, CDCl_3_) δ = 165.48, 148.71, 142.62, 139.41, 136.17, 134.62, 132.04, 131.08, 129.54, 127.34, 127.30, 122.74, 117.10, 114.29, 21.61. HRMS: calculated [C_17_H_14_BrN_2_O]^+^: 341.0284, found: 341.0284 [24].

*N-(5-Bromoquinolin-8-yl)-4-chlorobenzamide* (**2m**), ^1^H-NMR (400 MHz, CDCl_3_) δ = 10.63 (s, 1H), 8.84 (dd, *J* = 4.2, 1.6 Hz, 1H), 8.76 (d, *J* = 8.4 Hz, 1H), 8.51 (dd, *J* = 8.5, 1.6 Hz, 1H), 7.98 (d, *J* = 8.6 Hz, 2H), 7.81 (d, *J* = 8.4 Hz, 1H), 7.57 (dd, *J* = 8.5, 4.2 Hz, 1H), 7.52–7.49 (m, 2H). ^13^C-NMR (100 MHz, CDCl_3_) δ = 164.33, 148.82, 139.31, 138.35, 136.21, 134.22, 133.20, 131.00, 129.13, 128.73, 127.30, 122.83, 117.18, 114.72. HRMS: calculated [C_16_H_11_BrClN_2_O]^+^: 360.9738, found: 360.9725 [32].

*N-(5-Bromoquinolin-8-yl)-4-(trifluoromethyl)benzamide* (**2n**), ^1^H-NMR (400 MHz, CDCl_3_) δ = 10.70 (s, 1H), 8.83 (dd, *J* = 4.2, 1.5 Hz, 1H), 8.76 (d, *J* = 8.4 Hz, 1H), 8.51 (dd, *J* = 8.5, 1.5 Hz, 1H), 8.14 (d, *J* = 8.2 Hz, 2H), 7.80 (dd, *J* = 8.4, 3.4 Hz, 3H), 7.57 (dd, *J* = 8.5, 4.2 Hz, 1H).. ^13^C-NMR (100 MHz, CDCl_3_) δ = 163.92, 148.91, 139.23, 138.00, 136.14, 133.98, 133.78, 130.91, 127.75, 127.24, 125.91 (q, *J* = 3.7 Hz), 122.89, 122.33, 117.20, 115.02. HRMS: calculated [C_17_H_10_BrF_3_N_2_NaO]^+^: 416.9821, found: 416.9817 [31].

*N-(5-Bromoquinolin-8-yl)-3-chlorobenzamide* (**2o**), ^1^H-NMR (400 MHz, CDCl_3_) δ = 10.66 (s, 1H), 8.88 (dd, *J* = 4.2, 1.6 Hz, 1H), 8.79 (d, *J* = 8.4 Hz, 1H), 8.55 (dd, *J* = 8.5, 1.6 Hz, 1H), 8.04 (t, *J* = 1.8 Hz, 1H), 7.96–7.89 (m, 1H), 7.85 (d, *J* = 8.4 Hz, 1H), 7.66–7.53 (m, 2H), 7.49 (t, *J* = 7.8 Hz, 1H). ^13^C-NMR (100 MHz, CDCl_3_) δ = 162.94, 147.88, 138.32, 135.58, 135.08, 134.06, 133.12, 131.03, 129.91, 129.11, 126.66, 126.24, 124.23, 121.82, 116.14, 113.80. HRMS: calculated [C_16_H_10_BrClN_2_O]^+^: 360.9743, found: 360.9726 [24].

*N-(5-Bromoquinolin-8-yl)-3-methoxylbenzamide* (**2p**), ^1^H-NMR (400 MHz, CDCl_3_) δ = 10.70 (s, 1H), 9.01–8.73 (m, 2H), 8.55 (dd, *J* = 8.5, 1.6 Hz, 1H), 7.85 (d, *J* = 8.4 Hz, 1H), 7.66–7.57 (m, 3H), 7.46 (t, *J* = 8.2 Hz, 1H), 7.13 (ddd, *J* = 8.3, 2.5, 1.0 Hz, 1H), 3.92 (s, 3H). ^13^C-NMR (100 MHz, CDCl_3_) δ = 165.30, 160.04, 148.84, 139.45, 136.35, 136.06, 134.50, 131.00, 129.86, 127.28, 122.79, 119.06, 118.19, 117.04, 114.50, 112.71, 55.55. HRMS: calculated [C_17_H_14_BrN_2_O_2_]^+^: 357.0239, found: 357.0217 [24].

*N-(5-Bromoquinolin-8-yl)-3-phenylpropanamide* (**2q**), ^1^H-NMR (400 MHz, CDCl_3_) δ = 9.72 (s, 1H), 8.74 (dd, *J* = 4.2, 1.6 Hz, 1H), 8.65 (d, *J* = 8.4 Hz, 1H), 8.46 (dd, *J* = 8.5, 1.6 Hz, 1H), 7.75 (d, *J* = 8.4 Hz, 1H), 7.50 (dd, *J* = 8.5, 4.2 Hz, 1H), 7.31–7.27 (m, 4H), 7.24–7.17 (m, 1H), 3.17–3.10 (m, 2H), 2.87 (dd, *J* = 8.7, 7.0 Hz, 2H). ^13^C-NMR (100 MHz, CDCl_3_) δ = 170.77, 148.55, 140.66, 138.88, 135.88, 134.30, 130.88, 128.62, 128.44, 127.07, 126.34, 122.64, 116.92, 114.13, 39.71, 31.40. HRMS: calculated [C_18_H_15_BrN_2_NaO]^+^: 377.0260, found: 377.0256 [22].

*N-(5-Bromoquinolin-8-yl)thiophene-2-carboxamide* (**2r**), ^1^H-NMR (400 MHz, CDCl_3_) δ = 10.58 (s, 1H), 8.89 (dd, *J* = 4.2, 1.5 Hz, 1H), 8.75 (d, *J* = 8.4 Hz, 1H), 8.58 (dd, *J* = 8.5, 1.5 Hz, 1H), 7.86 (dd, *J* = 4.8, 3.7 Hz, 2H), 7.65–7.53 (m, 2H), 7.20 (dd, *J* = 4.9, 3.8 Hz, 1H). ^13^C-NMR (100 MHz, CDCl_3_) δ = 159.95, 148.77, 139.73, 139.11, 136.04, 134.20, 131.19, 130.97, 128.60, 127.92, 127.23, 122.76, 117.00, 114.44. HRMS: calculated [C_14_H_10_BrN_2_OS]^+^: 332.9692, found: 332.9691 [24].

*N-(5-Bromo-2-methyl-8-quinolinyl)- pivalamide* (**2s**), ^1^H-NMR (400 MHz, CDCl_3_) δ = 10.31 (s, 1H), 8.63 (d, *J* = 8.4 Hz, 1H), 8.35 (d, *J* = 8.6 Hz, 1H), 7.69 (d, *J* = 8.4 Hz, 1H), 7.39 (d, *J* = 8.6 Hz, 1H), 2.76 (s, 3H), 1.42 (s, 6H). ^13^C-NMR (100 MHz, CDCl_3_) δ = 177.15, 157.71, 138.78, 135.93, 133.89, 129.85, 125.31, 123.34, 116.62, 113.85, 40.39, 27.64, 25.17. HRMS: calculated [C_15_H_18_BrN_2_O]^+^: 321.0603, found: 321.0601 [30].

*N-(5-Iodoquinolin-8-yl)butyramide* (**3c**), ^1^H-NMR (400 MHz, CDCl_3_) δ = 9.79 (s, 1H), 8.77 (dd, *J* = 4.2, 1.5 Hz, 1H), 8.57 (d, *J* = 8.3 Hz, 1H), 8.37 (dd, *J* = 8.5, 1.5 Hz, 1H), 8.07 (d, *J* = 8.3 Hz, 1H), 7.53 (dd, *J* = 8.5, 4.2 Hz, 1H), 2.56–2.52 (m, 2H), 1.85 (dd, *J* = 14.9, 7.4 Hz, 2H), 1.06 (t, *J* = 7.4 Hz, 3H). ^13^C-NMR (100 MHz, CDCl_3_) δ = 171.79, 148.65, 140.67, 138.88, 138.25, 135.42, 129.51, 123.10, 117.76, 89.06, 40.17, 19.06, 13.84. HRMS: calculated [C_13_H_14_IN_2_O]^+^: 341.0145, found: 341.0139 [26].

*N-(5-Iodoquinolin-8-yl)pivalamide* (**3f**), ^1^H-NMR (400 MHz, CDCl_3_) δ = 10.26 (s, 1H), 8.76 (dd, *J* = 4.2, 1.5 Hz, 1H), 8.56 (d, *J* = 8.3 Hz, 1H), 8.33 (dd, *J* = 8.5, 1.6 Hz, 1H), 8.05 (d, *J* = 8.3 Hz, 1H), 7.51 (dd, *J* = 8.5, 4.2 Hz, 1H), 1.42 (s, 9H). ^13^C-NMR (100 MHz, CDCl_3_) δ = 176.27, 147.72, 139.59, 138.26, 137.19, 134.49, 128.43, 122.03, 116.52, 87.94, 39.36, 26.63. HRMS: calculated [C_14_H_16_IN_2_O]^+^: 355.0302, found: 355.0292 [31].

### 3.4. General Procedures for Suzuki Coupling Reaction of N-(5-Bromoquinolin-8-yl)pivalamide (***4a*** as an Example)

A mixture of **2f** (0.3 mmol), phenylboronic acid (0.36 mmol), PdCl_2_ (5 mol%), KOH (0.6 mmol), in solvent (2-propanol/water = 1/1.5, 1.0 mL) in a 20 mL Schlenk tube was stirred at 80 °C for 24 h. Then, the mixture was extracted with EtOAc (10 mL × 4). The combined organic layer was dried with Na_2_SO_4_ and filtered through a pad of Celite. The solvent was removed under reduced pressure and the residue was purified by silica gel column chromatography using PE/EtOAc (20/1) as an eluent to afford the products.

*N-(5-Phenylquinolin-8-yl)pivalamide* (**4a**), ^1^H-NMR (400 MHz, CDCl_3_) δ = 10.38 (s, 1H), 9.00–8.71 (m, 2H), 8.29 (dd, *J* = 8.5, 1.6 Hz, 1H), 7.54–7.39 (m, 7H), 1.46 (s, 9H). ^13^C-NMR (101 MHz, CDCl_3_) δ = 177.34, 148.04, 139.27, 138.81, 134.76, 134.11, 134.03, 130.14, 128.52, 128.02, 127.45, 126.27, 121.51, 115.81, 40.42, 27.80. HRMS: calculated [C_20_H_21_N_2_O]^+^: 305.1648, found: 305.1626 [30].

*N-(5-(4-Methoxyphenyl)quinolin-8-yl)pivalamide* (**4b**), ^1^H-NMR (400 MHz, CDCl_3_) δ = 10.35 (s, 1H), 8.83 (dd, *J* = 4.8, 3.2 Hz, 2H), 8.30 (dd, *J* = 8.5, 1.6 Hz, 1H), 7.49 (d, *J* = 8.0 Hz, 1H), 7.41 (dd, *J* = 8.5, 4.2 Hz, 1H), 7.39–7.35 (m, 2H), 7.05–7.00 (m, 2H), 3.89 (s, 3H), 1.45 (s, 9H). ^13^C-NMR (101 MHz, CDCl_3_) δ = 177.28, 159.12, 147.90, 138.83, 134.93, 133.80, 133.77, 131.65, 131.18, 127.90, 126.51, 121.34, 116.03, 113.96, 55.41, 40.39, 27.80. HRMS: calculated [C_21_H_23_N_2_O_2_]^+^: 355.1754, found: 355.1736 [30].

*N-(5-(Pent-1-en-1-yl)quinolin-8-yl)pivalamide* (**4c**), ^1^H-NMR (400 MHz, CDCl_3_) δ = 10.30 (s, 1H), 8.80 (dd, *J* = 4.2, 1.6 Hz, 1H), 8.75 (d, *J* = 8.1 Hz, 1H), 8.47 (dd, *J* = 8.6, 1.6 Hz, 1H), 7.62 (d, *J* = 8.1 Hz, 1H), 7.46 (dd, *J* = 8.5, 4.2 Hz, 1H), 6.96 (d, *J* = 15.6 Hz, 1H), 6.23 (dt, *J* = 15.5, 7.0 Hz, 1H), 2.29 (m, *J* = 7.2, 1.5 Hz, 2H), 1.62–1.51 (m, 2H), 1.43 (d, *J* = 1.6 Hz, 9H), 1.00 (t, *J* = 7.4 Hz, 3H). ^13^C-NMR (100 MHz, CDCl_3_) δ = 177.14, 147.84, 138.72, 134.26, 133.70, 132.79, 129.66, 125.77, 125.26, 124.41, 121.12, 116.21, 40.35, 35.54, 27.77, 22.57, 13.81. HRMS: calculated [C_19_H_25_N_2_O]^+^: 297.1961, found: 297.1950 [30].

*N-(5-(4-Chlorophenyl)quinolin-8-yl)pivalamide* (**4d**), ^1^H-NMR (400 MHz, CDCl_3_) δ = 10.36 (s, 1H), 8.96–8.71 (m, 2H), 8.23 (dd, *J* = 8.5, 1.6 Hz, 1H), 7.50–7.35 (m, 6H), 1.45 (s, 9H). ^13^C-NMR (101 MHz, CDCl_3_) δ = 177.37, 148.08, 138.76, 137.69, 134.46, 134.42, 133.58, 132.64, 131.38, 128.72, 128.10, 126.16, 121.63, 115.89, 40.42, 27.77. HRMS: calculated [C_20_H_19_ClN_2_NaO]^+^: 361.1078, found: 361.1069 [30].

*N-(5-(4-Cyanophenyl)quinolin-8-yl)pivalamide* (**4e**), ^1^H-NMR (400 MHz, CDCl_3_) δ = 10.38 (s, 1H), 8.90–8.82 (m, 2H), 8.19 (dd, *J* = 8.6, 1.5 Hz, 1H), 7.83–7.74 (m, 2H), 7.61–7.55 (m, 2H), 7.53–7.43 (m, 2H), 1.45 (s, 9H). ^13^C-NMR (101 MHz, CDCl_3_) δ = 177.45, 148.36, 144.13, 138.76, 135.18, 133.90, 132.34, 131.71, 130.82, 128.44, 125.72, 121.98, 118.79, 115.70, 111.28, 40.45, 27.74. HRMS: calculated [C_21_H_19_N_3_NaO]^+^: 352.1420, found: 352.1411 [30].

## 4. Conclusions

In summary, we developed an efficient, economical, and environmentally friendly method for iron(III)-catalyzed C5-H halogenation of quinolines at room temperature in water. Both NXS and X_2_ could effectively function as halogen agents. The air could act as the oxidant. This transformation showed a broad substrate scope, good yield, and well-tolerated functionalization. Mechanism studies suggested that a single electron transfer (SET) mechanism might be involved in the reaction.

## Figures and Tables

**Table 1:**
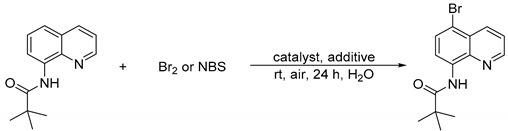
Optimization of the reaction conditions. ^a^

Entry	Catalyst (%)	Additive	Yield [%] ^b^	
			Using Br_2_	Using NBS
1 ^c^	-	-	0	0
2	-	-	35	91
3	Pd(OAc)_2_ (10)	-	47	67
4	Cu(OAc)_2_ (20)	-	43	65
5	Co(OAc)_2_·9H_2_O (20)	-	39	58
6	Fe(NO_3_)_3_·9H_2_O (5)	-	68	68
7	FeCl_3_·9H_2_O (5)	-	63	65
8	Fe(NO_3_)_3_·9H_2_O (5)	Ag_2_O	59	65
9	Fe(NO_3_)_3_·9H_2_O (5)	Ag_2_CO_3_	64	69
10	Fe(NO_3_)_3_·9H_2_O (5)	AgOAc	73	75
11	Fe(NO_3_)_3_·9H_2_O (5)	CH_3_(CH_2_)_5_COOAg	89	90
12	Fe(NO_3_)_3_·9H_2_O (5)	CH_3_(CH_2_)_5_COOH, NaHCO_3_^d^	95	95

^a^ Unless otherwise noted, the reactions were carried out with *N*-(quinolin-8-yl)pivalamide (0.3 mmol), *N*-bromo-succinimide (NBS) or Br_2_ (0.6 mmol), a catalyst (5–20%), an additive (0.6 mmol) in water (1 mL) under air atmosphere at room temperature for 24 h. ^b^ Isolated yields. ^c^ N_2_ atmosphere. ^d^ CH_3_(CH_2_)_5_COOH (0.3 mmol), NaHCO_3_ (0.3 mmol).

## Supplementary Materials

The supplementary materials are available online.
