# Supplementary material for: Iron(III)-Catalyzed Highly Regioselective Halogenation of 8-Amidoquinolines in Water
Source: Molecules. 2019 Feb 1;24(3):535. doi: 10.3390/molecules24030535 (PMC6384643; doi:10.3390/molecules24030535)

Supporting Information

Iron(III)-Catalyzed Highly Regioselective Halogenation of 8-Amidoquinolines in Water

Yang Long,[†] Lei Pan[†] and Xiangge Zhou*

College of Chemistry, Sichuan University, Chengdu 610064, P. R. China.

Email: zhouxiangge@scu.edu.cn, Tel: +86-28-85412904.

Table of Contents

1. X-ray crystallographic data of compound 2l 2
2. GC-MS analysis on the crude product 3
3. EPR experiment 5
4. ^{1}H and ^{13}C NMR spectra for the products 6

1. X-ray Crystallographic Data of Compound 2l

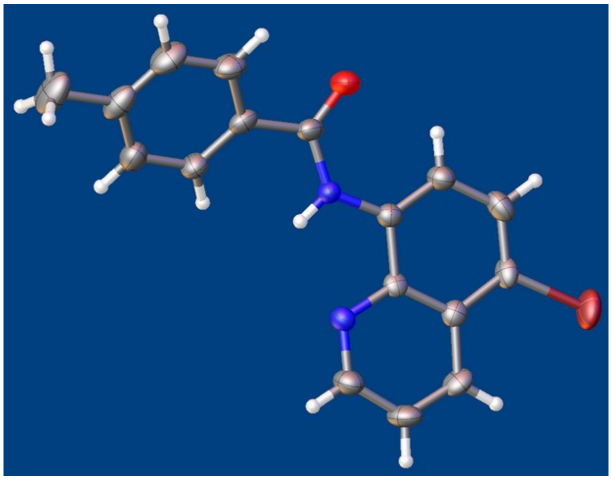

Table S1 Crystal data and structure refinement for *N*-(5-bromoquinolin-8-yl)-4-methylbenzamide (CCDC 1480727).

Identification code	**2l**
Empirical formula	$C_{17}H_{13}BrN_2O$
Formula weight	341.20
Temperature/K	294.4(2)
Crystal system	triclinic
Space group	P-1
a/Å	7.0587(5)
b/Å	8.5688(7)
c/Å	12.3472(10)
α/°	81.742(7)
β/°	80.313(7)
γ/°	81.413(6)
Volume/Å^3	722.51(10)
Z	2
ρcalcg/cm^3	1.568
μ/mm^{-1}	3.872
F(000)	344.0
Crystal size/mm^3	0.8 × 0.7 × 0.4
Radiation	CuKα (λ = 1.54184)
2Θ range for data collection/°	10.52 to 134.136
Index ranges	$-8 \le h \le 7$, $-10 \le k \le 9$, $-14 \le l \le 14$
Reflections collected	7350
Independent reflections	2585 [R_{int} = 0.0602, R_{sigma} = 0.0490]
Data/restraints/parameters	2585/0/191
Goodness-of-fit on F^2	1.127
Final R indexes [I>=2σ (I)]	R_1 = 0.0988, wR_2 = 0.2289
Final R indexes [all data]	R_1 = 0.1023, wR_2 = 0.2368
Largest diff. peak/hole / e Å^{-3}	0.77/-3.13

CCDC 1480727 contains the supplementary crystallographic data for this paper. These data can be obtained free of charge via http://www.ccdc.cam.ac.uk/conts/retrieving.html (or from the CCDC, 12 Union Road, Cambridge CB2 1EZ, UK; Fax: +44 1223 336033; E-mail: deposit@ccdc.cam.ac.uk)

2. GC-MS analysis on the crude product

The GC-MS analysis on the crude product of 2c was done, and the detailed GC analysis and MS spectrum were as follows.

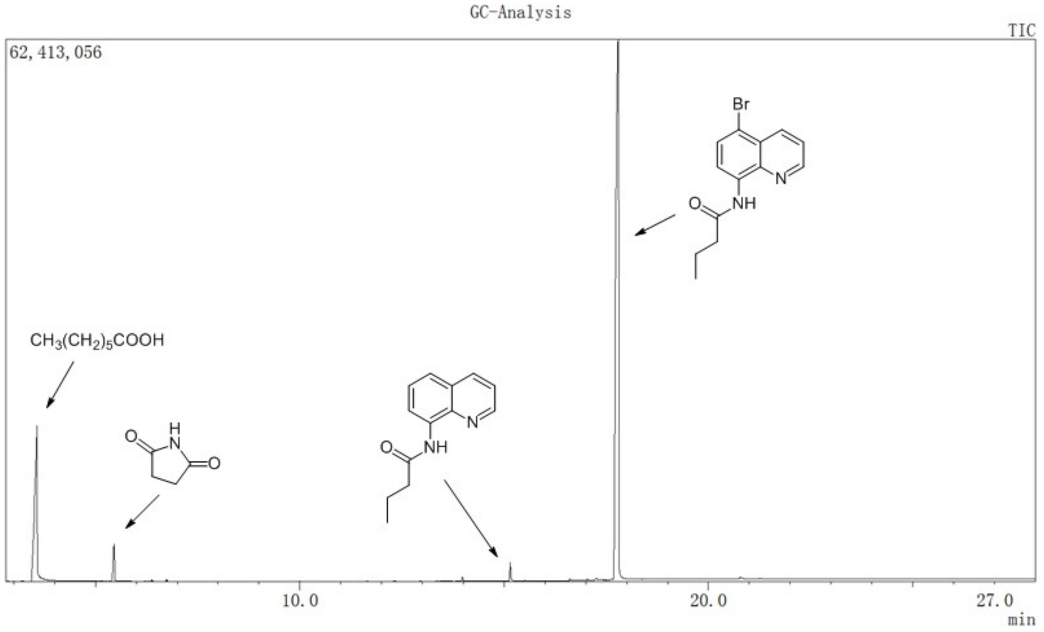

Figure S1. GC Analysis on the crude product

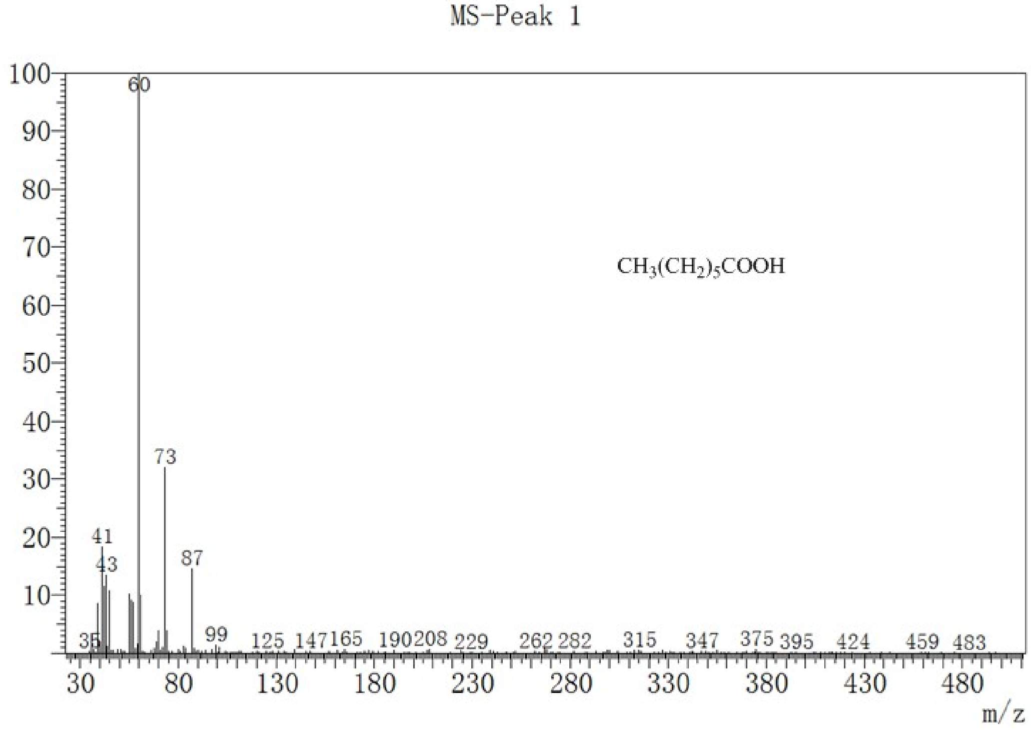

Figure S2. MS spectrum of peak 1

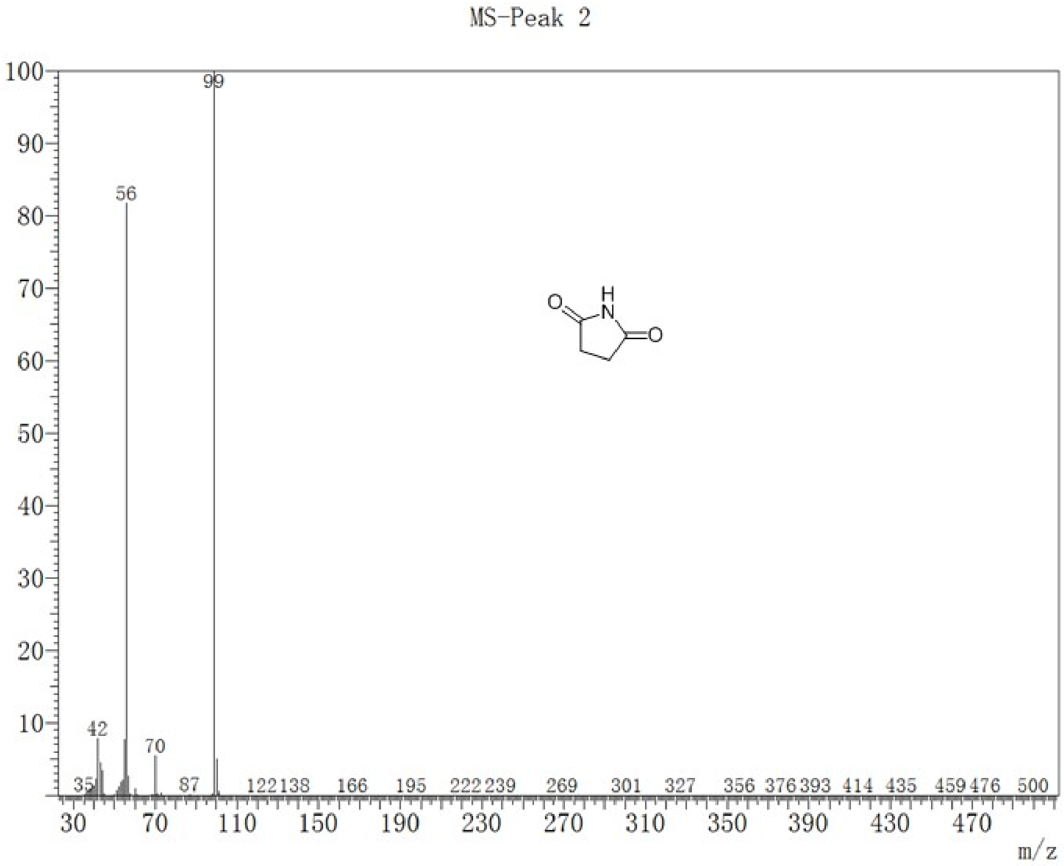

Figure S3. MS Spectrum of peak 2

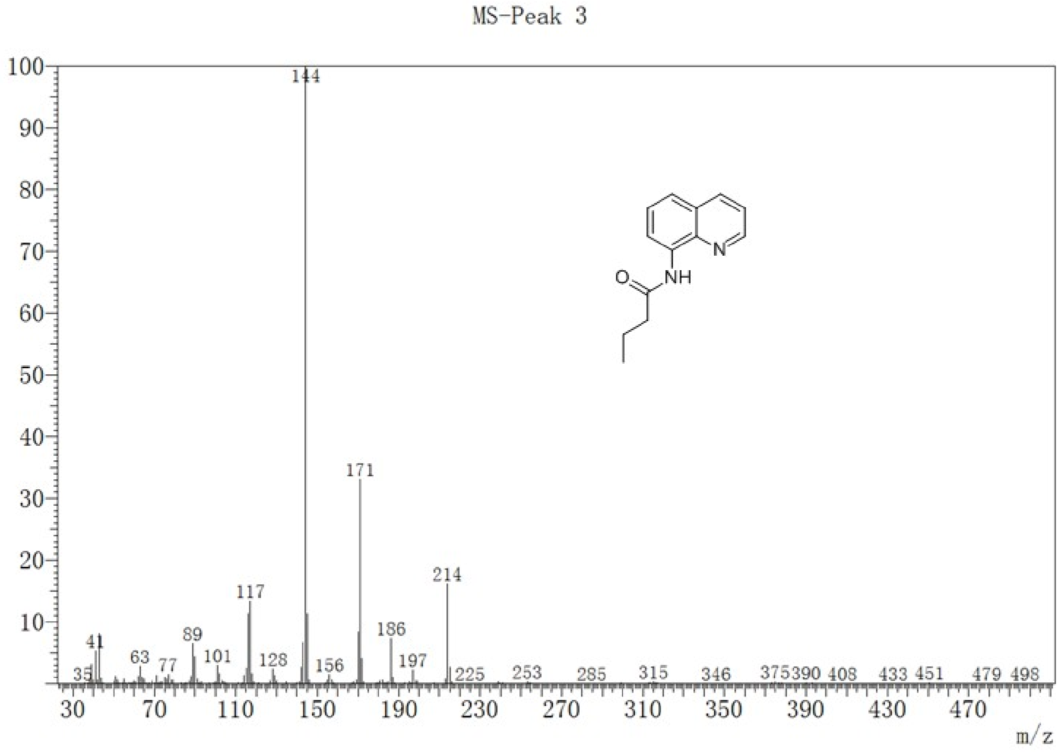

Figure S4. MS spectrum of peak 3

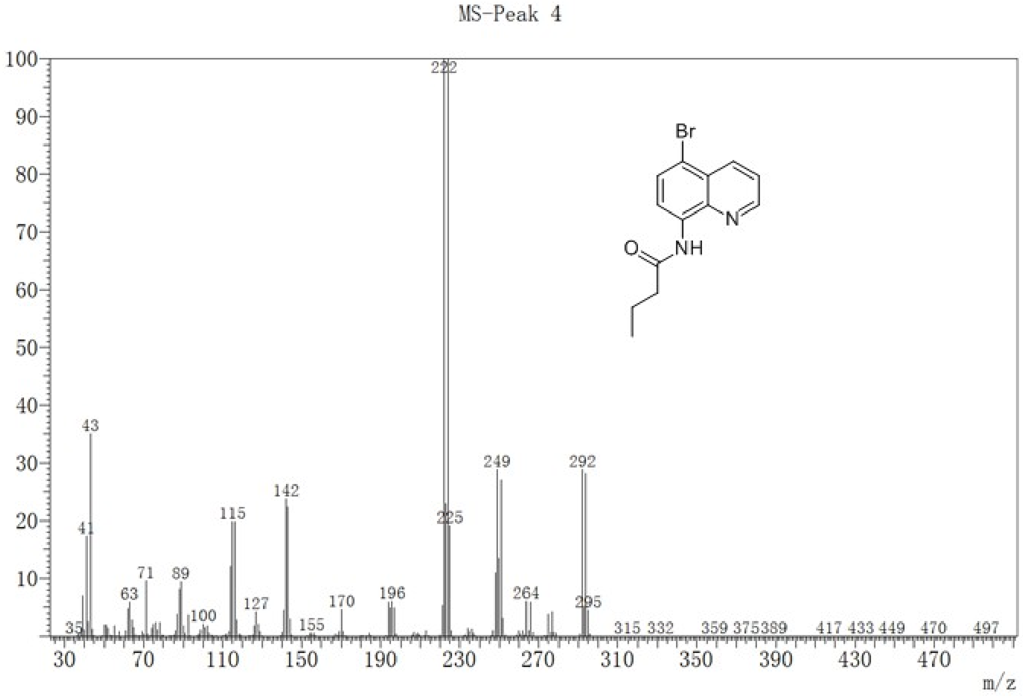

Figure S5. MS spectrum of peak 4

3. EPR expriement

EPR spectra was recorded at room temperature on EPR spectrometer (Brucker A300) using DMPO and operated at 9.434 GHz. Typical spectrometer parameters are shown as follows, scan range: 30 G; center field set: 3358.980 G; time constant: 20.48 msec; conversion time: 40 msec; sweep time: 81.92 sec; modulation amplitude: 0.50 G; moldulation frequency: 100 kHz; receiver gain: 1.00×10^{3}; microwave power: 59.15 mW.

As shown in Figure S6, the EPR spectra corroborated with the radicial mechanism.

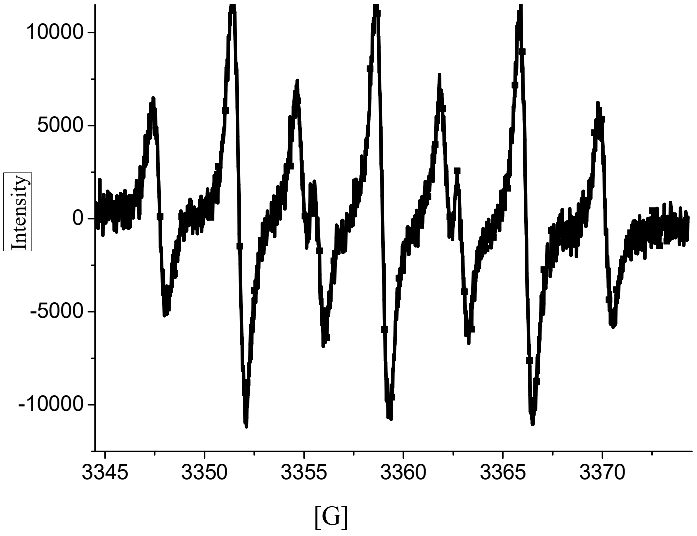

Figure S6. EPR spectrum

4. ^{1}H NMR and ^{13}C NMR spectra for the products

N-(5-bromoquinolin-8-yl)acetamide (2a)

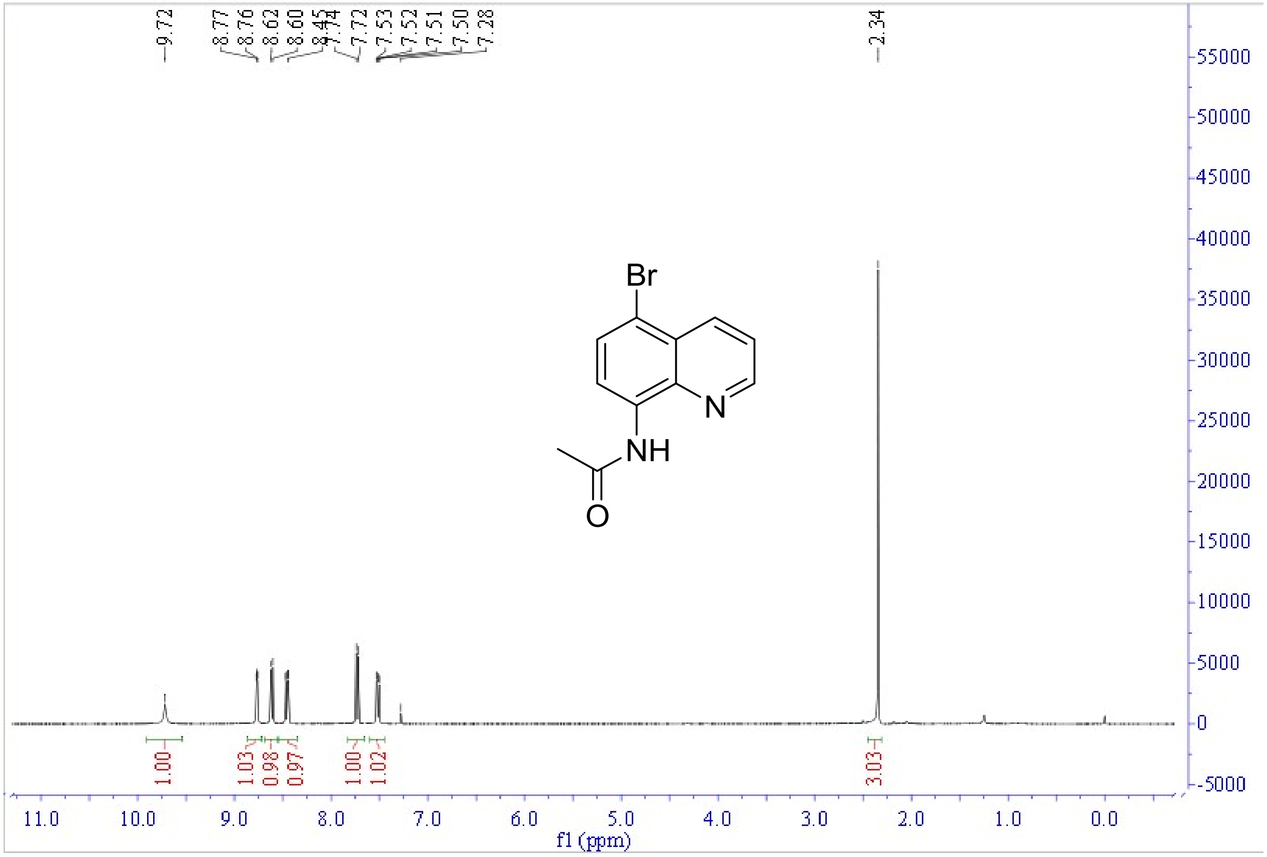

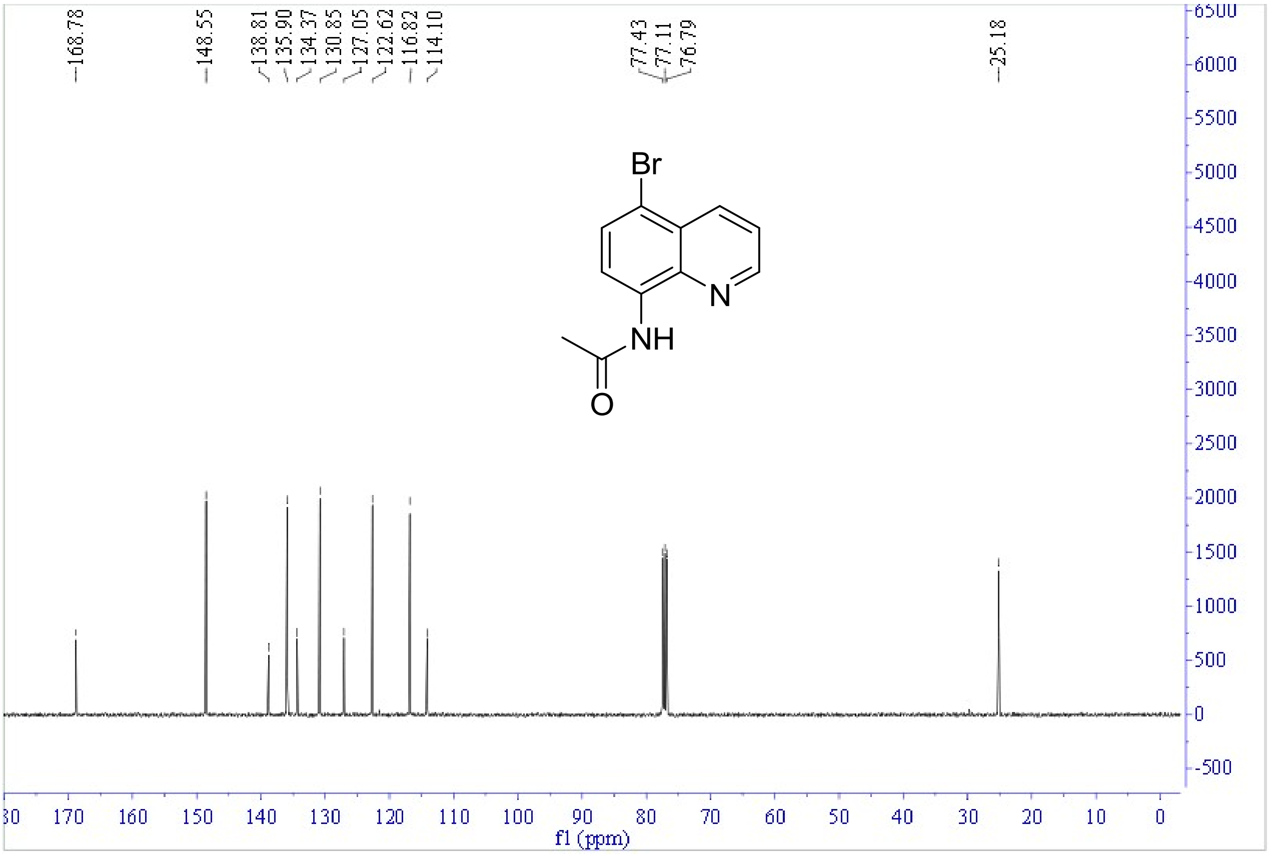

N-(5-bromoquinolin-8-yl)propionamide (2b)

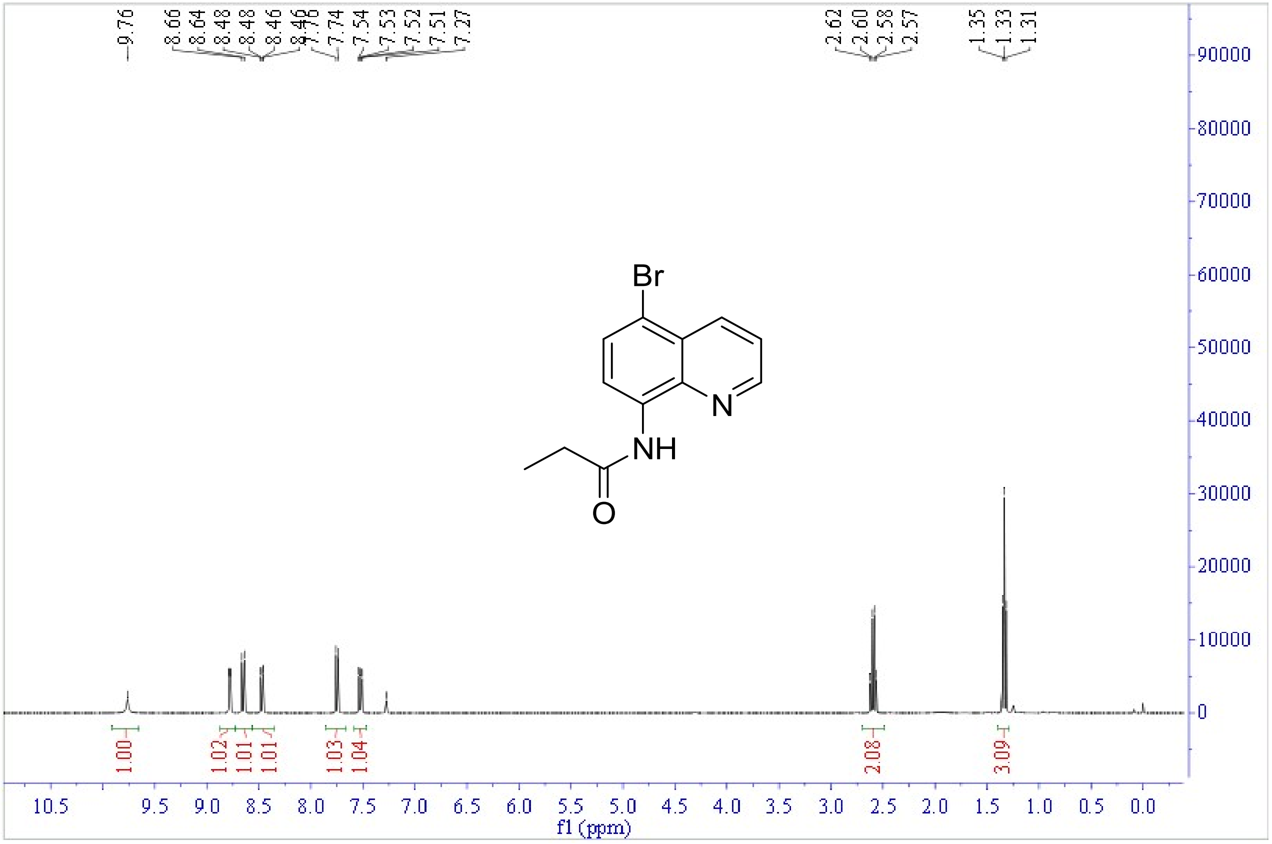

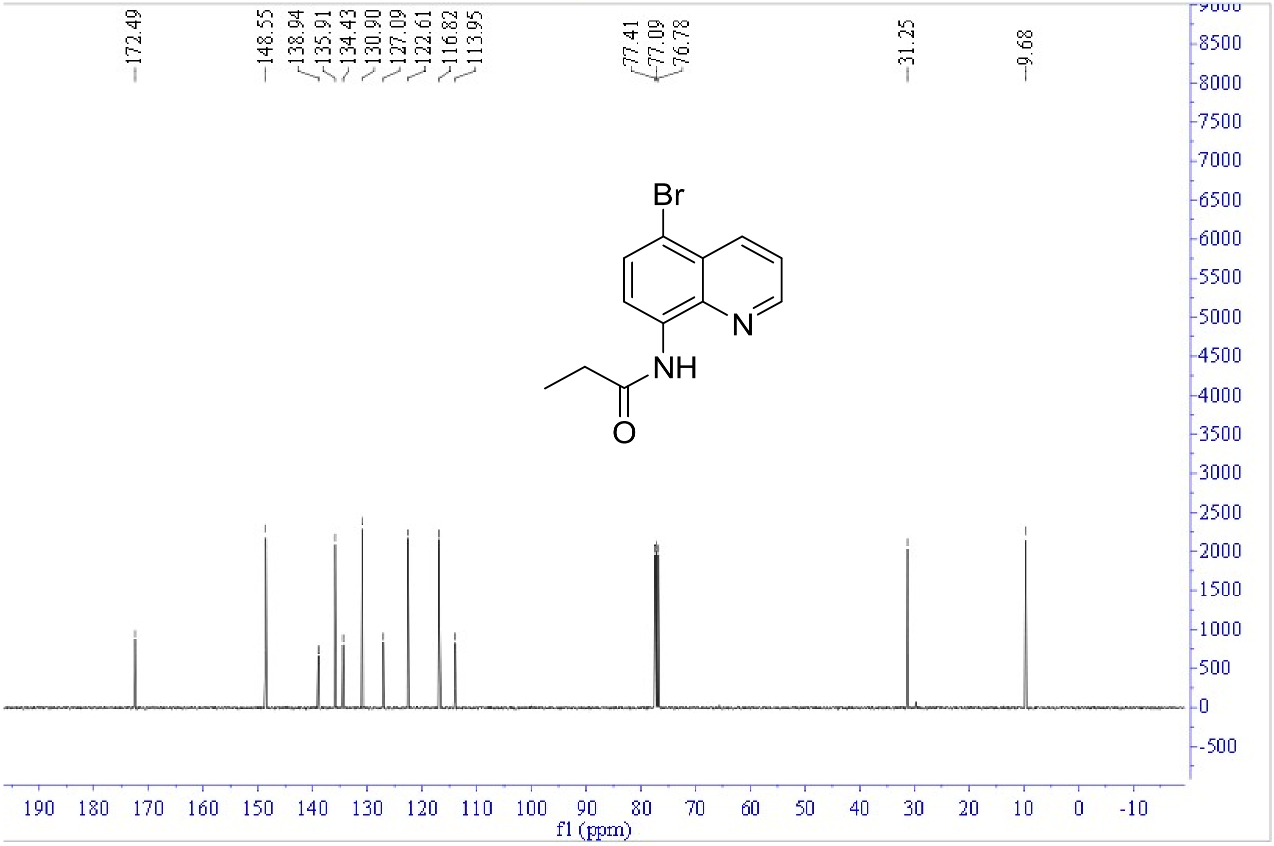

N-(5-bromoquinolin-8-yl)butyramide (2c)

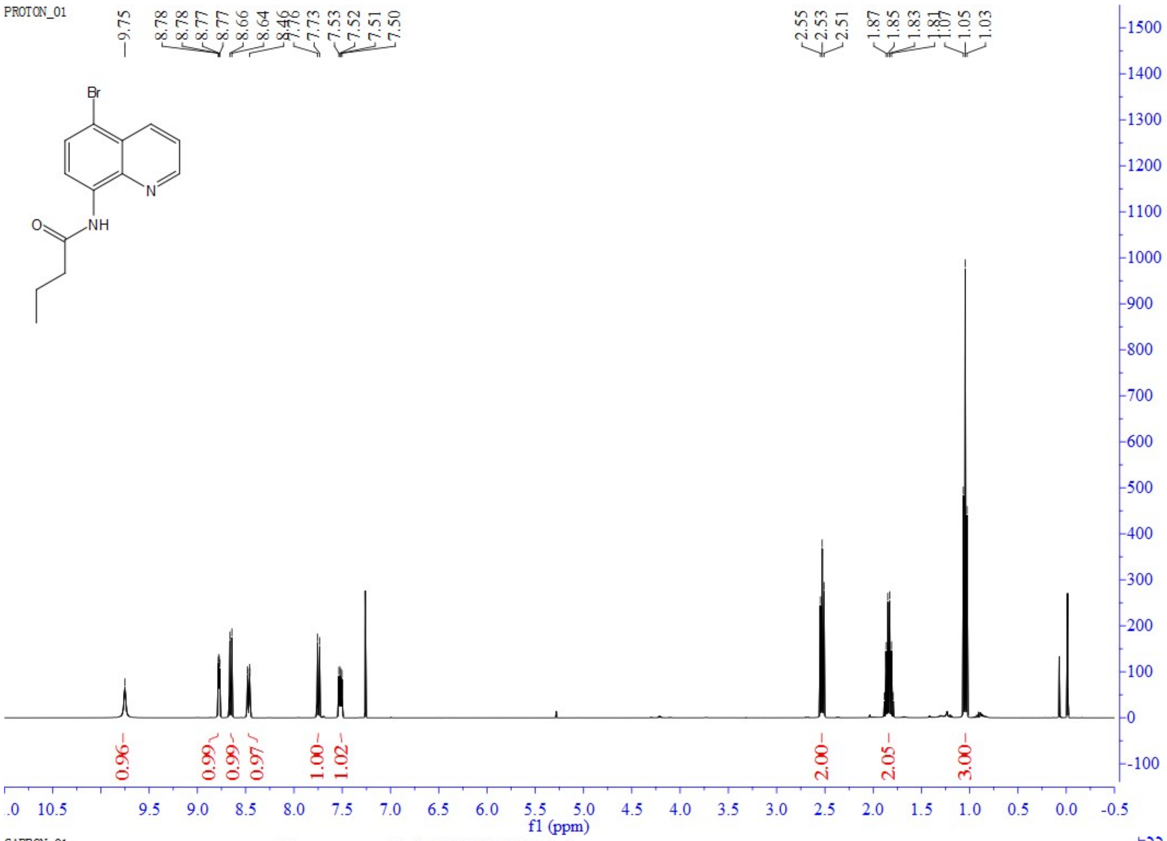

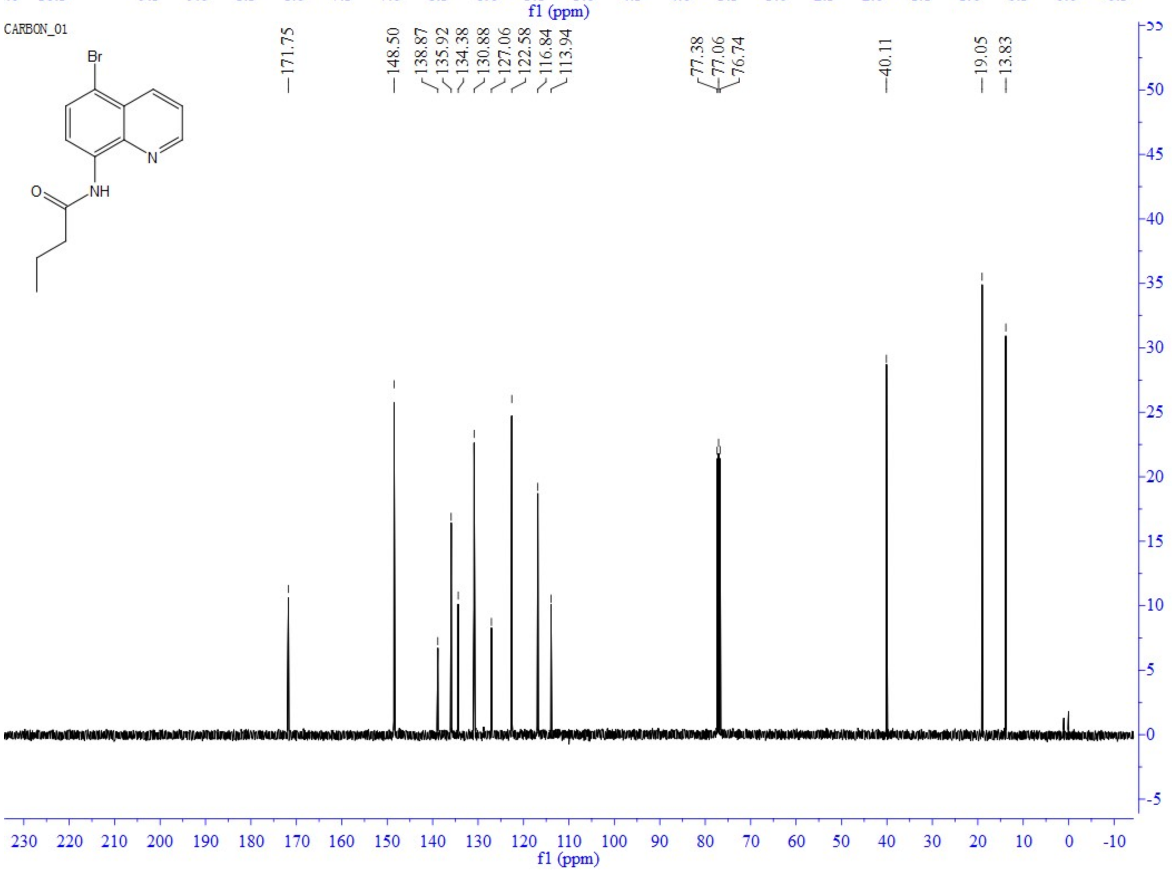

N-(5-bromoquinolin-8-yl)hexanamide (2d)

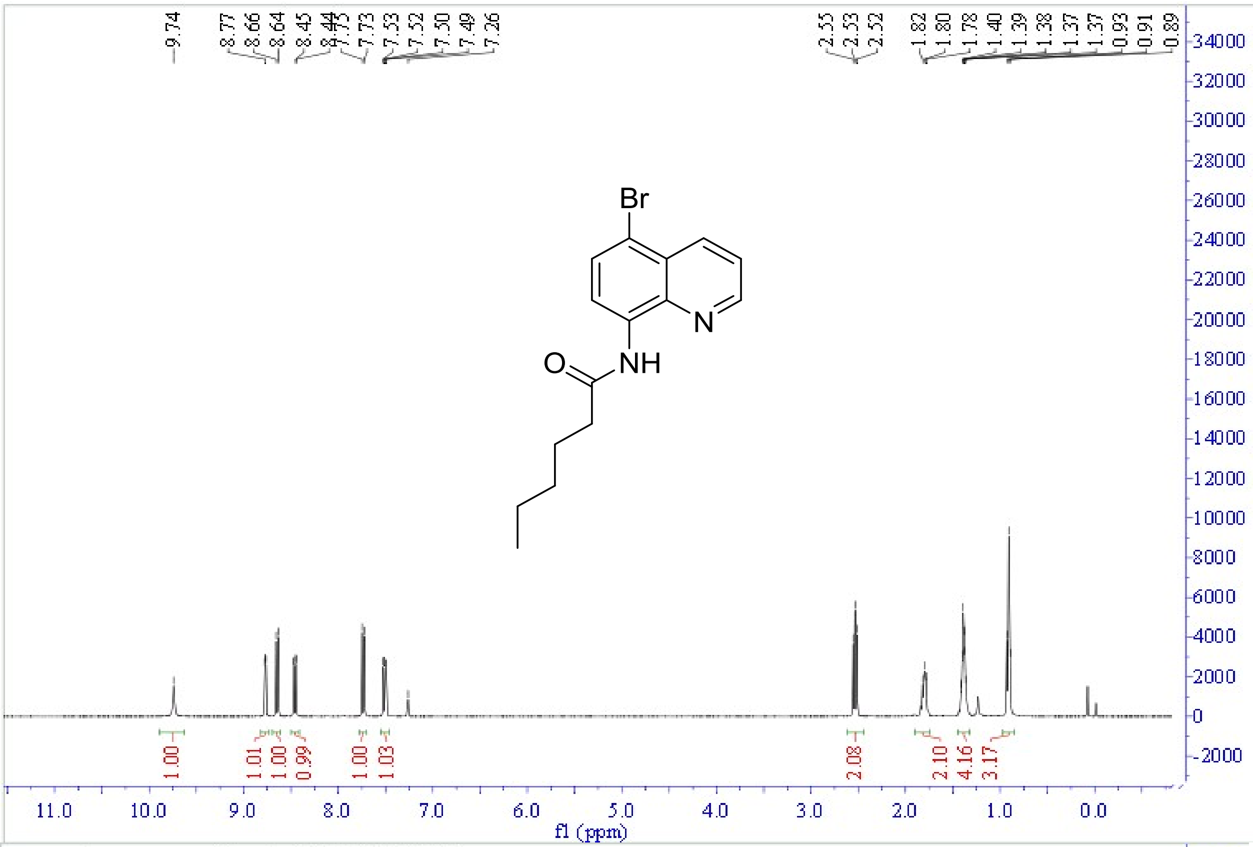

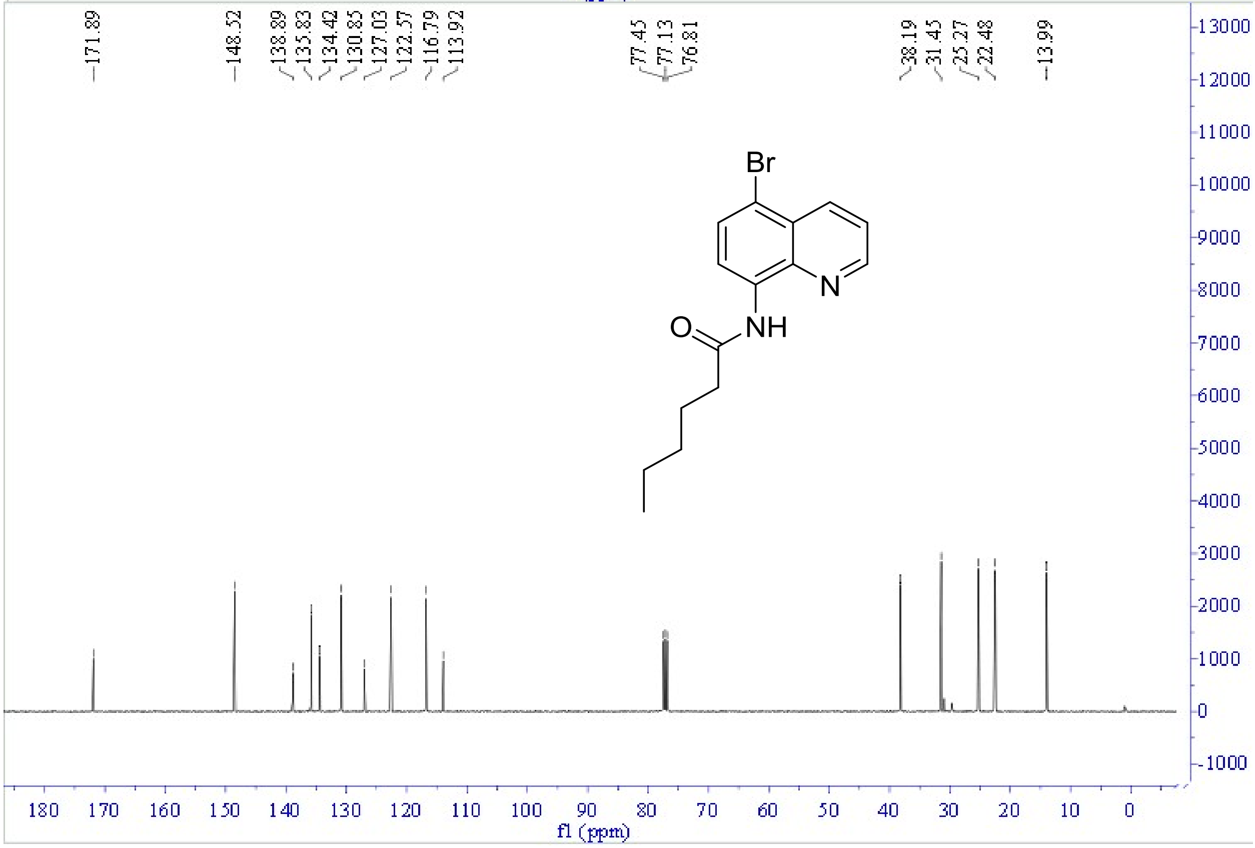

N-(5-bromoquinolin-8-yl)isobutyramide (2e)

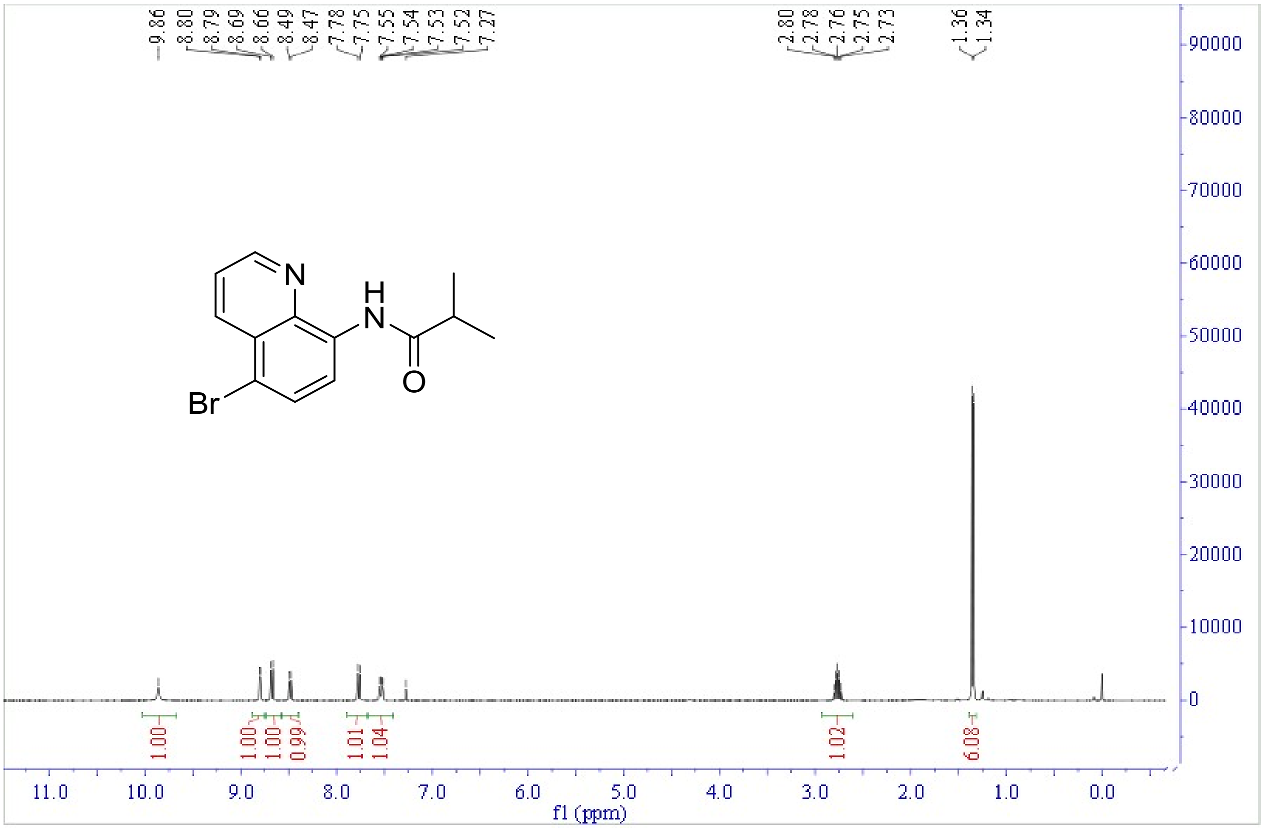

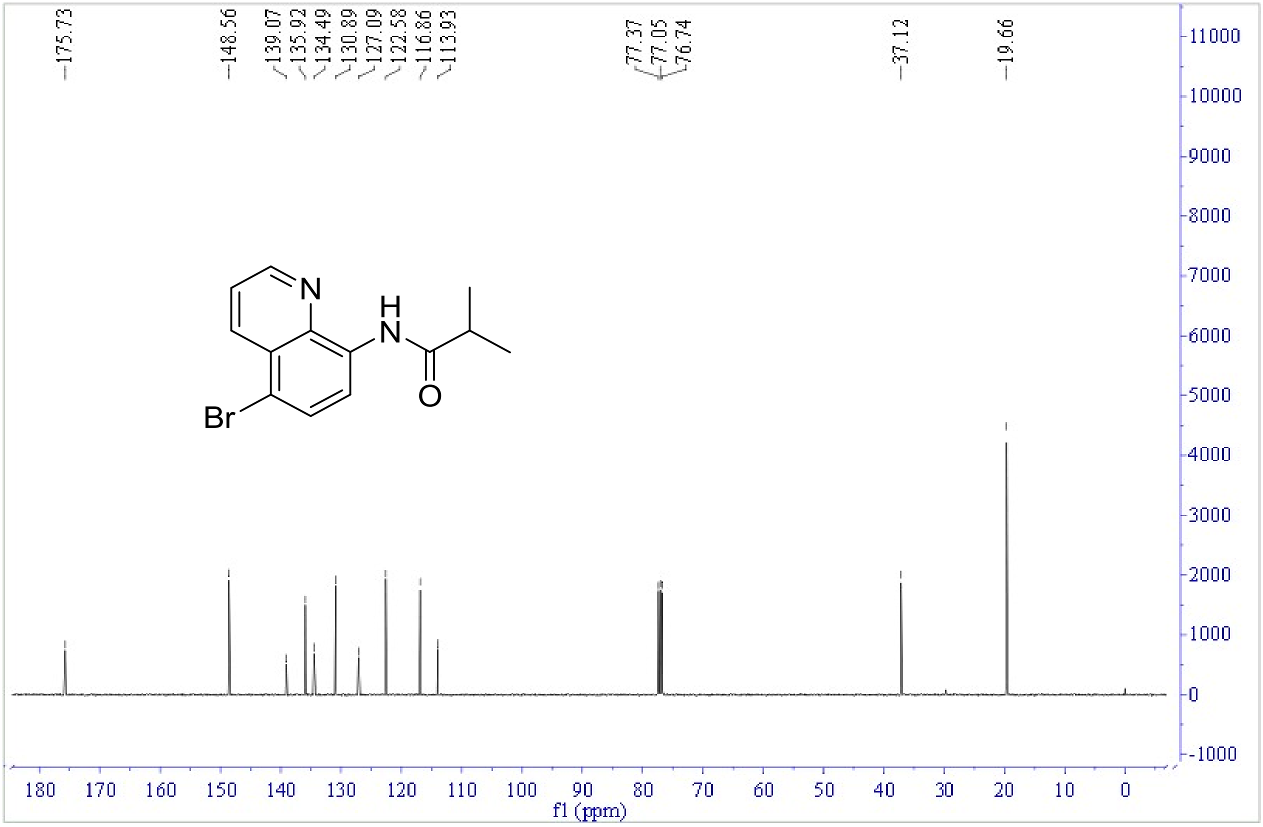

N-(5-bromoquinolin-8-yl)pivalamide (2f)

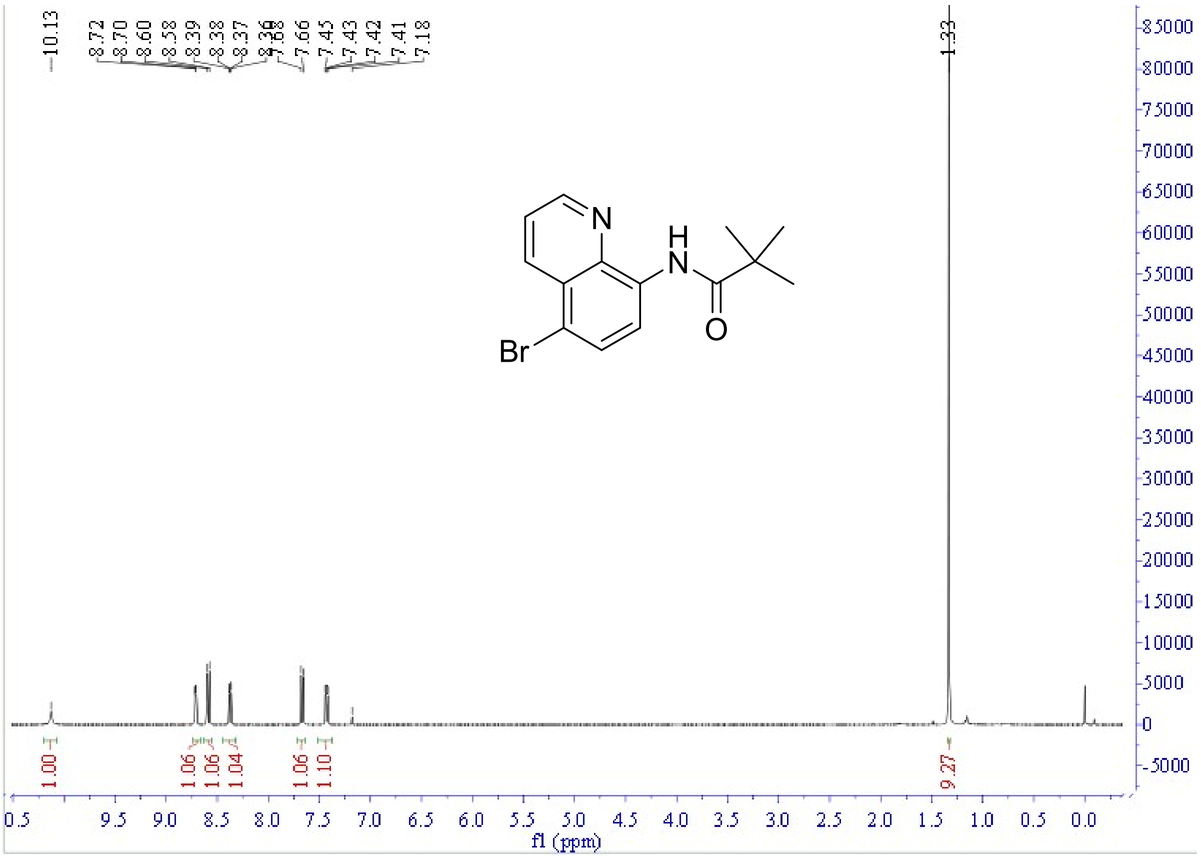

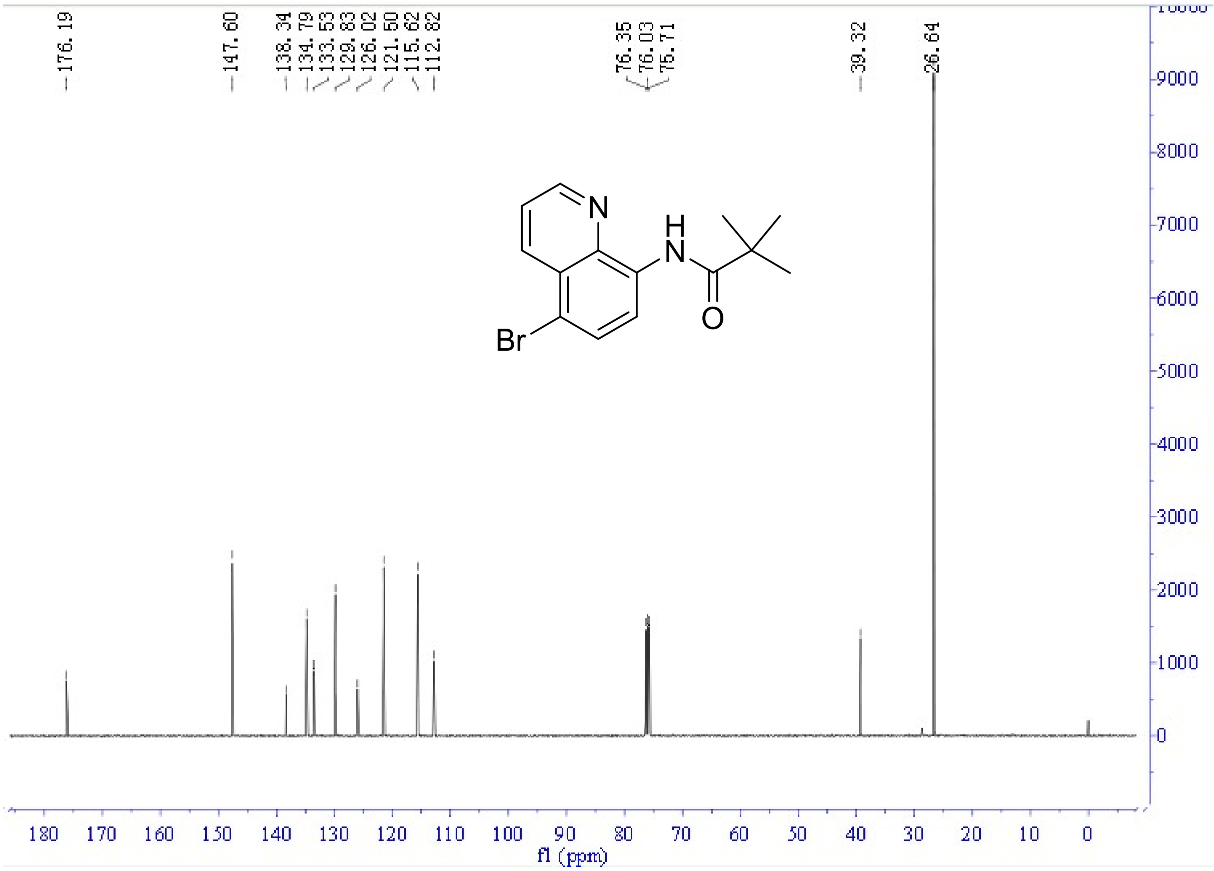

N-(5-bromoquinolin-8-yl)-2,2-dimethylbutanamide (2g)

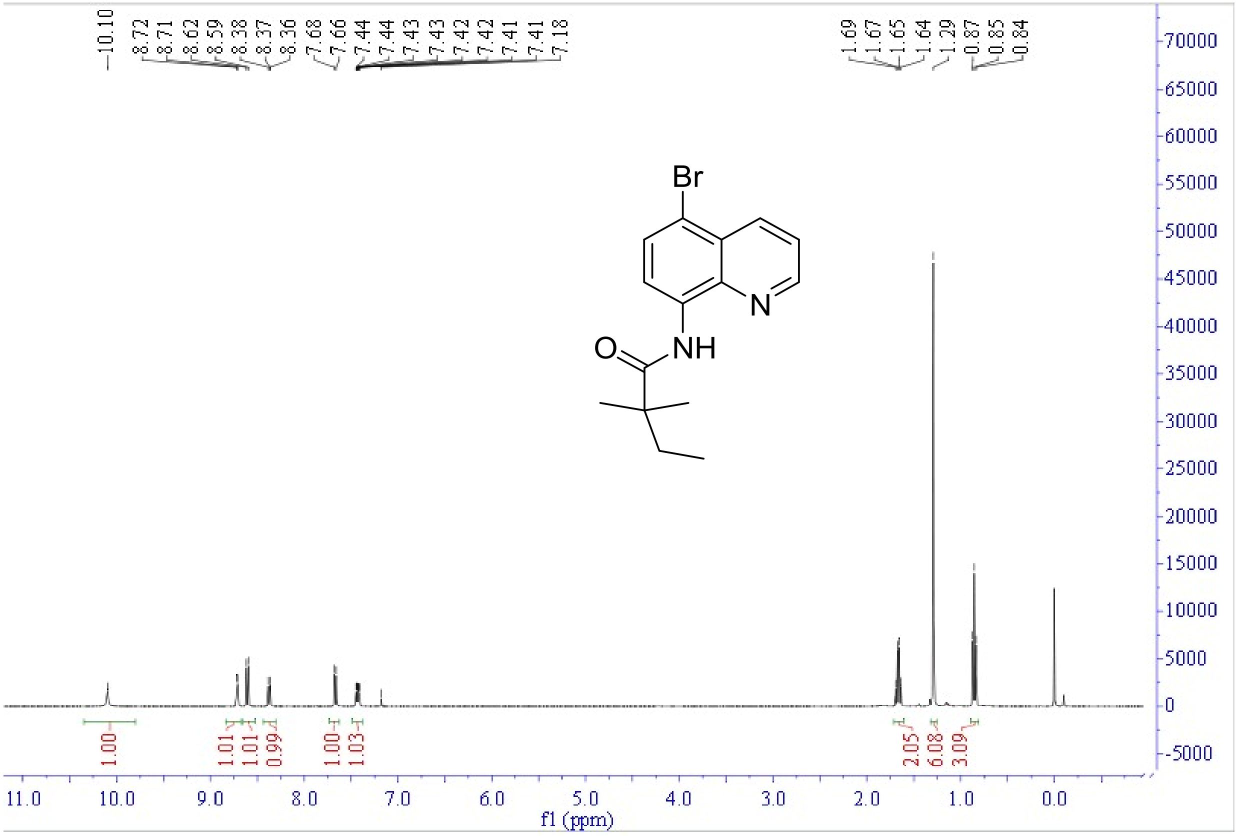

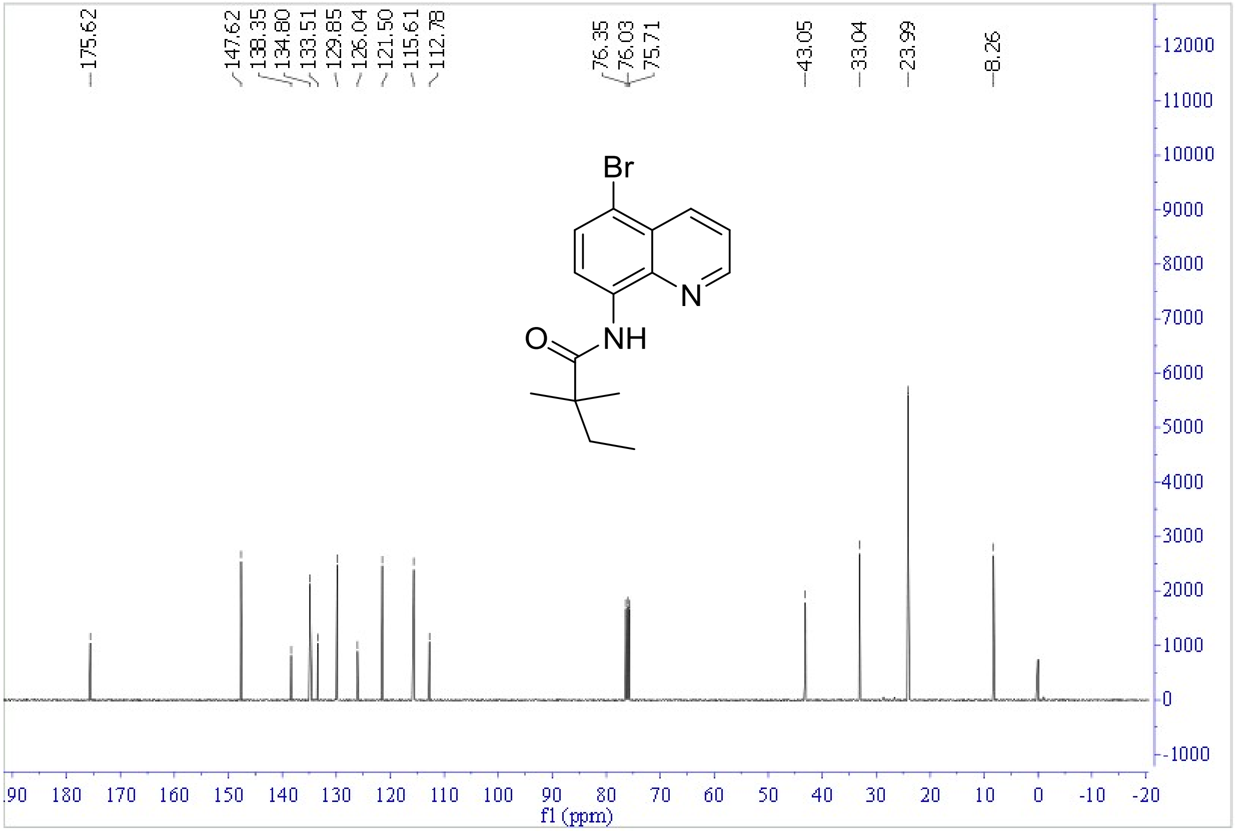

N-(5-bromoquinolin-8-yl)-3-chloro-2,2-dimethylpropanamide (2h)

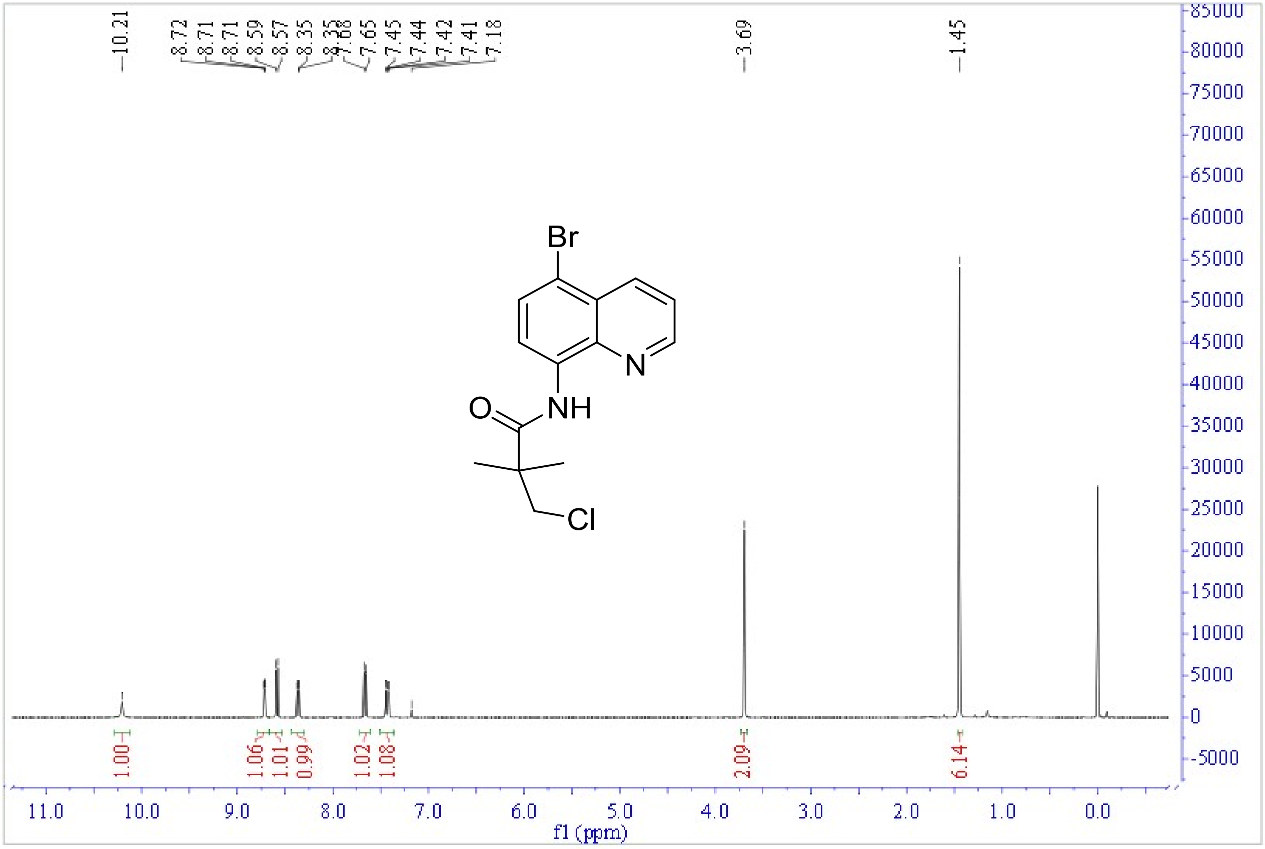

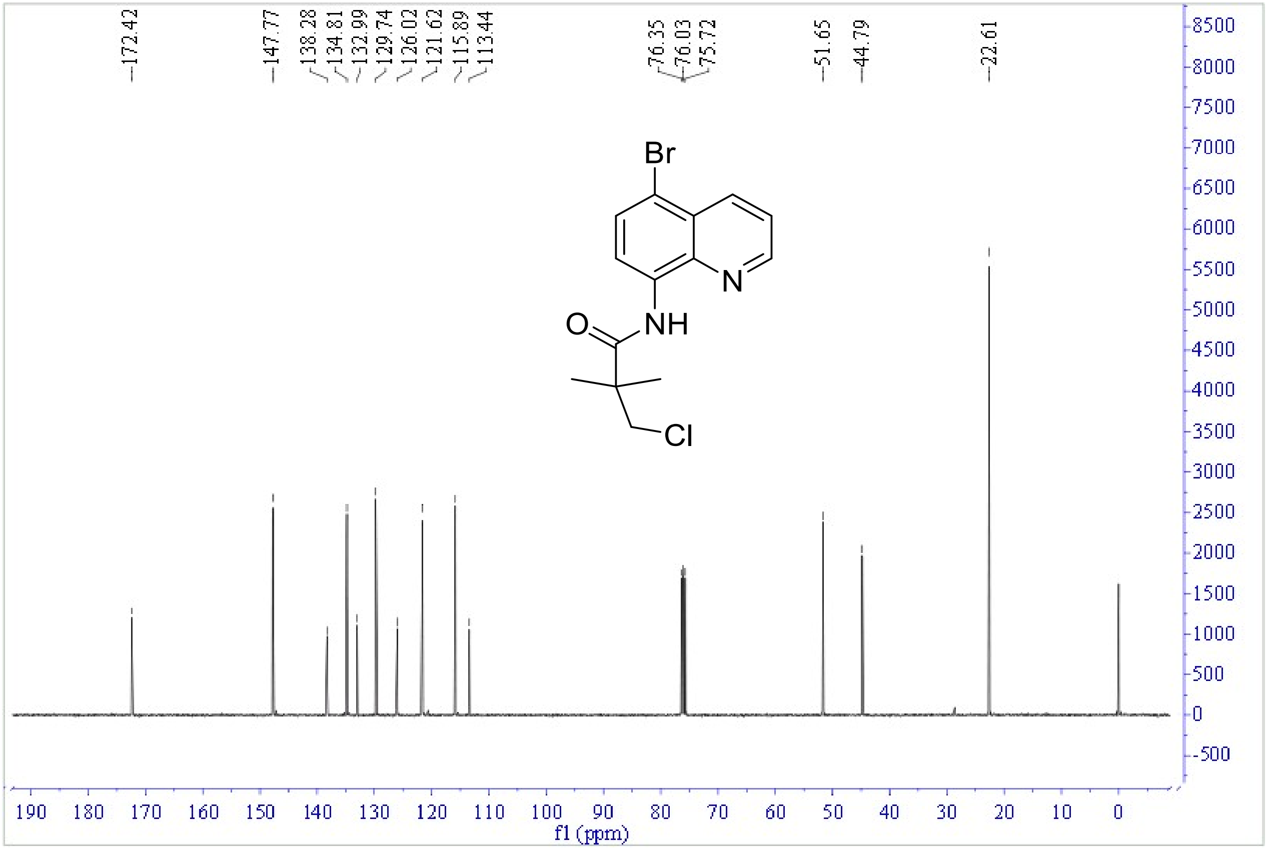

N-(5-bromoquinolin-8-yl)-3-cyclopentylpropanamide (2i)

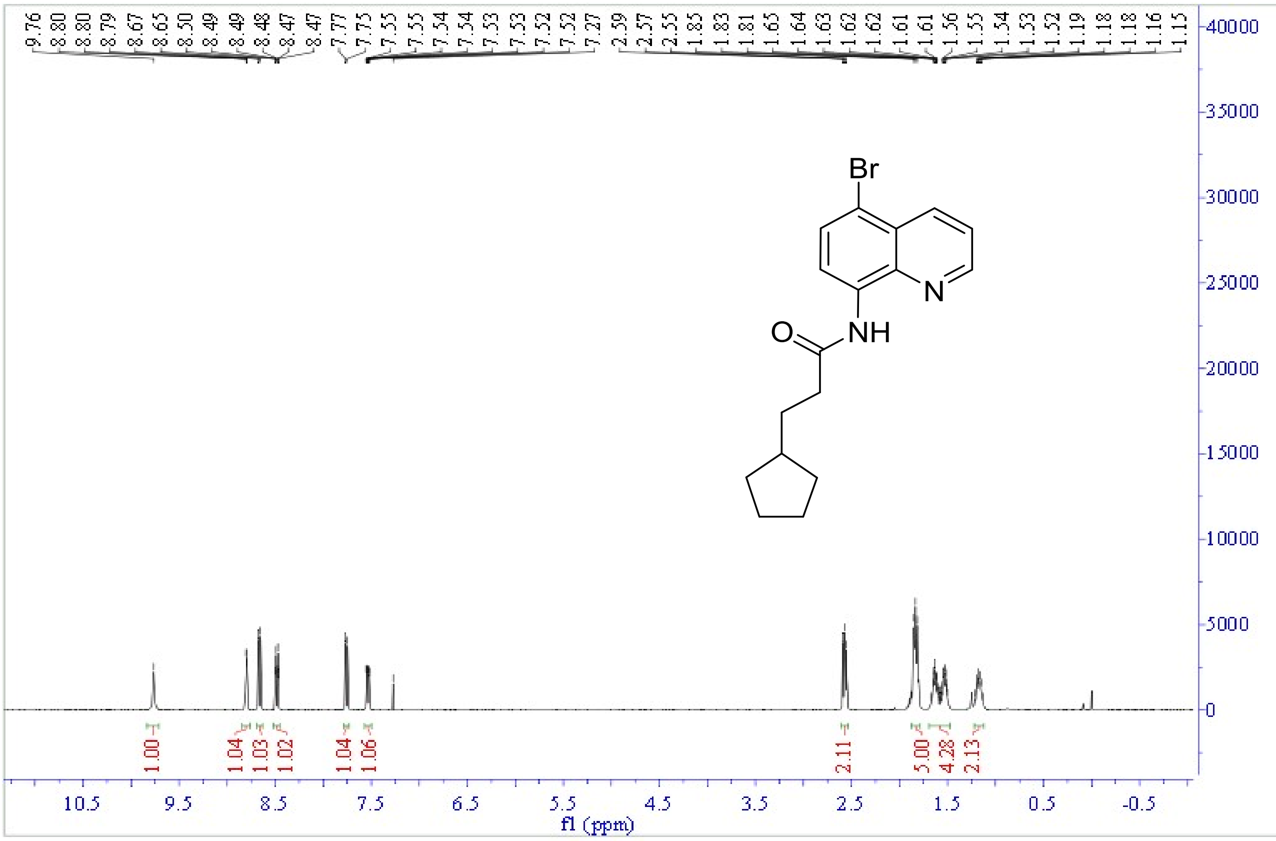

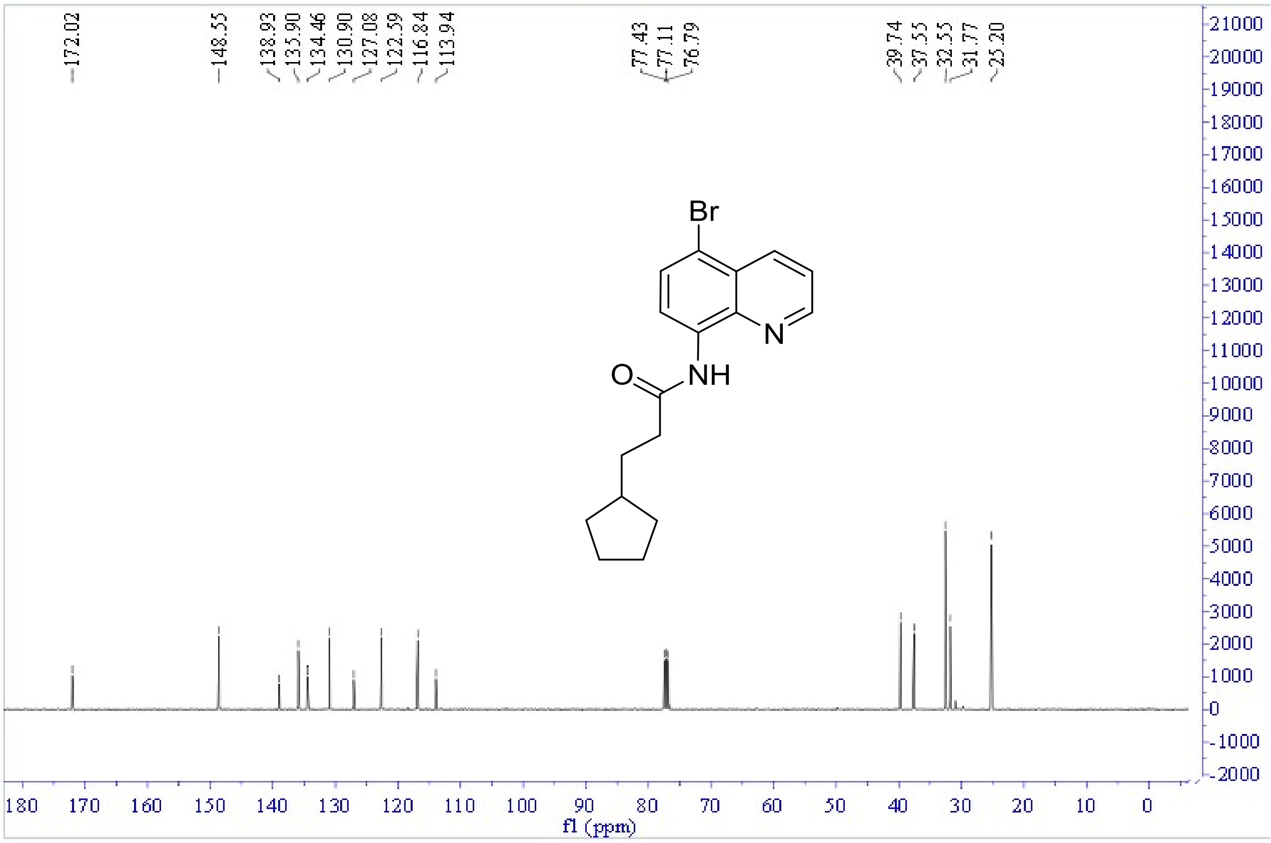

N-(5-bromoquinolin-8-yl)-4-methylpentanamide (2j)

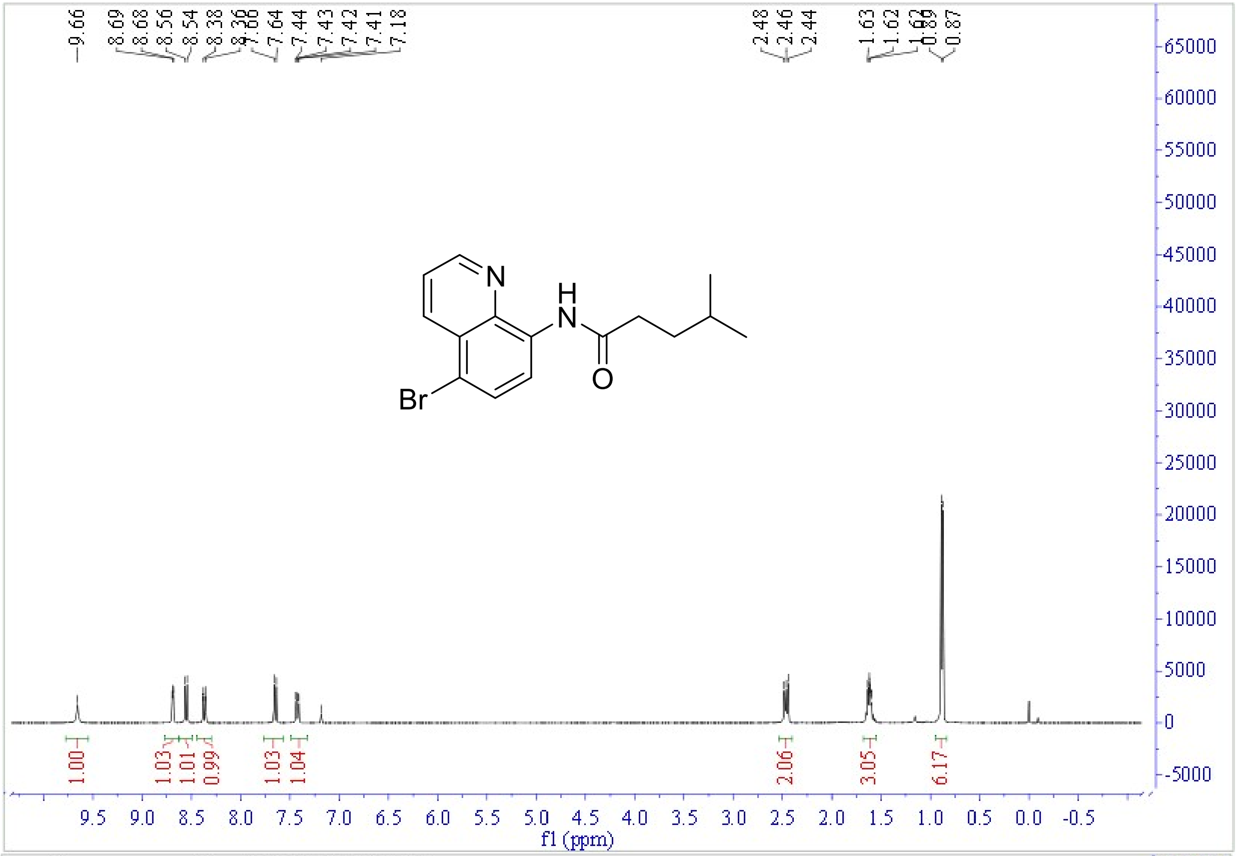

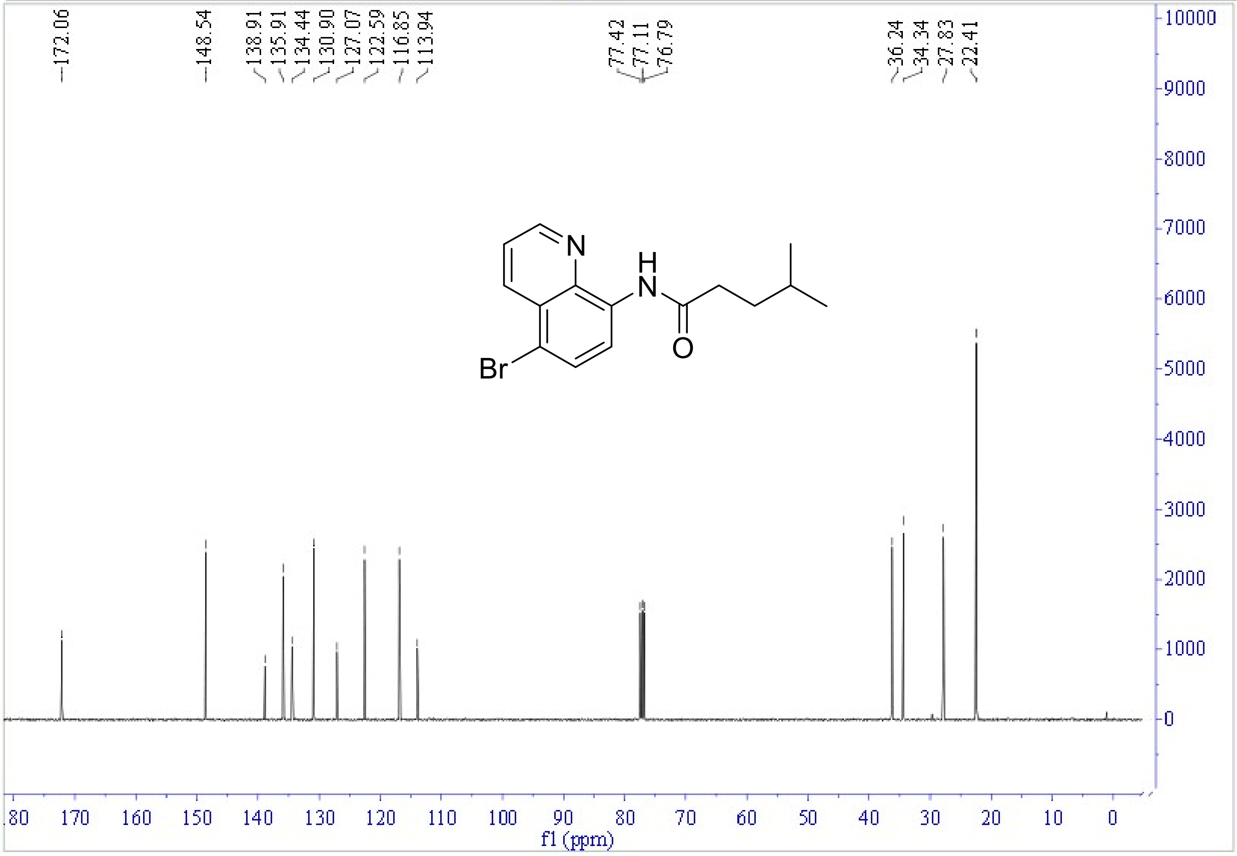

N-(5-bromoquinolin-8-yl)benzamide (2k)

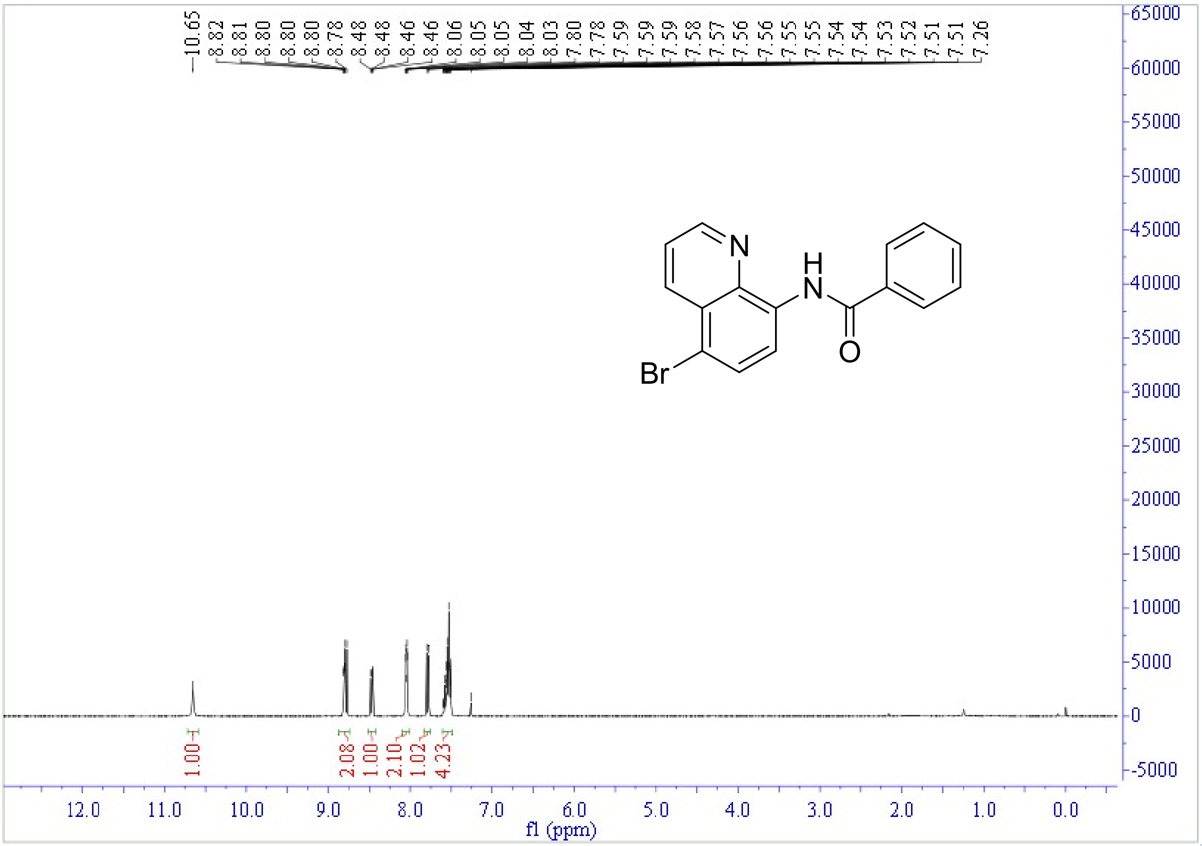

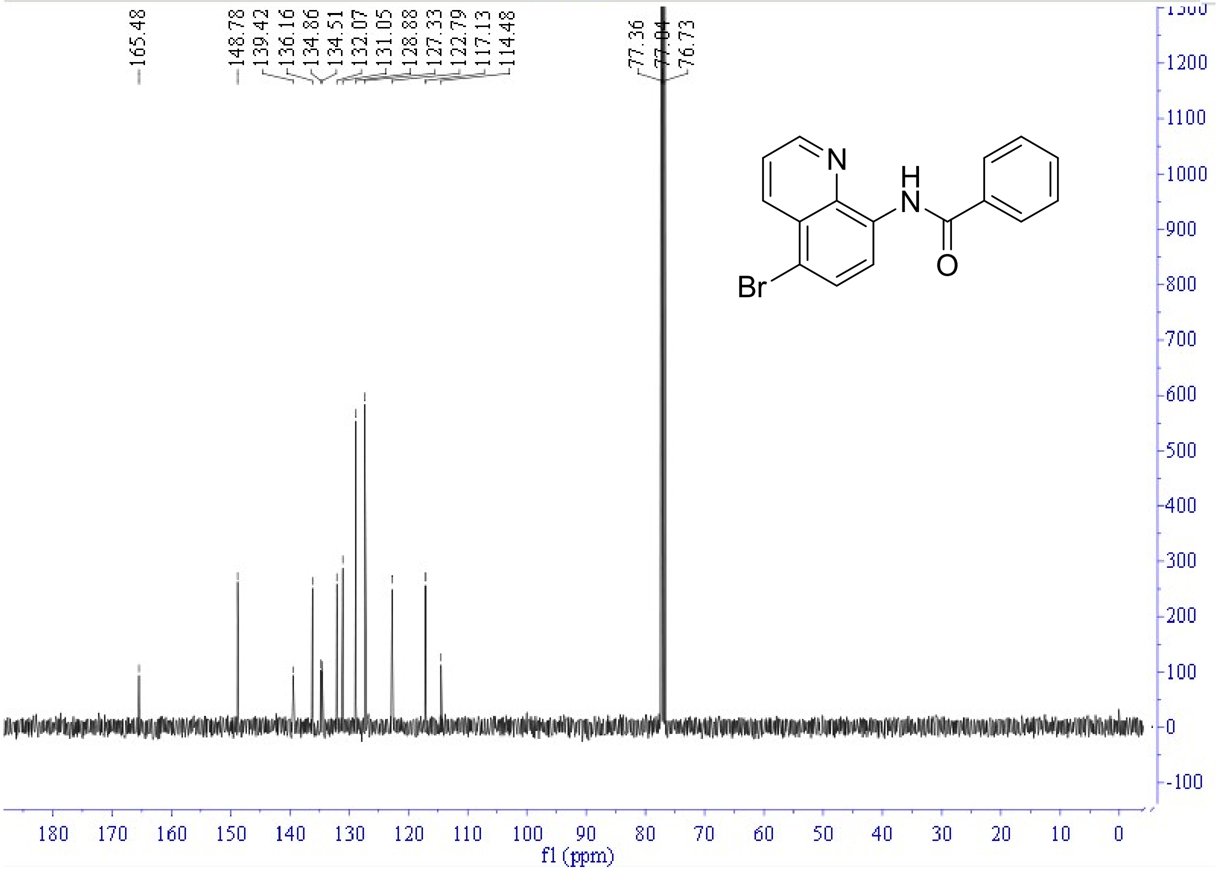

N-(5-bromoquinolin-8-yl)-4-methylbenzamide (2l)

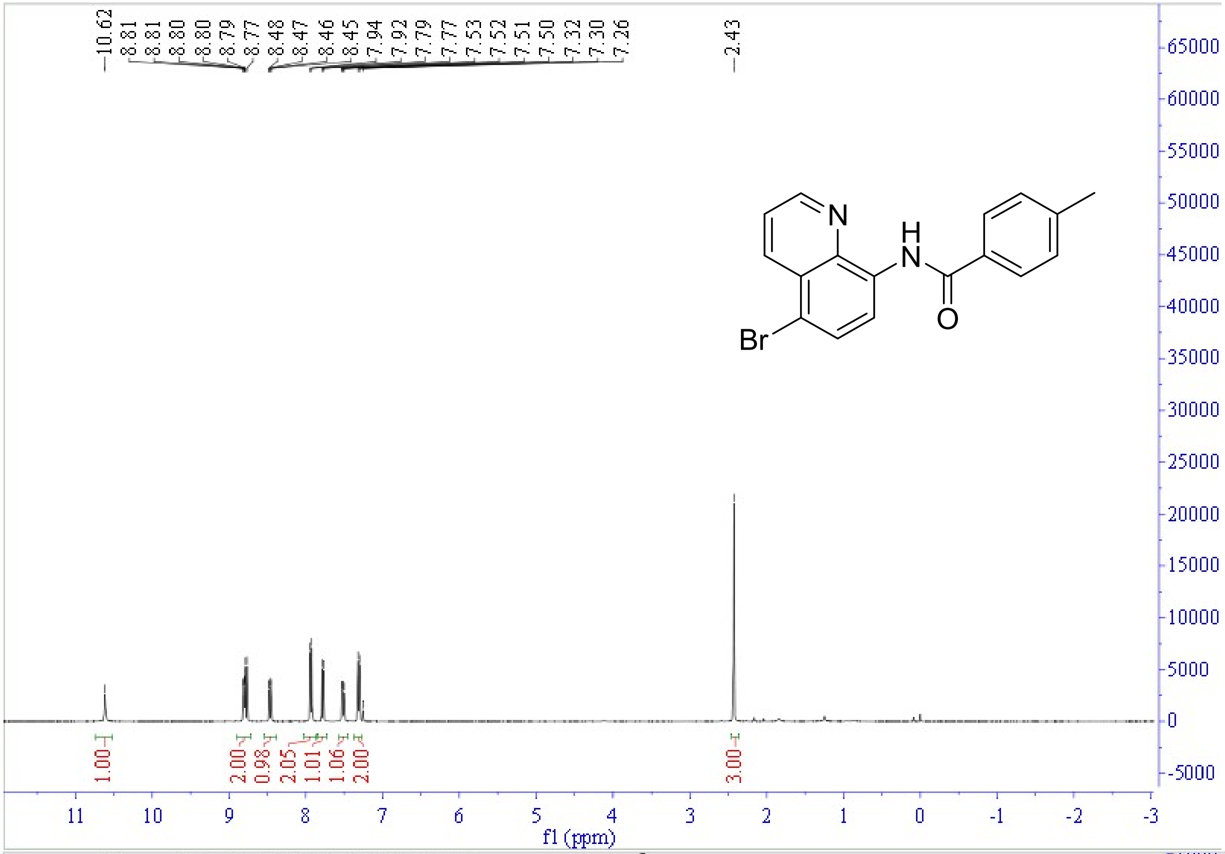

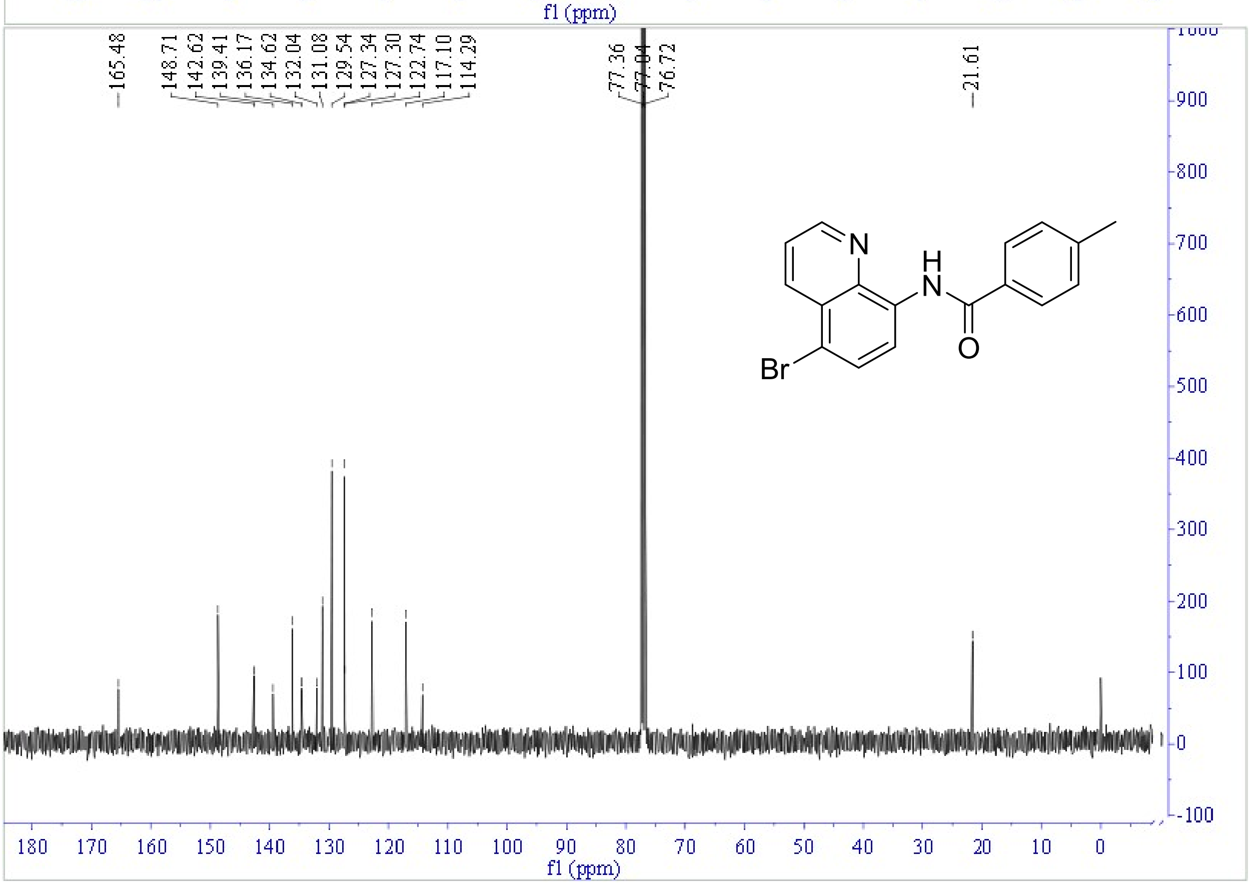

N-(5-bromoquinolin-8-yl)-4-chlorobenzamide (2m)

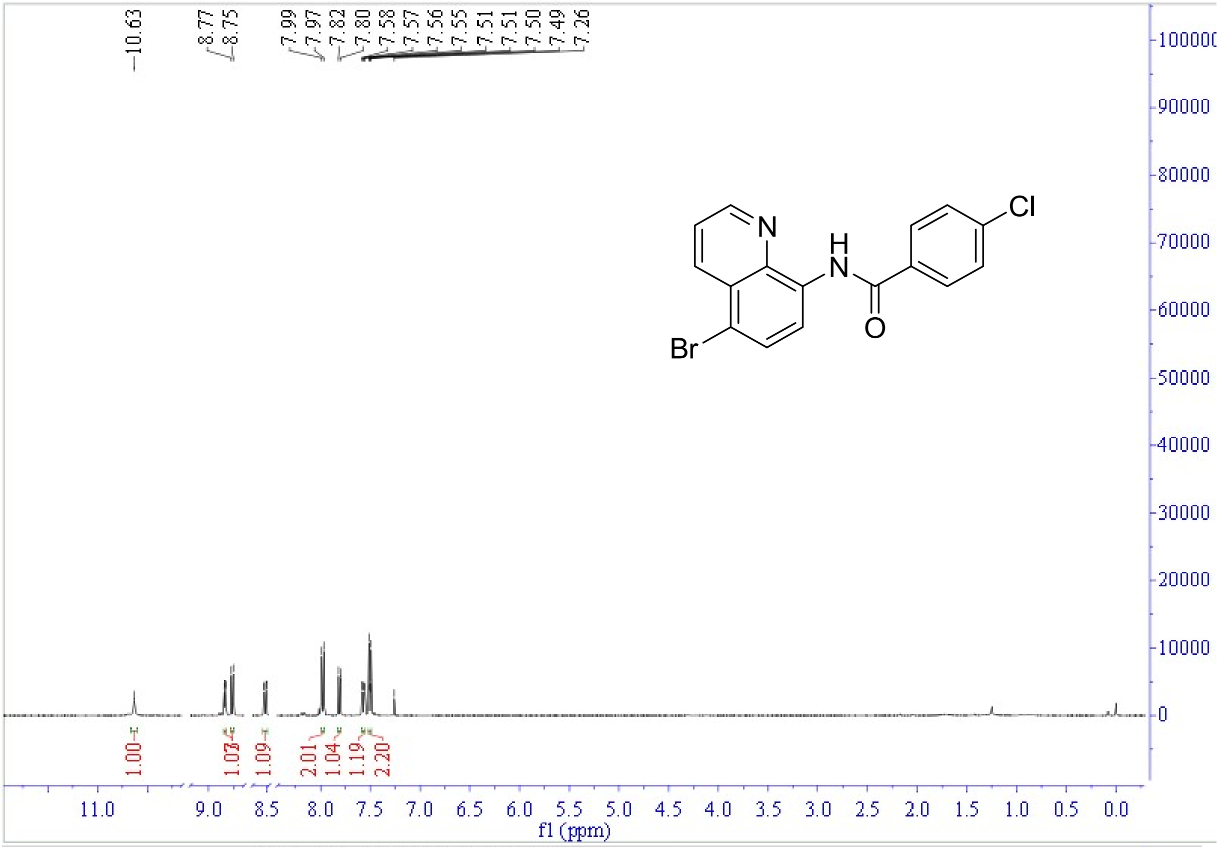

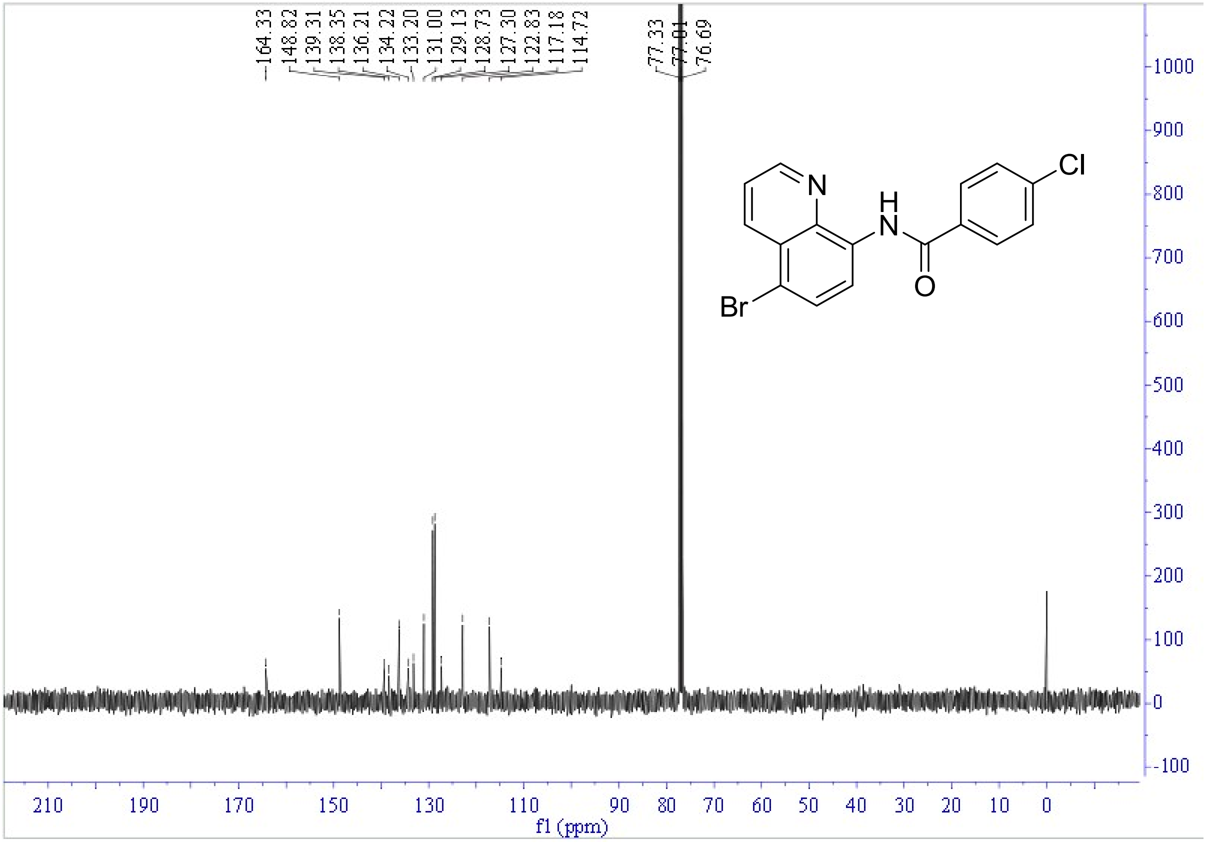

N-(5-bromoquinolin-8-yl)-4-(trifluoromethyl)benzamide (2n)

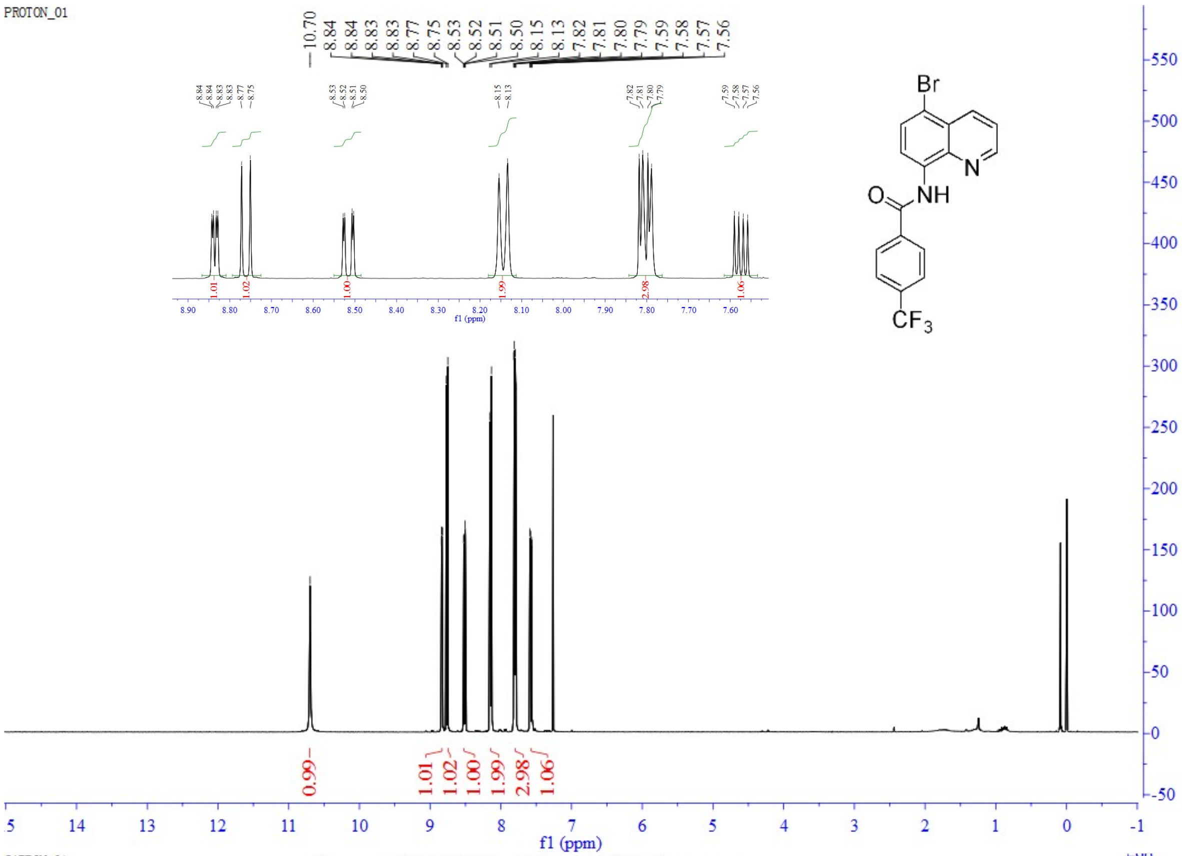

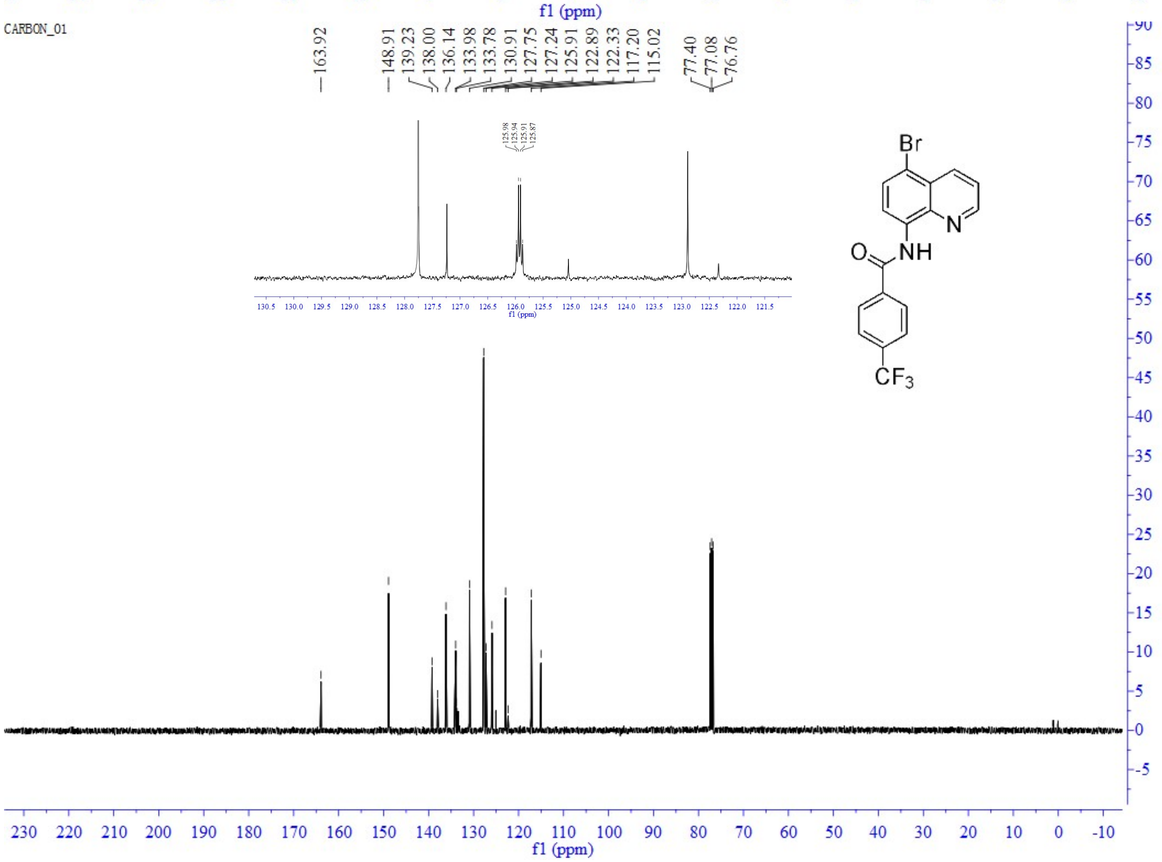

N-(5-bromoquinolin-8-yl)-3-chlorobenzamide (2o)

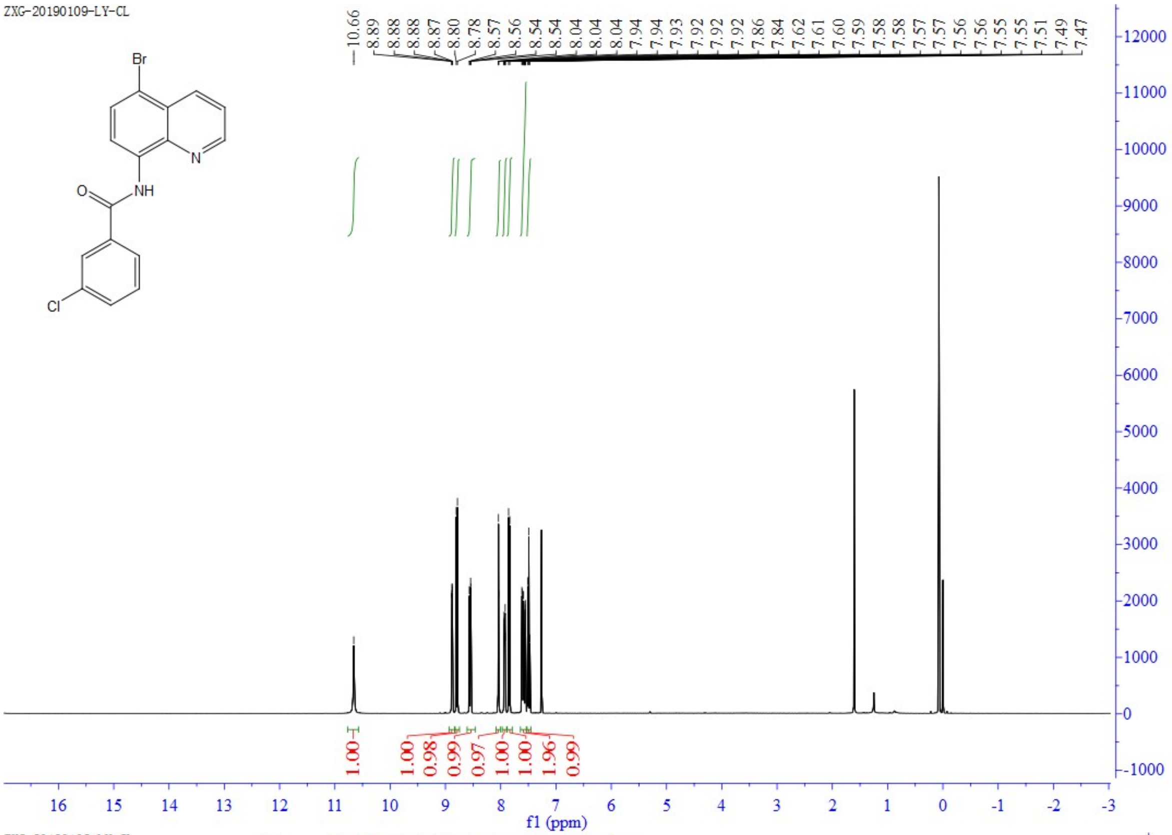

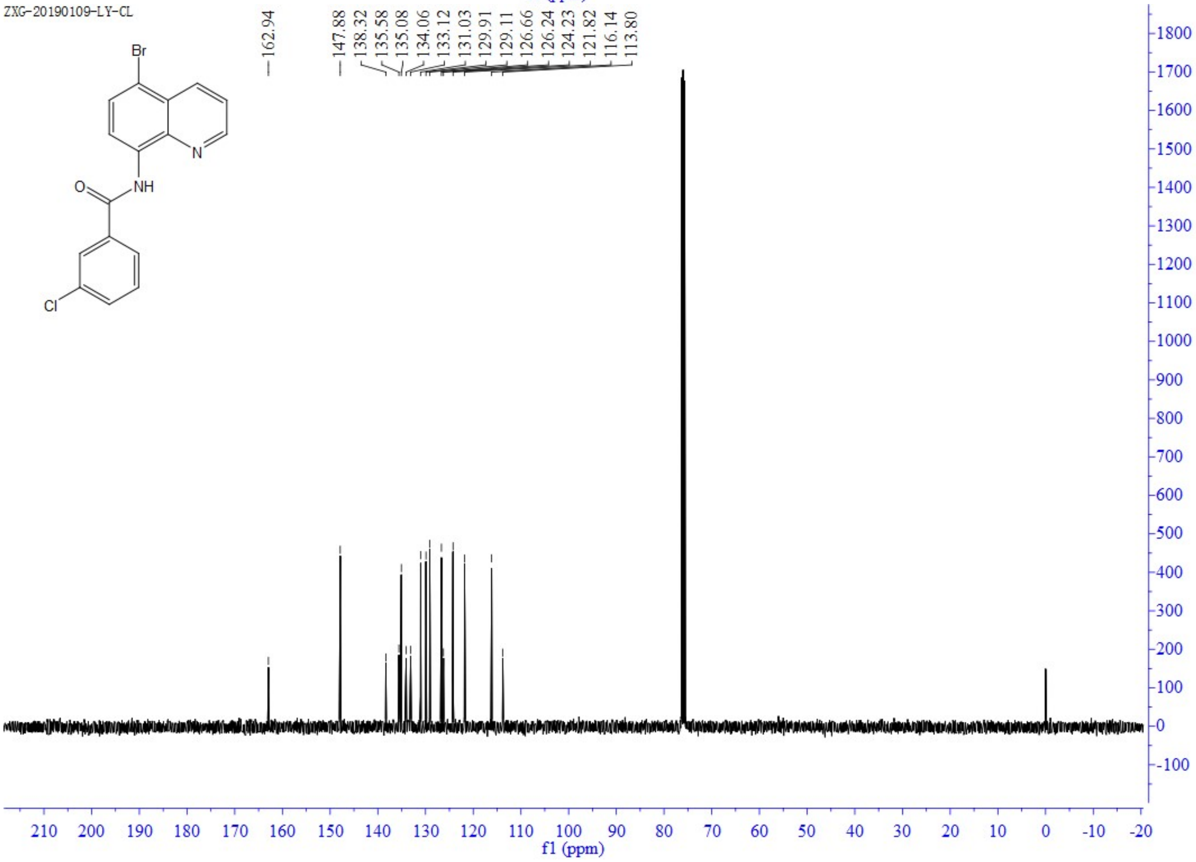

N-(5-bromoquinolin-8-yl)-3-methoxylbenzamide (2p)

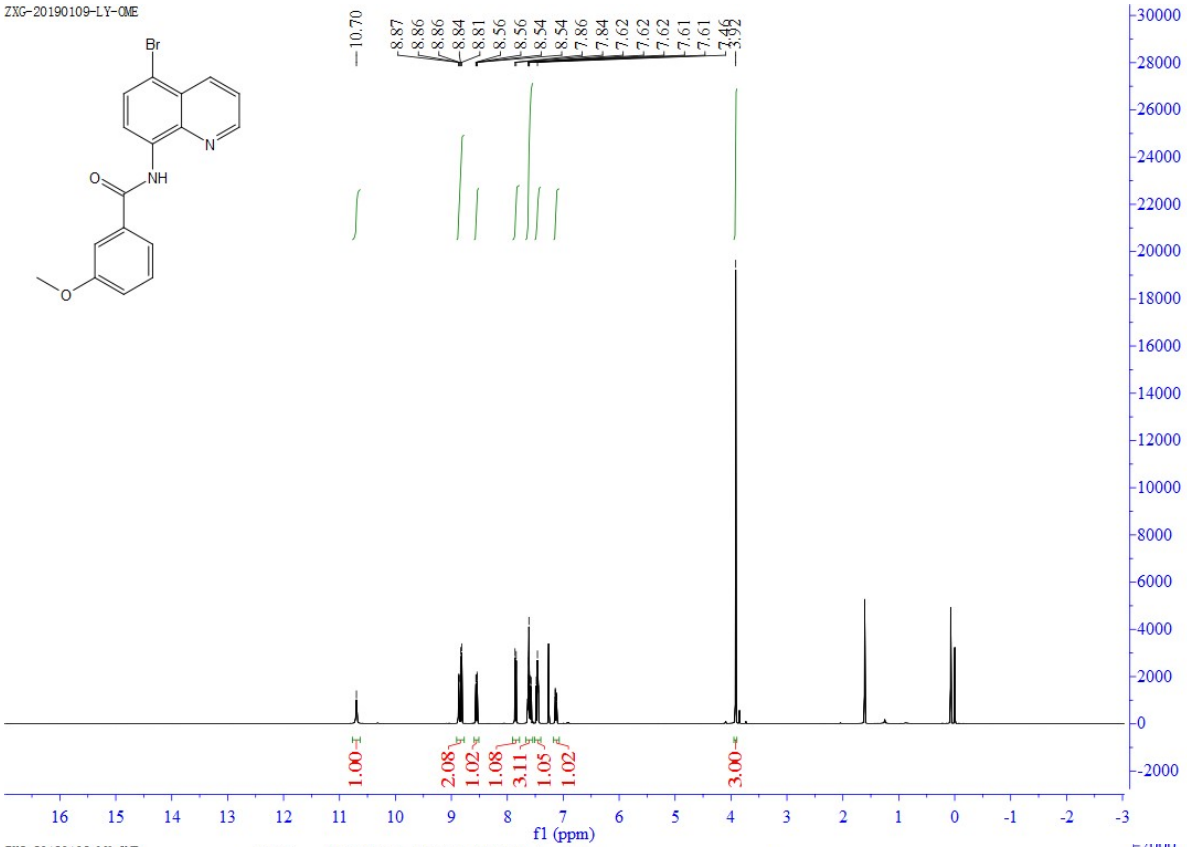

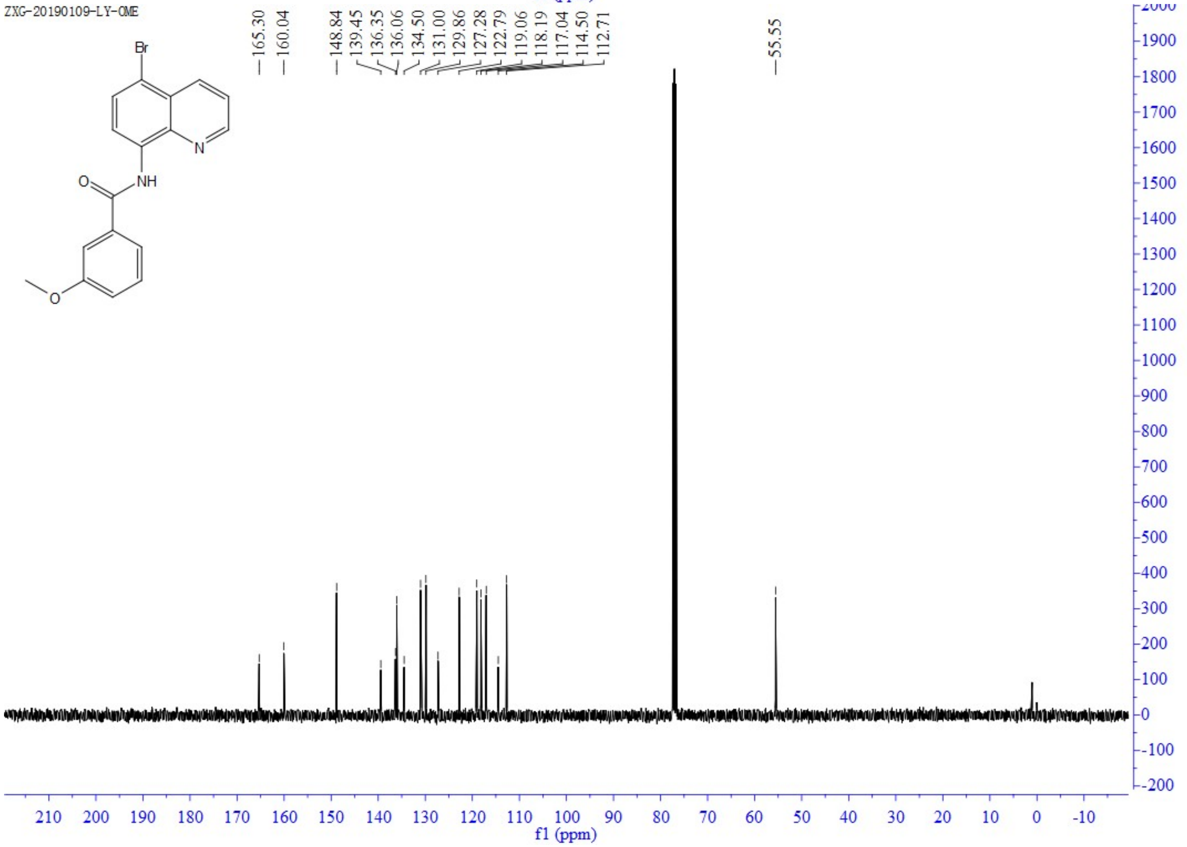

N-(5-bromoquinolin-8-yl)-3-phenylpropanamide (2q)

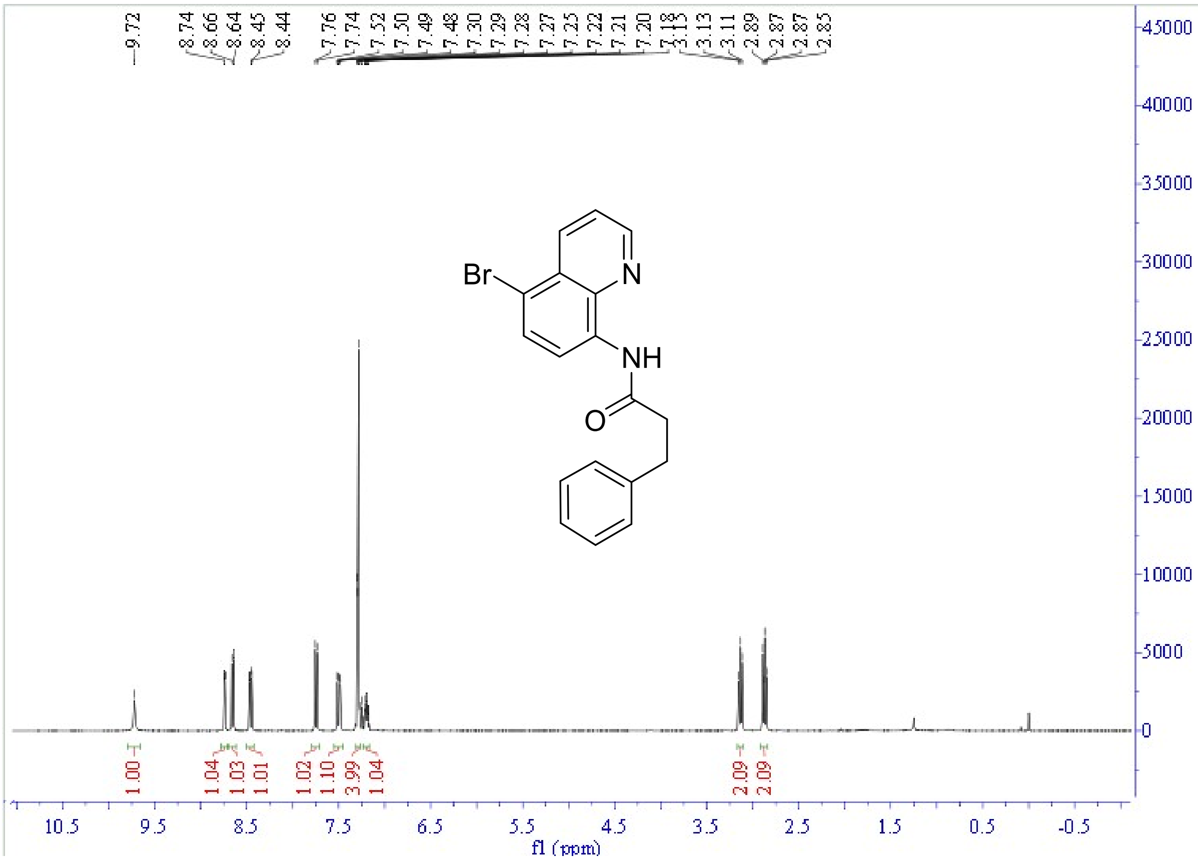

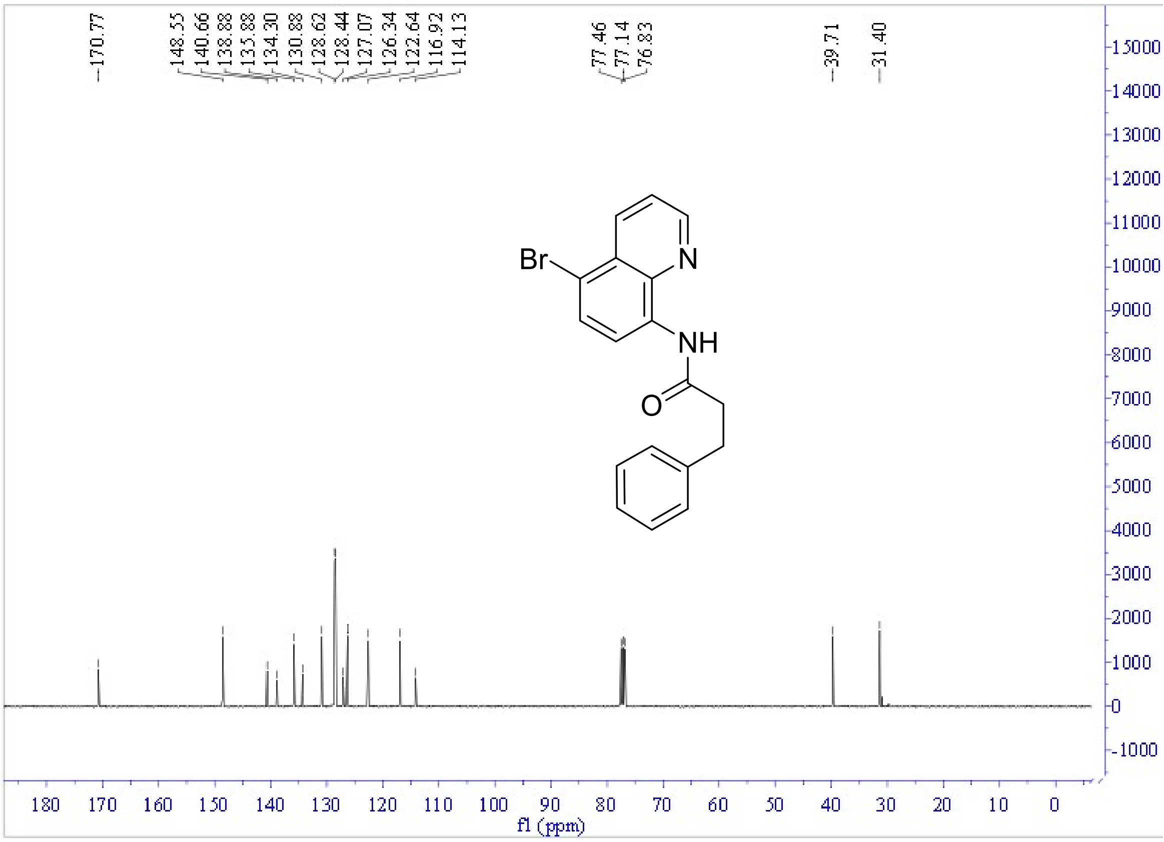

N-(5-bromoquinolin-8-yl)thiophene-2-carboxamide (2r)

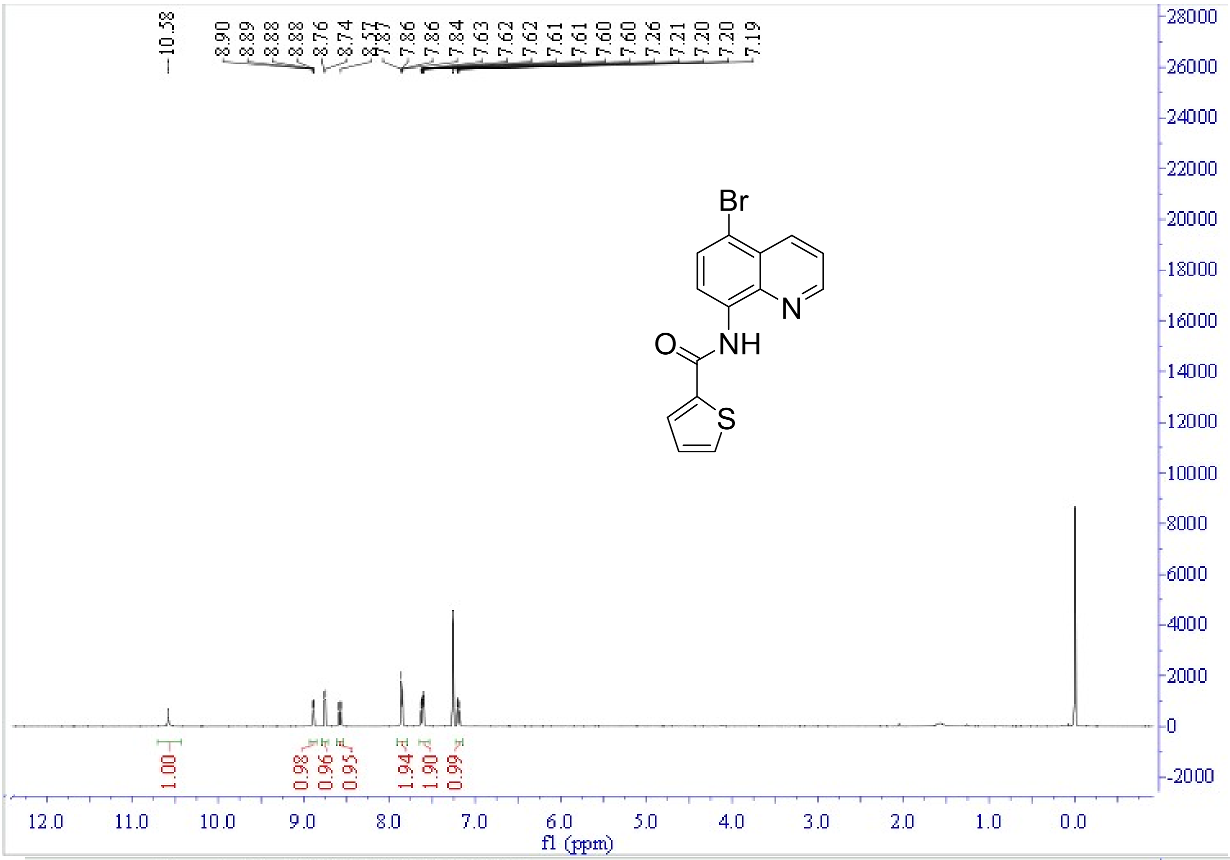

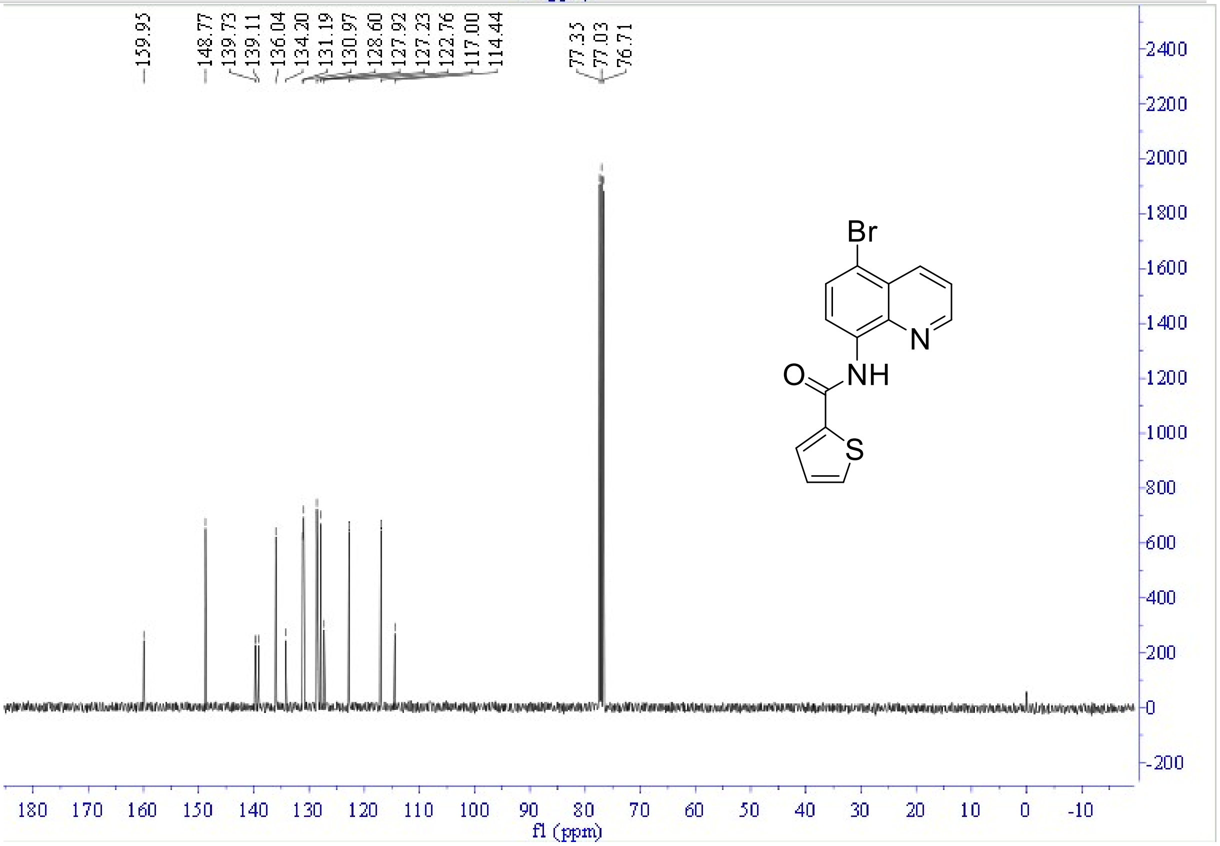

N-(5-bromo-2-methyl-8-quinolinyl)- pivalamide (2s)

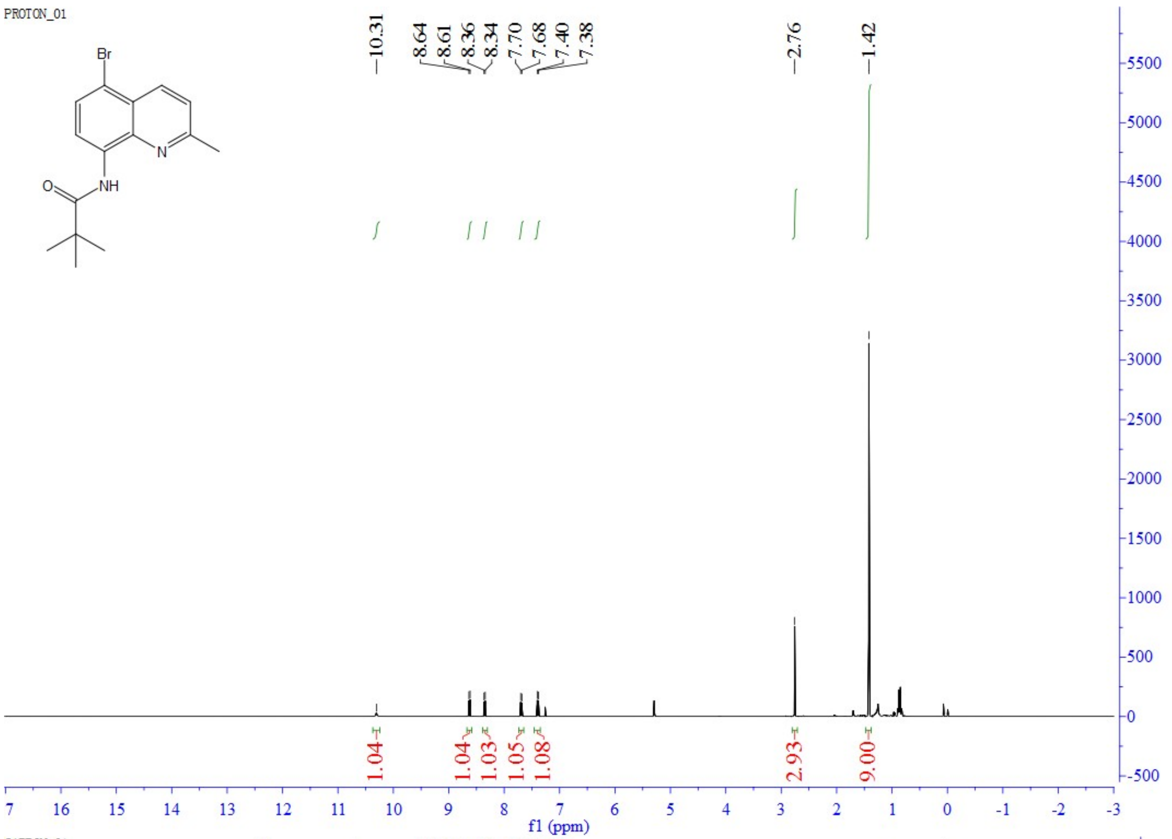

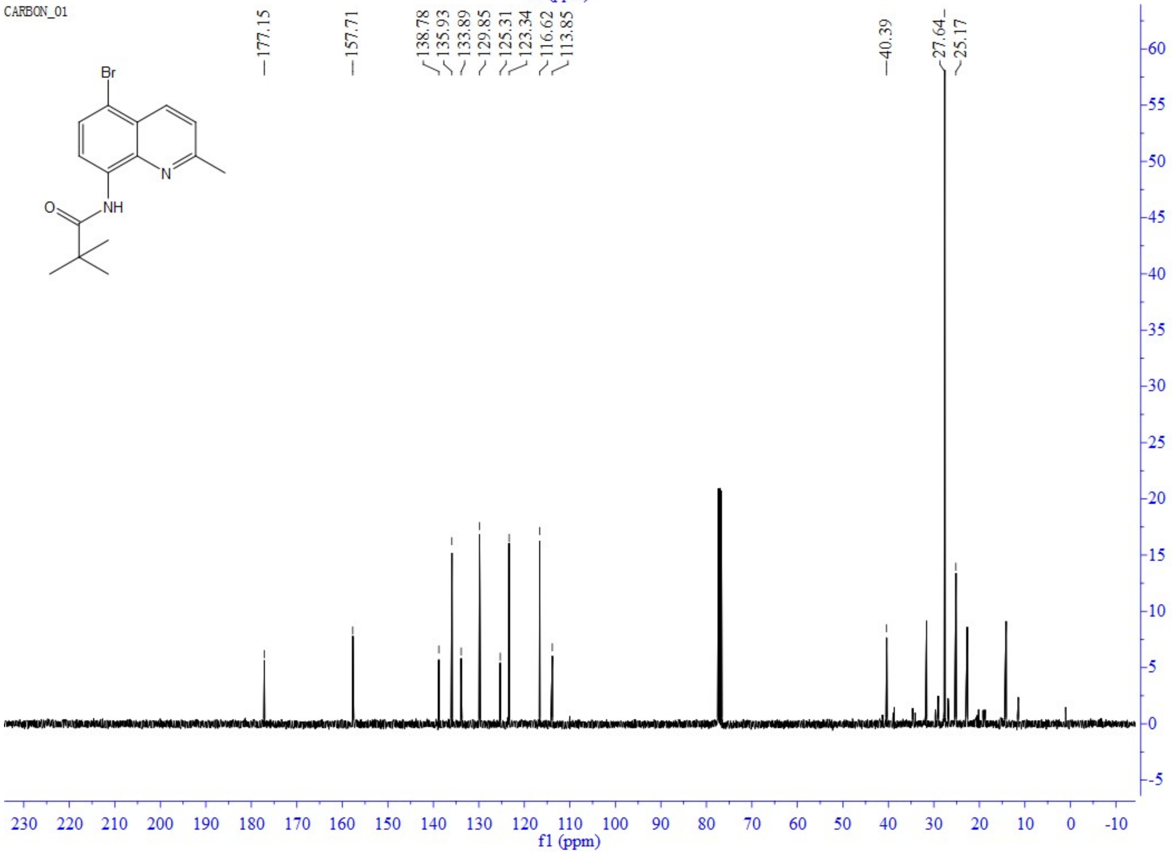

N-(5-iodoquinolin-8-yl)butyramide (3c)

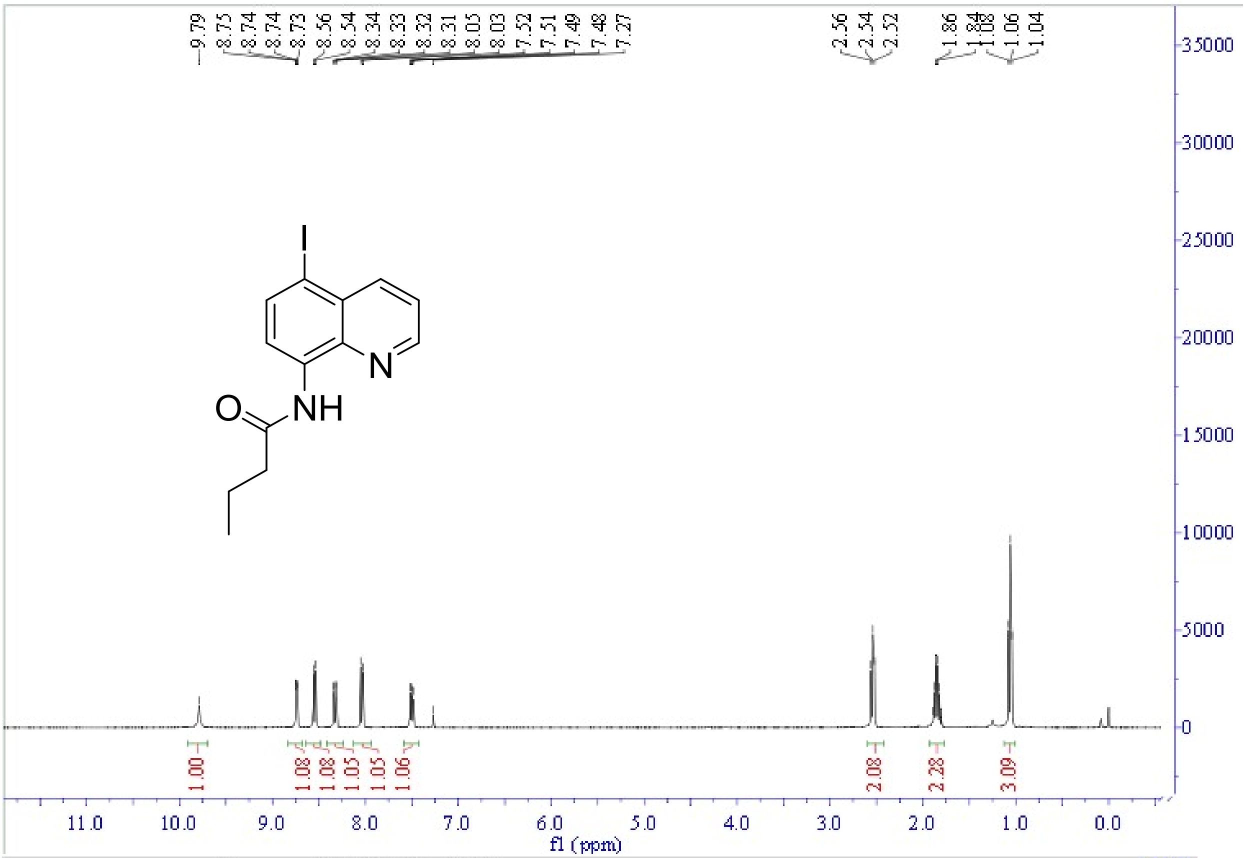

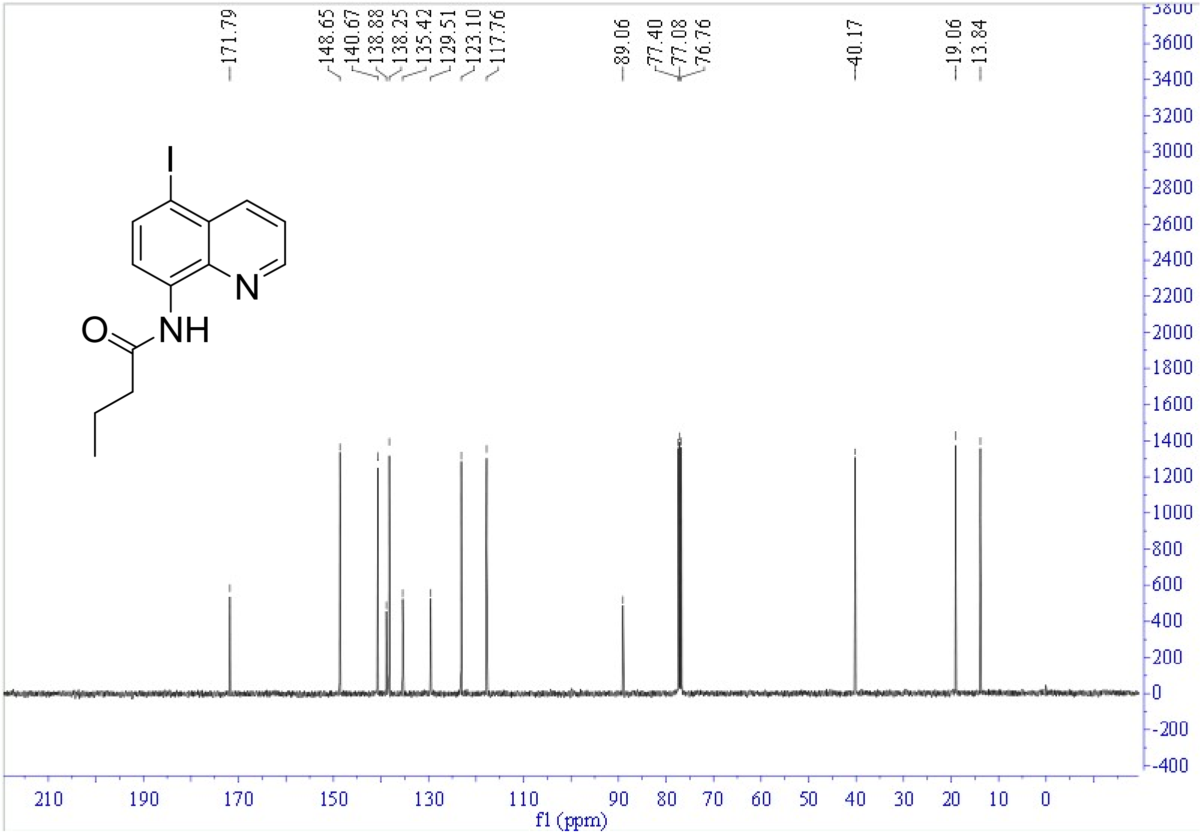

N-(5-iodoquinolin-8-yl)pivalamide (3f)

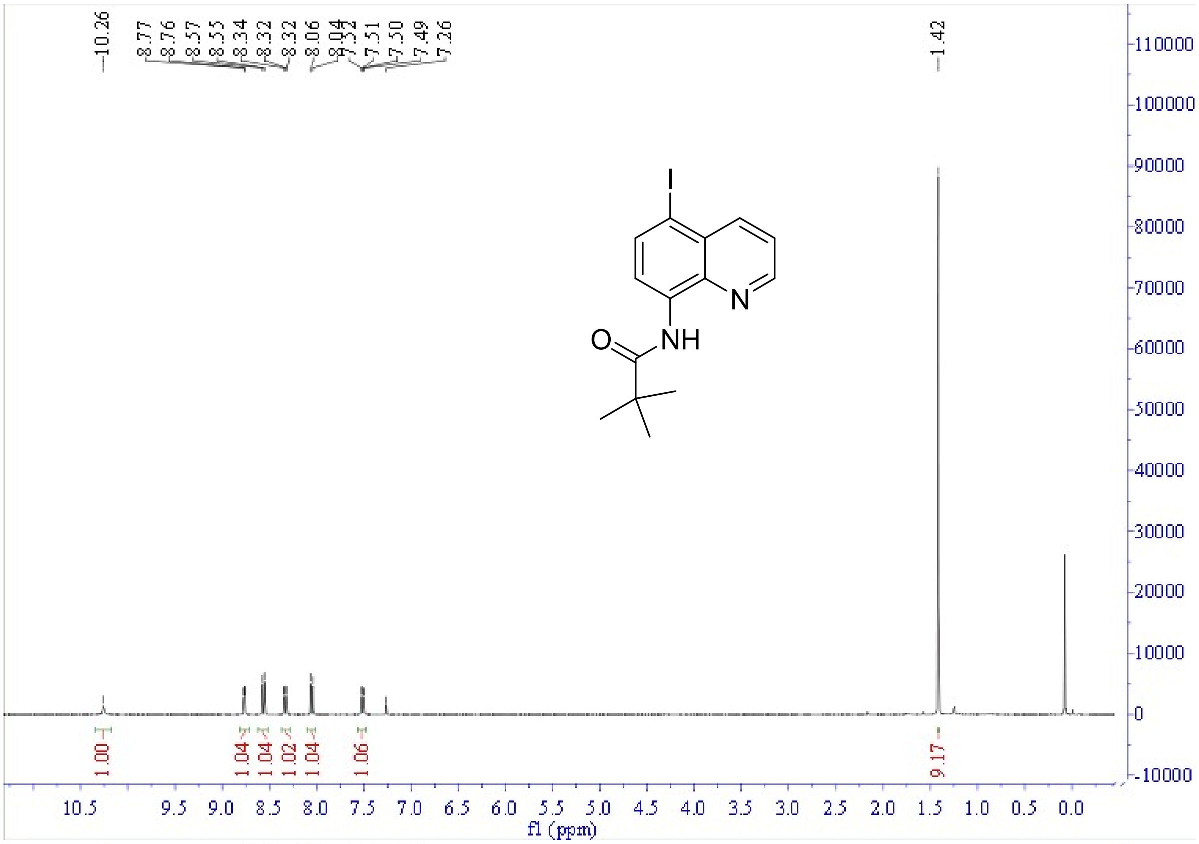

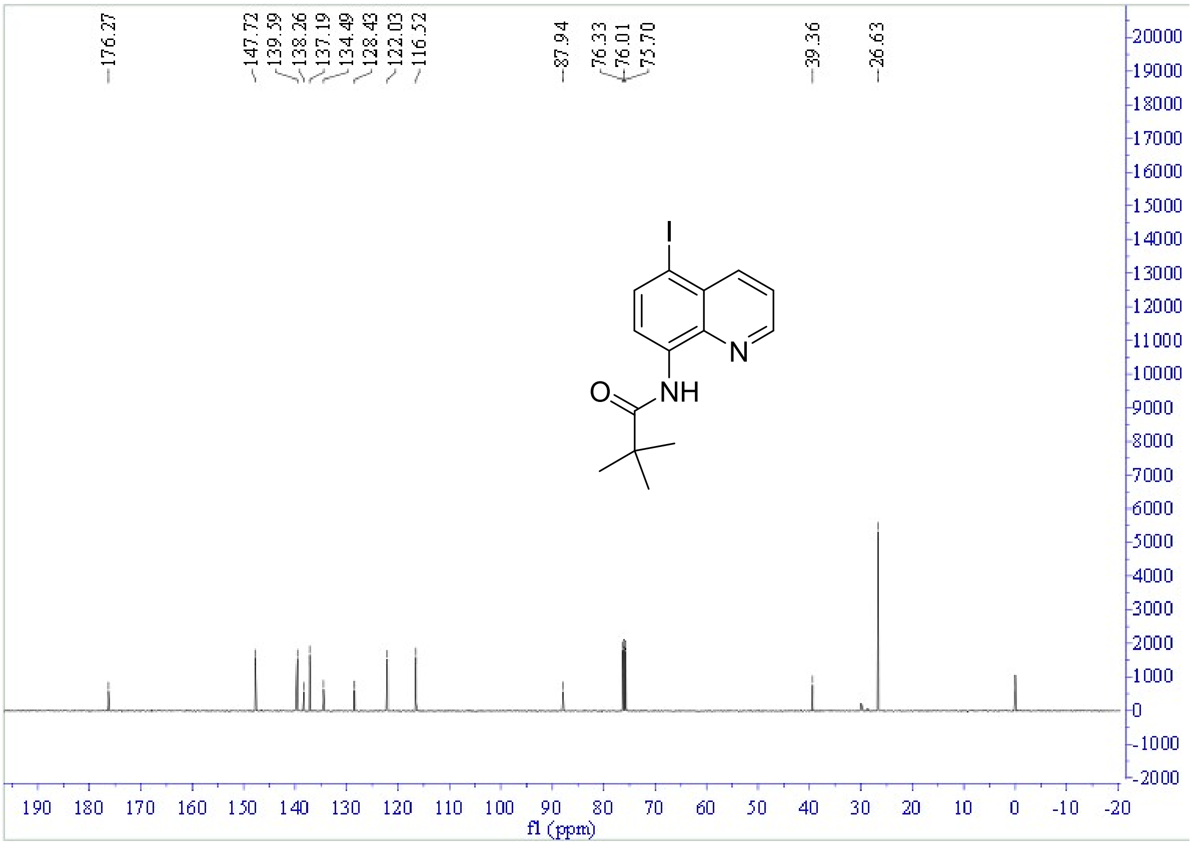

N-(5-phenylquinolin-8-yl)pivalamide (4a)

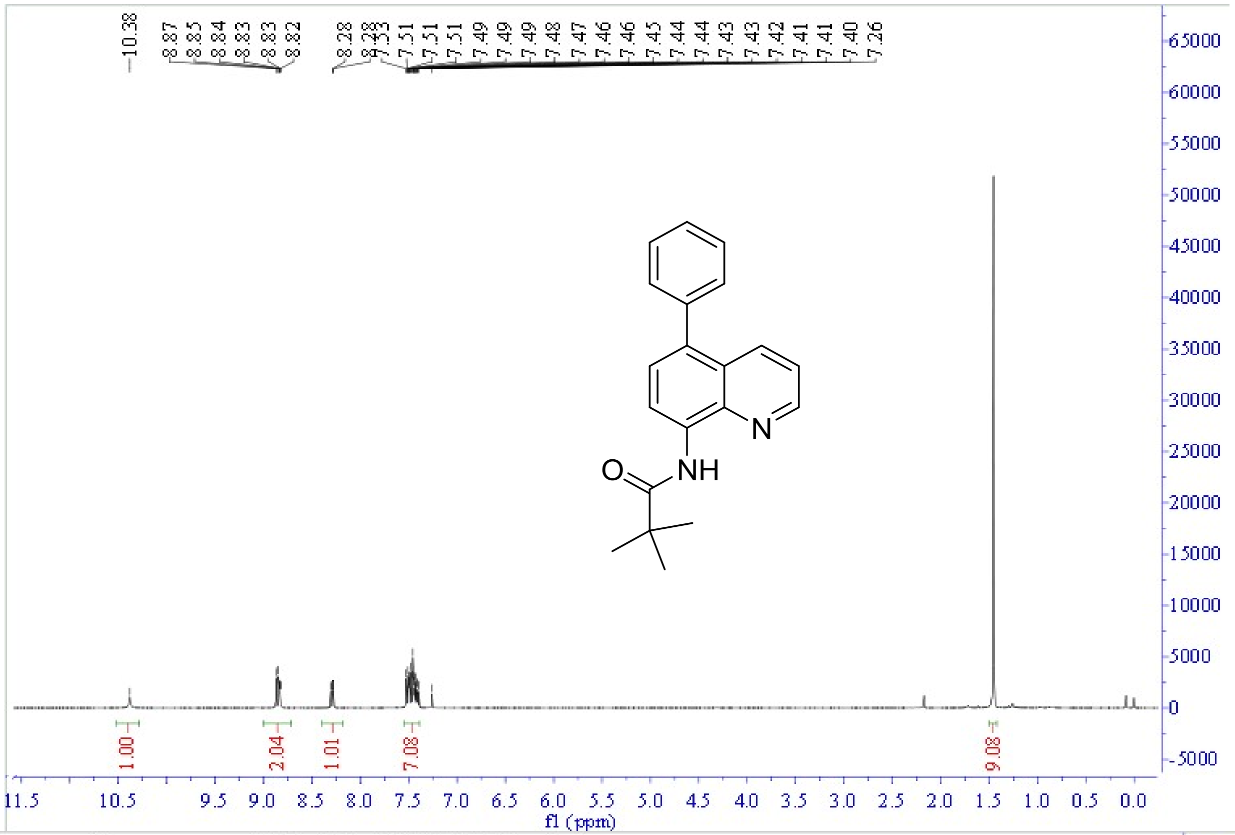

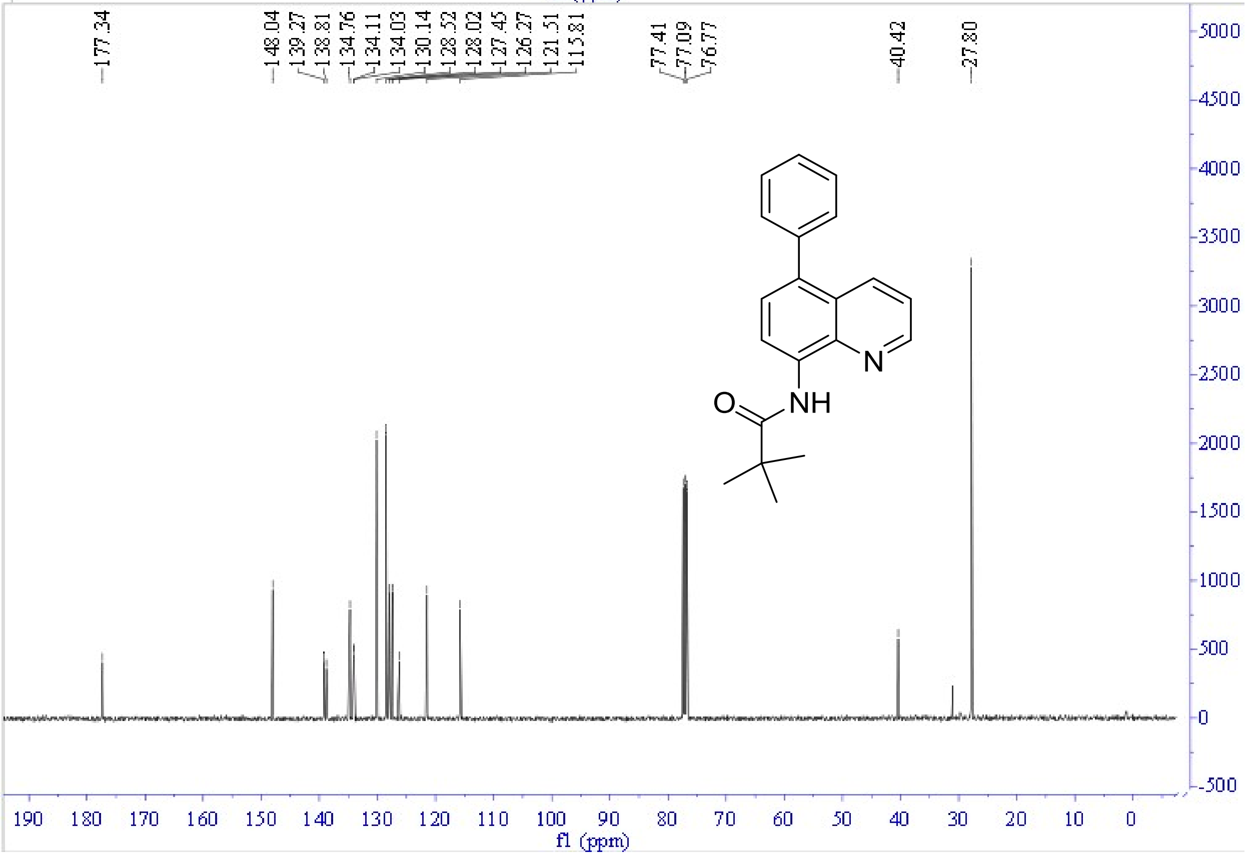

N-(5-(4-methoxyphenyl)quinolin-8-yl)pivalamide (4b)

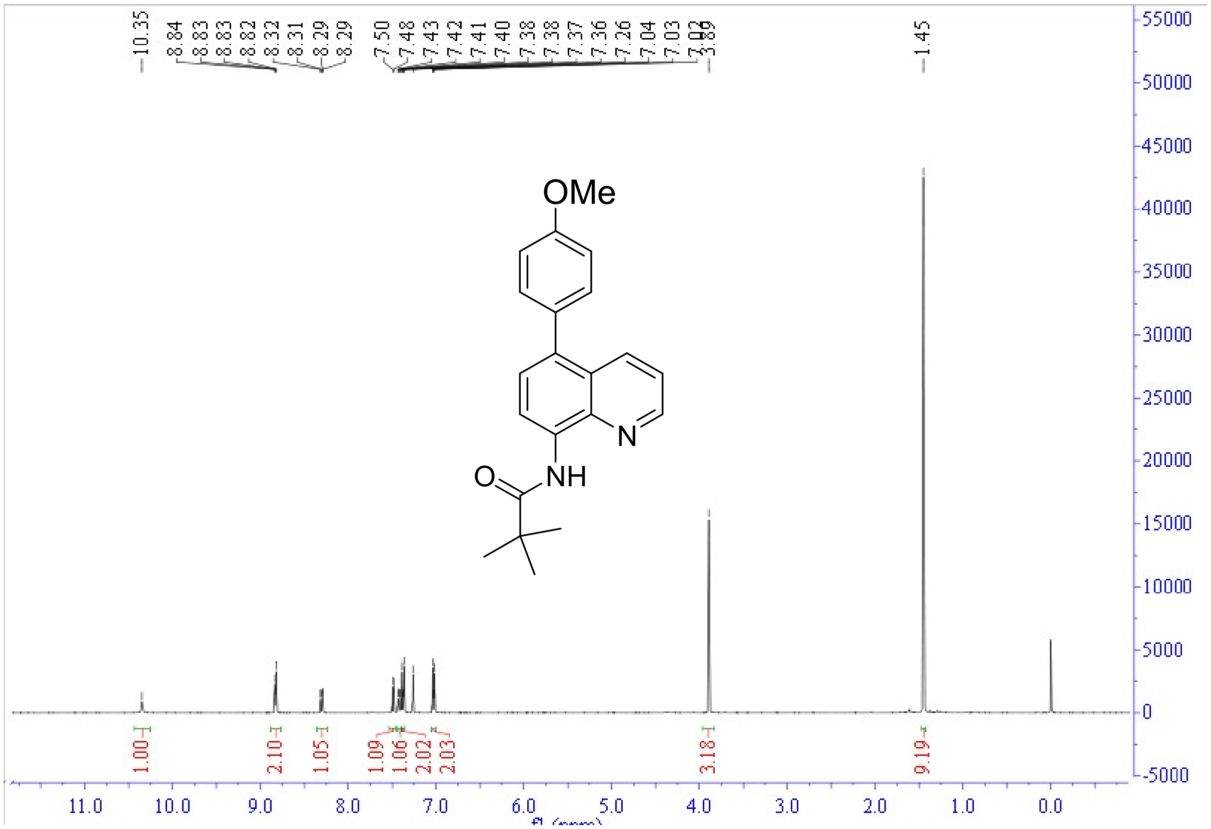

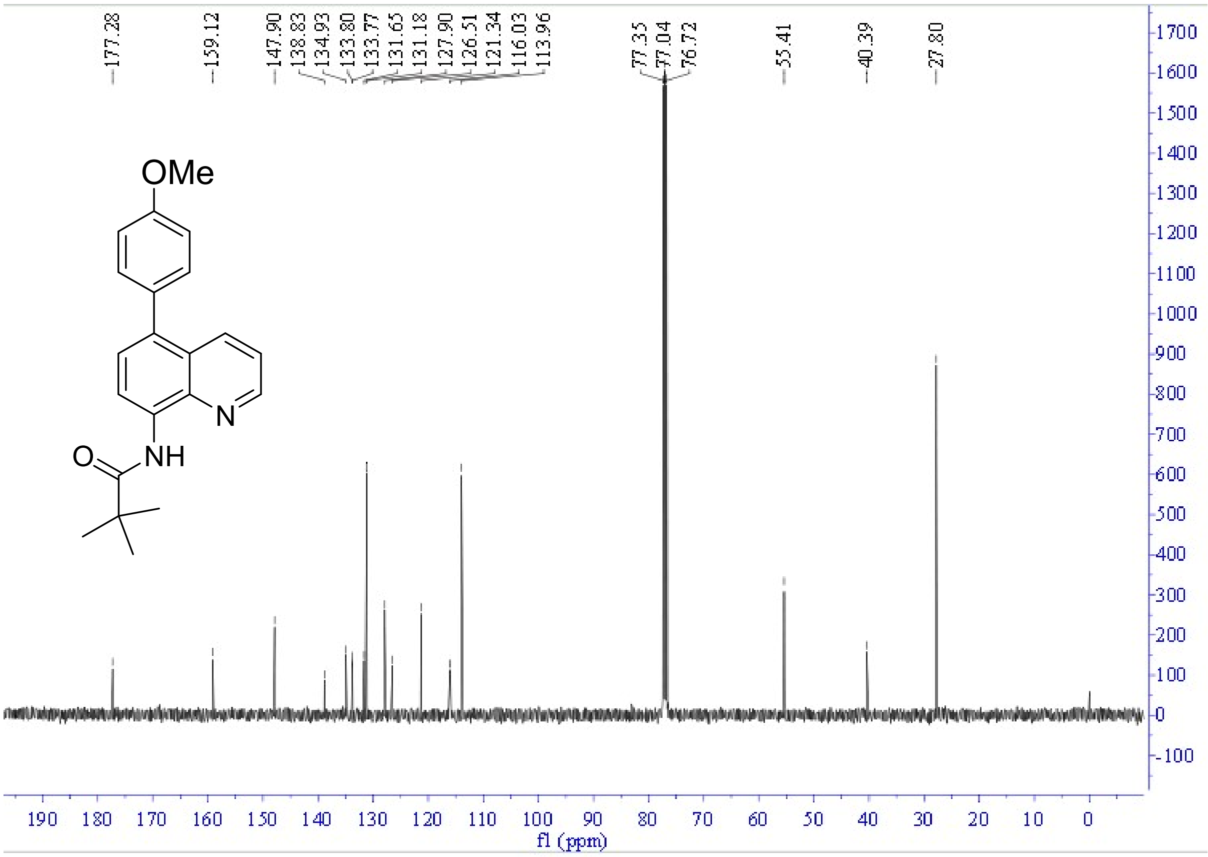

N-(5-(pent-1-en-1-yl)quinolin-8-yl)pivalamide (4c)

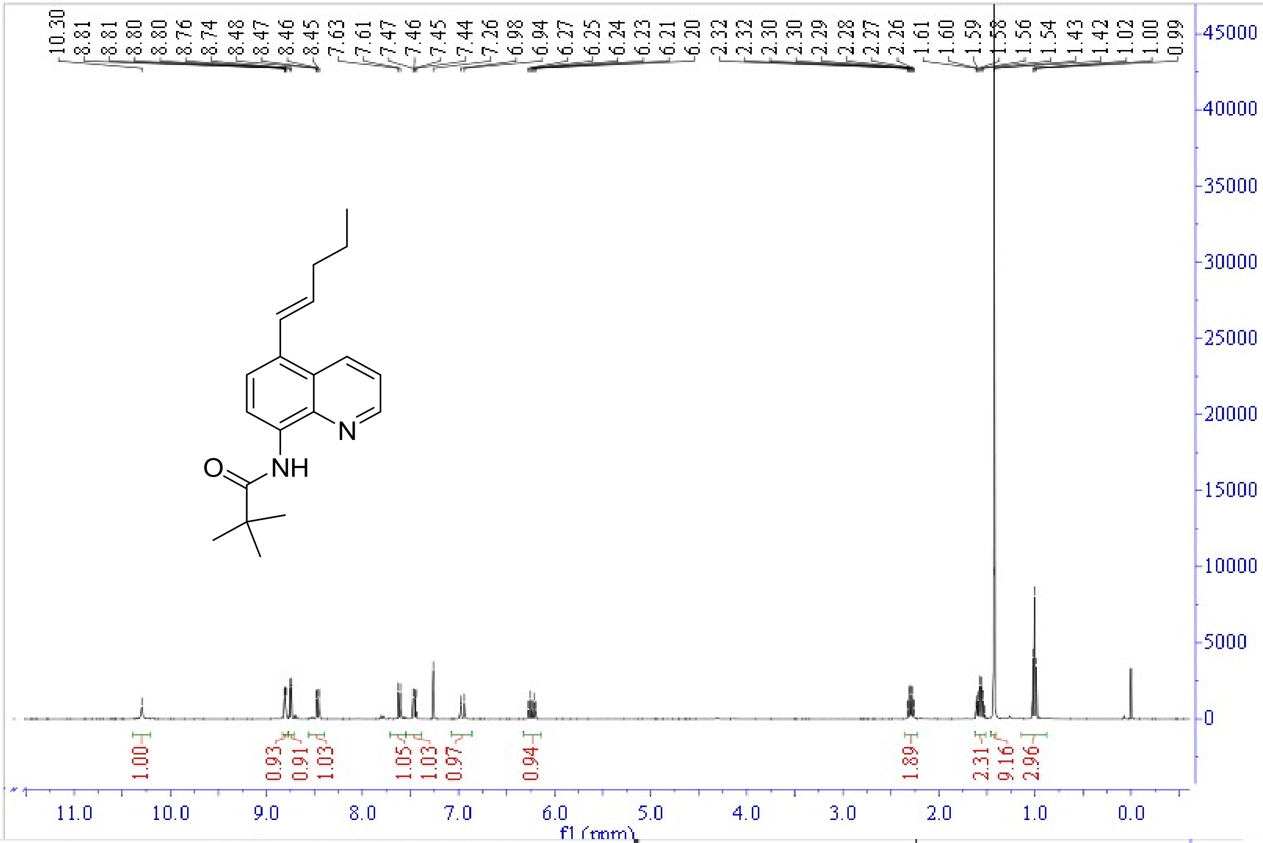

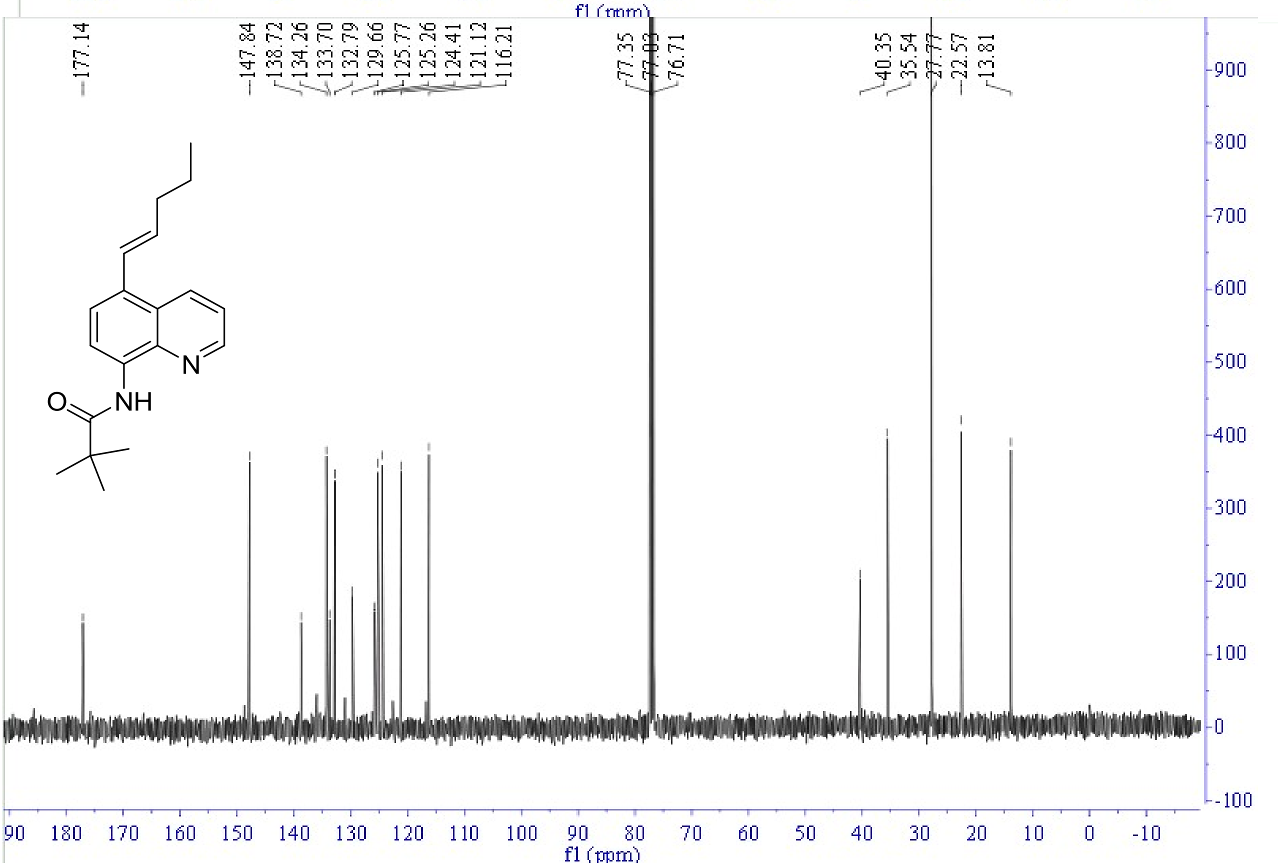

N-(5-(4-chlorophenyl)quinolin-8-yl)pivalamide (4d)

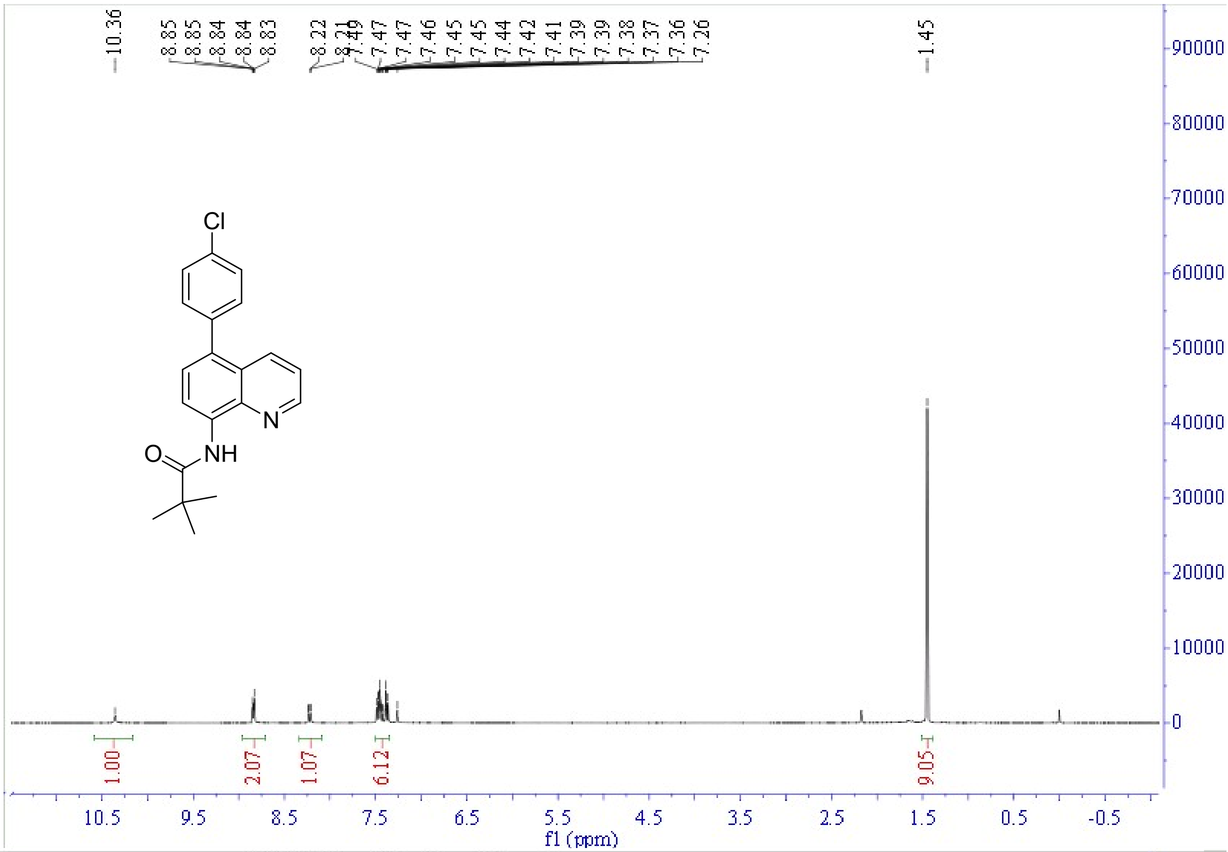

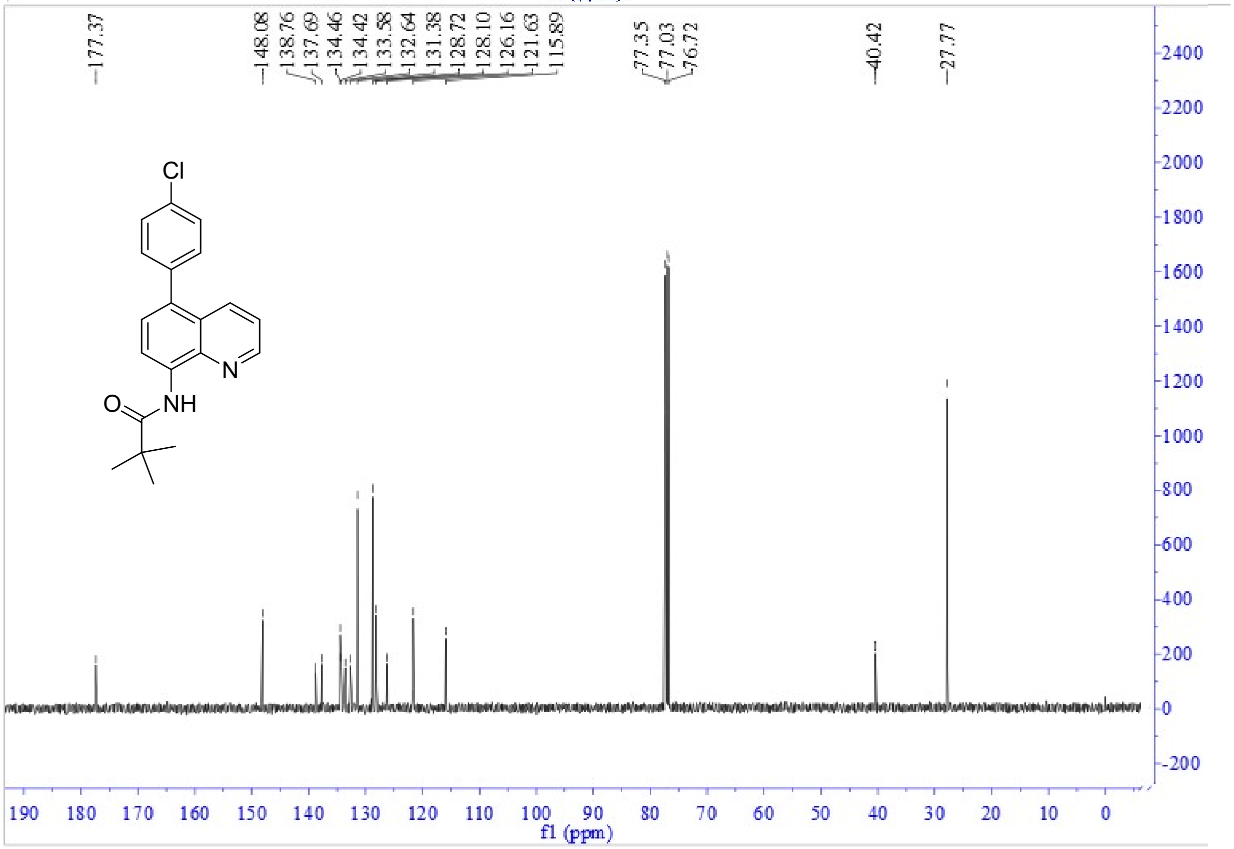

N-(5-(4-cyanophenyl)quinolin-8-yl)pivalamide (4e)

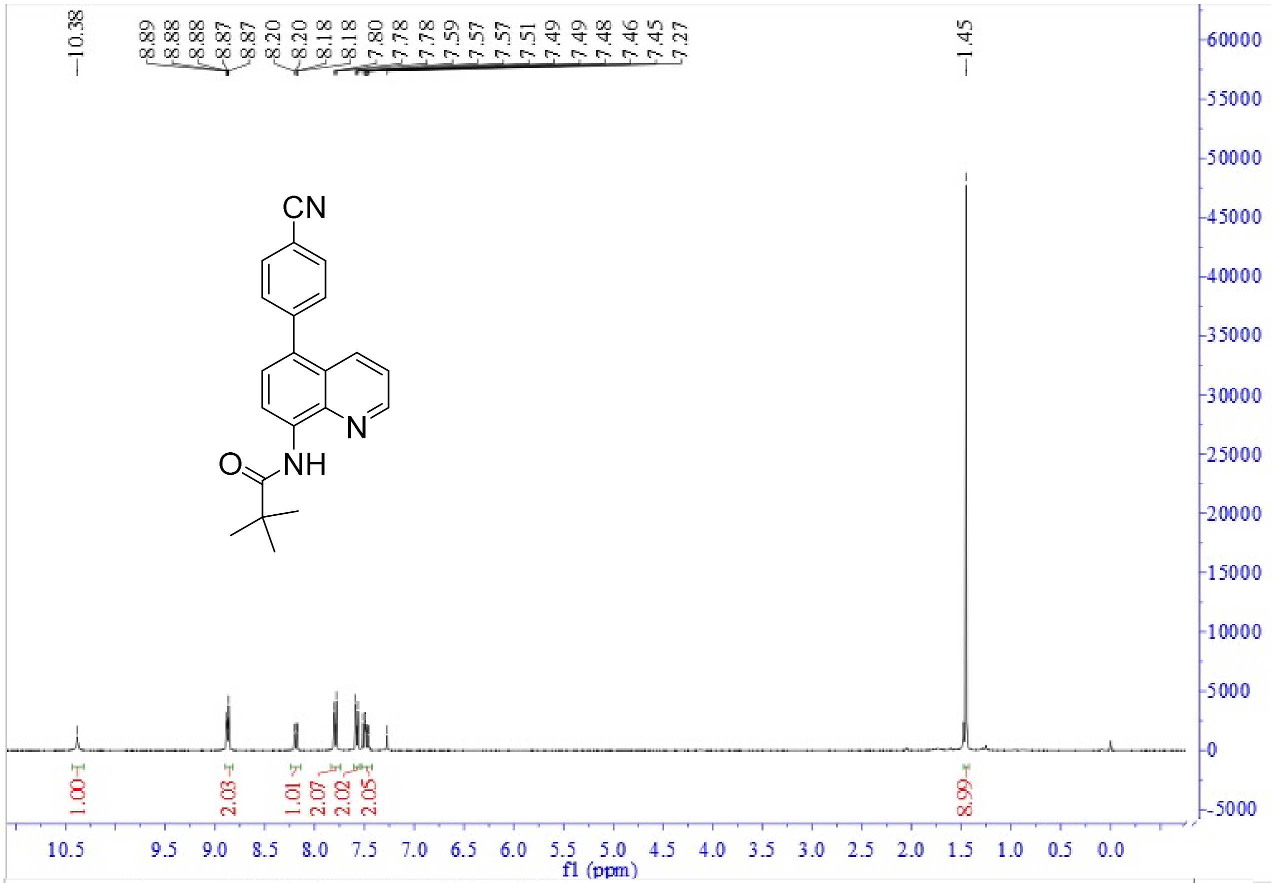

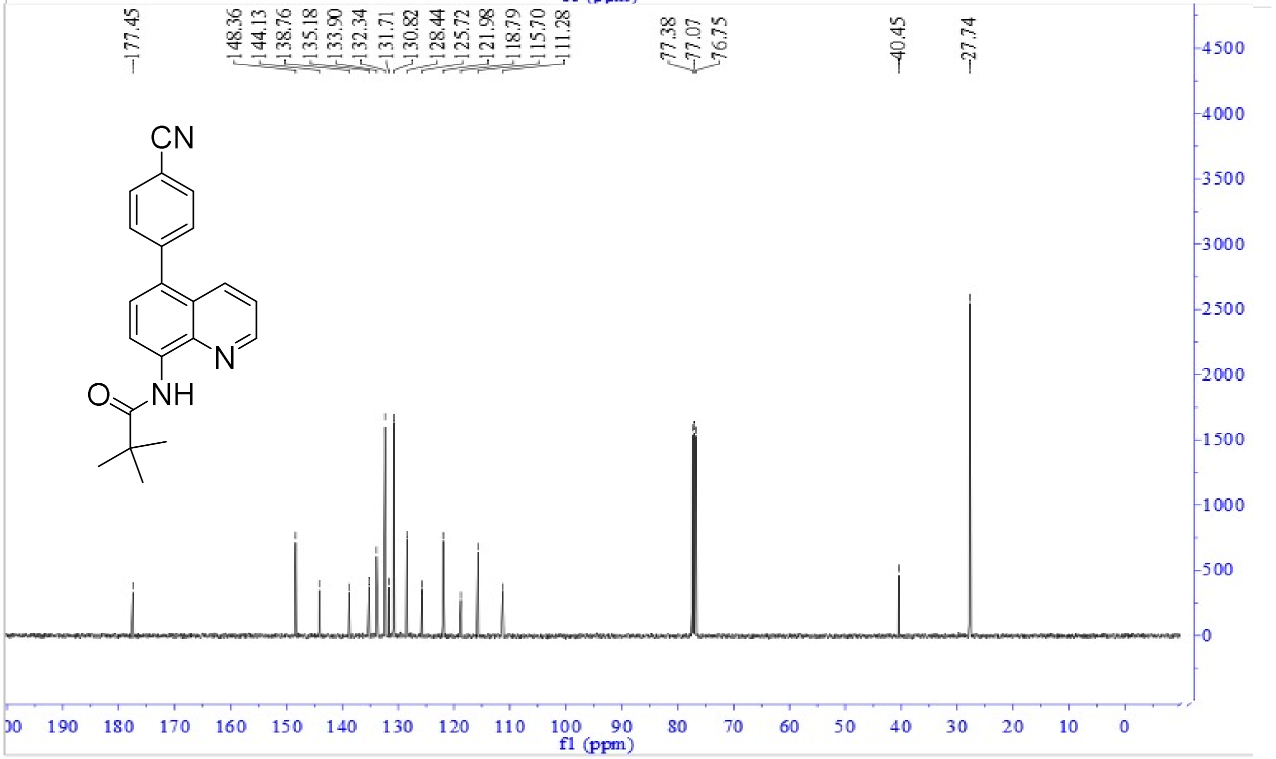

Supplement: Supplementary file 1 [file molecules-24-00535-s001.pdf]
